# Biocontrol potential of *Pseudomonas protegens* ML15 against *Botrytis cinerea* causing gray mold on postharvest tomato (*Solanum lycopersicum* var. *cerasiforme*)

**DOI:** 10.3389/fpls.2023.1288408

**Published:** 2023-12-07

**Authors:** Nur Ajijah, Angelika Fiodor, Mikolaj Dziurzynski, Robert Stasiuk, Julia Pawlowska, Lukasz Dziewit, Kumar Pranaw

**Affiliations:** ^1^Department of Environmental Microbiology and Biotechnology, Institute of Microbiology, Faculty of Biology, University of Warsaw, Warsaw, Poland; ^2^Institute of Biochemistry and Biophysics, Polish Academy of Sciences, Warsaw, Poland; ^3^Department of Biology (DBIO), University of Florence, Sesto Fiorentino, Florence, Italy; ^4^Department of Geomicrobiology, Institute of Microbiology, Faculty of Biology, University of Warsaw, Warsaw, Poland; ^5^Institute of Evolutionary Biology, Faculty of Biology, Biological and Chemical Research Center, University of Warsaw, Warsaw, Poland

**Keywords:** biocontrol, *Botrytis cinerea*, postharvest fruits, *Pseudomonas protegens*, gray mold

## Abstract

Gray mold, caused by *Botrytis cinerea* is a major cause of post-harvest rot of fresh fruits and vegetables. The utilization of selected microorganisms as biocontrol agents is a promising alternative to effectively control gray mold on tomatoes. The current study was conducted to explore potential biocontrol mechanisms of the *Pseudomonas* strain to control infections on post-harvest tomatoes. Among the 8 tested bacterial isolates, *Pseudomonas protegens* ML15 demonstrated antagonistic activity to *Botrytis cinerea*. Moreover, *P. protegens* ML15 exhibited the production of siderophores, hydrogen cyanide, ammonia, exopolysaccharides, lipase, biosurfactant, 2,4-diacetylphloroglucinol, and several other antifungal compounds, such as 1-tetradecanol, cyclododecane, 2,4-di-tert-butylphenol, and 2-methyl-1-hexadecanol. A comprehensive genomic analysis of *P. protegens* ML15 unravels 18 distinct genetic regions with the potential for biosynthesizing secondary metabolites, known for their pivotal role in biocontrol responses against plant pathogens. *In vivo*, experiments showed that both culture suspension and cell-free supernatant of *P. protegens* ML15 significantly reduced fungal growth (53.0 ± 0.63%) and mitigated disease development (52.8 ± 1.5%) in cherry tomatoes at four days post-*B*. *cinerea* inoculation. During the infection, the application of *P. protegens* ML15 resulted in the augmentation of total antioxidant, phenolic content, and ascorbic acids content. Thus, our results suggested that *P. protegens* ML15’s role as a biocontrol agent against *B. cinerea*-induced postharvest tomato decay achieved through the secretion of antifungal substances, induction of tomato defense responses, and inhibition of mycelial growth of *B. cinerea*. These findings provide a significant contribution to the ongoing search for alternative, eco-friendly methods of controlling gray mold in fresh products. The utilization of *P. protegens* ML15 as a biocontrol agent could help to reduce the reliance on chemical fungicides and promote sustainable agriculture practices.

## 1 Introduction

Fungi that cause plant diseases contribute to approximately 20-25% of the total losses attributed to decay in harvested fruits and vegetables (Petrasch et al., 2019). Most postharvest pathogens have a necrotrophic or saprotrophic lifestyle that causes the decomposition of host tissues. As a result, fruits and vegetables lose nutritional value and organoleptic appeal, ultimately leading to large economic deprivation (Jiménez-Reyes et al., 2019; Petrasch et al., 2019).

Members of the *Botrytis* genus are generally, ascomycetous, plant pathogenic anamorphic fungi. *Botrytis cinerea* causes gray mold disease that can infect leaves, stems, flowers, and fruits. This necrotrophic fungus has a wide range of hosts, more than 1400 plant species, including economically important crops such as tomatoes, strawberries, pears, cherries, eggplants, grapes, and peppers (Abbey et al., 2019; Poveda et al., 2020). It is estimated that gray mold is the second leading cause of annual losses in harvested fruits and vegetables worldwide, ranging from $10 to $100 billion (Hua et al., 2018; Bu et al., 2021). *B. cinerea* is capable of rotting tomato fruit by direct entry or injury after pruning and harvesting. Tomato gray mold causes severe economic and nutritional deficits worldwide due to the common use of tomatoes in many food products and their retail sale (Sarven et al., 2020). In addition, inadequate processing, storage, packaging, or marketing contribute to rot losses (Bu et al., 2021). Combating this pathogenic fungus is challenging due to the wide range of hosts, dual infection strategies (pre- and postharvest), and ability to survive in the form of conidia or sclerotia (Poveda et al., 2020). Despite the huge availability of fungicides worldwide, at best 8% of them are used against *B. cinerea*. In addition, only a small fraction of these agents are effective, making arduous fight against this pathogen (De Angelis et al., 2022).

Antimicrobial based biofungicides, unlike chemical fungicides, leave no hazardous residues in the environment, which is consistent approach with food biosecurity and human health (De Angelis et al., 2022; Tang et al., 2022). Few biofungicides have been developed and commercialized for controlling *B*. *cinerea*, such as Trichodex (with *Trichoderma harzianum*), Graygold (with *Trichoderma hamatum, Rhodotorula glutinis*, and *Bacillus megaterium*), Kodiak HB (with *Bacillus subtilis* GB03 and *Bacillus subtilis* QST-713), and BioSave (with *Pseudomonas syringe*) (Jackson, 2014; Abbey et al., 2019). Nonpathogenic beneficial bacteria can be used as antagonists to control pathogenic fungi due to several mechanisms (Lastochkina et al., 2019). Bacteria possess several mechanisms to thwart fungal infections, ranging from competition for essential nutrients and space, synthesizing a diverse array of secondary metabolites with potent antifungal properties, or even activating the host’s defense system (Morales-Cedeño et al., 2021; Zhu et al., 2022). Such mechanisms include the production of various secondary metabolites, such as antibiotics, siderophores, hydrolytic enzymes, hydrogen cyanide, volatile organic compounds, and exopolysaccharides. These complex molecules are believed to play a crucial role in inhibiting fungal growth and development, thus preventing the onset of infections (Ramakrishna et al., 2019; Karthika et al., 2020).

The quest for microorganisms with potent antagonistic activity against *B. cinerea* is a never-ending endeavor in the pursuit of developing innovative biocontrol strategies that foster sustainable and eco-friendly crop production. As the study of bacterial secondary metabolites continues to evolve, it offers boundless possibilities to discover novel and effective means of combatting plant pathogens, while simultaneously promoting environmentally conscious agricultural practices. The development of such biocontrol approaches holds immense promise and presents a transformative shift towards a more sustainable and resilient agricultural system. The biocontrol potential of beneficial *Pseudomonas* spp. has been extensively investigated through antibiosis, competition, and elicitation of induced systemic resistance (ISR) (Balthazar et al., 2022a; Huang et al., 2022). Notably, *Pseudomonas fluorescens* ZX had ability to protect postharvest citrus fruits against blue and green molds, caused by *Penicillium italicum* and *Penicillium digitatum*, respectively (Wang et al., 2021). Additionally, *P. putida* BP25 exhibited robust reduction of anthracnose severity on fruits caused by *Colletotrichum gloeosporioides* (Archana et al., 2021). Moreover, *P. protegens* has demonstrated strong antifungal activity against a wide range of plant pathogens, including *Alternaria alternata, Botrytis cinerea, Botryosphaeria dothidea, Fusarium oxysporum, Heterobasidion abietinum, Sclerotinia sclerotiorum*, and *Verticillium dahliae* (Nandi et al., 2017; Andreolli et al., 2019; Prigigallo et al., 2021; Huang et al., 2022; Ni et al., 2022).

The current study was aimed to achieve a multi-faceted set of objectives. First, to identify a potent bacterial isolate that could effectively suppress the growth of *B. cinerea*, a notorious pathogen responsible for post-harvest gray mold rot in tomatoes. Among the candidates, *Pseudomonas protegens* ML15 exhibited a strong antagonistic activity against *B. cinerea*. Subsequently, the study aimed to explore diverse biocontrol mechanisms employed by the selected isolate, and identify the various antifungal compounds synthesized by it. The study also delved into the biosynthetic potential of the isolate, based on comprehensive genome mining techniques, to unravel the underlying mechanisms responsible for its potent biocontrol activities. Finally, the study evaluated the biological control efficacy of the promising isolate against *B. cinerea*-caused post-harvest gray mold rot, thereby offering a comprehensive insight into the potential biocontrol mechanisms of the *Pseudomonas* strain in mitigating the harmful impact of *B. cinerea* infections on post-harvest tomatoes.

## 2 Materials and methods

### 2.1 Microbial cultures

A total of 8 plant growth promoting bacteria (PGPB) isolates from our previous study were used in this study (Fiodor et al., 2023). Two (*Bacillus thuringiensis* EDC1 and *Serratia plymuthica* EEPC5) bacterial isolates were previously isolated from carrot and parsley root samples, one (*Pseudomonas protegens* ML15) from Błędowska Desert and five (*Burkholderia ambifaria* AF8II10, *Bacillus cereus* AF8II13, *Klebsiella aerogenes* AF3II1, *Pseudomonas putida* AF1I1, *Serratia marcescens* AF8I1) from rhizospheric soil samples of cereal crops from Hołodolina, Niewodnica Nargilewska, and Hermanówka. The bacterial culture was maintained on Luria Bertani (LB) agar medium. The fungal phytopathogen *Botrytis cinerea* BC21 was obtained from the culture collection of Institute of Evolutionary Biology, University of Warsaw. *B. cinerea* BC21 was isolated from strawberry fruit and maintained on Potato Dextrose Agar (PDA) medium at 20°C for 5 - 7 days for subsequent tests.

### 2.2 Antagonistic activity of bacterial isolates against *B. cinerea* BC21

Bacterial isolates were investigated for their potential antagonistic activity against *B. cinerea* BC21 using both dual culture growth inhibition and agar well diffusion methods (Guo et al., 2020; Jia et al., 2020). Plates were kept at 25°C until the mycelia of tested fungi were fully grown in the control plate. The inhibition percentage was calculated according to Ghazy and El-Nahrawy (2021).


Eq. (A) $$Inhibition\;percentage=\left(\frac{C-T}{C}\right)x100$$

Where, C is the radial growth of fungi in control, and T is the radial growth of the fungi in the presence of the bacterial isolate.

Whereas, the diameter of the inhibition zone (mm) around the well was measured to evaluate antagonistic activity of antimicrobial compound in cell-free supernatant of bacterial isolates (Jia et al., 2020).

### 2.3 Traits related to biocontrol activity of selected isolate against *B. cinerea* BC21

One bacterial isolate was selected on the basis of antagonistic activity against *B. cinerea* BC21. Selected isolate was investigated for several indirect mechanisms that may inhibit phytopathogens, including the production of siderophores, hydrogen cyanide, exopolysaccharides, ammonia, extracellular enzymes, and biosurfactant.

#### 2.3.1 Siderophores production

The ability of bacteria to produce siderophores was qualitatively determined using Chrome Azurol S (CAS) assay (Pranaw et al., 2020). Briefly, freshly grown bacterial cultures were spot inoculated onto CAS agar medium. Siderophore production was confirmed by the formation of an orange halo zone around a colony after 5 d of incubation at 30°C. The diameter of colored zone was used to determine siderophores production index (SPI) according to Tamariz-Angeles et al. (2021).


Eq. (B) $$SPI=\frac{diameter\;of\;orange\;halo\;zone}{diameter\;of\;colony}$$

#### 2.3.2 Hydrogen cyanide production

HCN production was examined using method proposed previously (Pranaw et al., 2020). Freshly grown inoculum of bacteria was streaking on King’s B agar medium supplemented with 4.4 g L^-1^ of glycine. A layer of Whatman No. 1 filter paper (90 mm) was soaked in 0.5% picric acid solution in 2% sodium carbonate and placed in the lid of each Petri plate. All plates were sealed with parafilm and incubated at 30°C for 4 d. Hydrogen cyanide production was confirmed by the change in color of the filter paper from yellow to orange-brown.

#### 2.3.3 Exopolysaccharides production

EPS production was estimated using methodology described by Oleńska et al. (2021). Bacterial isolates were cultured in 40 mL of LB broth medium for 5 d (30°C, 150 rpm). Subsequently, cultures were centrifuged at 12,857× *g* for 30 min at 4°C. Each supernatant was mixed with two volumes of absolute ethanol and kept for 24 h at 4°C for precipitation. Crude EPS was weighted after centrifugation at 12,857× *g* for 30 min and dried overnight at 50°C.

#### 2.3.4 Ammonia production

Production of ammonia was determined by method described by Mukherjee et al. with some modifications (Mukherjee et al., 2019). Fresh grown culture (OD600~ 0.8–1.2) of each bacterial isolate was inoculated in 40 mL of peptone broth and incubated for five days with rotational shaking (150 rpm) at 30°C. Bacterial cultures were collected in Eppendorf tubes and centrifuged at 12,857× *g* for 5 min. Subsequently, 40 µL of K-Na tartrate and 40 µL of Nessler’s reagent were added to 2 mL of supernatant. The formation of ammonia was indicated by a color change from yellow to dark brown. Finally, OD was measured at 435 nm by spectrophotometer (Evolution 201, Thermo Scientific, Madison, USA), and based on the standard curve of ammonium sulphate ((NH_4_)_2_SO_4_), the ammonia concentration was calculated.

#### 2.3.5 Extracellular enzyme production

The ability to produce cellulase, protease, lipase, and chitinase was tested using four different solid media. Glucose-yeast-peptone medium supplemented with 0.5% carboxymethyl cellulose was used to test cellulose production ability. Plates were incubated for 3 d, then flooded with 0.1% Congo Red, and then distained with 1 M NaCl (Saad et al., 2020). Glucose yeast peptone medium with 1% gelatin was used to test protease synthesis ability (De Veras et al., 2018). Lipase production ability was assessed using peptone agar with 1% Tween 20 (Sigma-Aldrich, St. Louis, USA) (Saad et al., 2020). For the evaluation of the chitinolytic properties, chitinase detection agar medium containing 10% of the colloidal chitin prepared from commercial chitin (Sigma-Aldrich, St. Louis, USA) according to Drewnowska et al. (2020) was used. Briefly, a 2-d-old liquid bacterial culture was spot inoculated (5 µL) onto the surface of a specific agar medium. The plates were incubated at 30°C for 2-5 d to observe a clear zone or hydrolysis area around the colonies, and then the enzymatic index was calculated.


Eq. (C) $$\text{Enzymatic index}=\frac{\text{diameter of hydrolysis area}}{\text{diameter of colony}}$$

#### 2.3.6 Biosurfactants production

Emulsifying activity of the biosurfactants produced by selected isolate was determined by the measurement of the emulsion index (E_24_). Cell-free supernatant (CFS) was collected from 3-d-old liquid bacterial culture grown in a minimal salt medium. Briefly, equal volumes of hexadecane and cell-free supernatant in a clean test tube was mixed by vortexing for 2 min, and kept for 24 h at room temperature. E_24_ was calculated according to Sharma and Pandey (2020).


Eq. (D) $$E_{24}=\frac{\text{height of the emulsion layer }}{\text{height of the total solution}}\text{x }100$$

#### 2.3.7 Effects of volatile organic compounds (VOCs) produced by the selected bacterial isolate in inhibiting mycelia growth of *B. cinerea*

The antifungal activities of VOCs emitted by the selected isolate was investigated using the double plate method according to Li et al. (2022). Selected isolate was streaked onto one base of the plate containing of LB agar medium and incubated for 1 d at 30°C. Subsequently, a mycelial disk (5 mm in diameter) was placed onto another plate containing PDA medium. The two base plates were tightly sealed with parafilm and incubated at 25°C for 7 d. The two base plates with uninoculated bacterial isolate were used as a control. The inhibition percentage was calculated as described earlier (Eq. A). Each treatment was performed in three replicates.

### 2.4 Extraction and identification of antifungal metabolites in a cell-free extract

The thin layer chromatography (TLC) method was used to separate compounds in cell-free an extract. Bacterial culture was grown for three days at 30°C, 150 rpm and then was centrifuged at 12,857× *g*, 10 min, 4°C. Therefore, the supernatant was extracted three times with an equal volume of ethyl acetate. The organic fraction was dried with Na_2_SO_4_ and evaporated using a rotary evaporator (Trejo-Raya et al., 2021). The resulting cell-free extract was used for further analysis. The content of phloroglucinol in cell-free extract was detected by TLC using hexane: acetone (3:2 v/v) as solvent systems (Trejo-Raya et al., 2021).

High-performance liquid chromatography (HPLC) method was used for quantification of 2,4-diacthylphloroglucinol (2,4-DAPG) in CFS of selected isolate. The CFS was filtered through a 0.2 µm syringe filter. Briefly, samples were analyzed using HPLC (Waters Separation module 2695, Waters Alliance, USA) equipped with a PAD detector (2996, Waters Alliance, USA), and C18 reverse-phase column for separation. Each sample (20 µL) was injected and eluted with 1 ml min^-1^ using a step-by-step gradient to increase the amount of acetonitrile (ACN) in water. The gradient started at 40% of ACN for 4 min, then increased to 64% in 7.5 min, reached 75% in 16.5 min, and ended at 100% in 18.5 min (100% was maintained for 3 min before being reduced to 40% for 5 min). Chromatograms were recorded at 270 nm, and the 2,4-DAPG was quantified according to standard curve with 2,4-DAPG (Cayman, Michigan, USA) (Vacheron et al., 2018).

Finally, the organic compounds were separated by gas chromatography Agilent 7890A Series Gas Chromatograph interfaced to an Agilent 5973c Network Mass Selective Detector and an Agilent 7683 Series Injector, Agilent Technologies, Santa Clara, CA, USA (GC-MS). A 5 µL CFS of the selected bacterial isolate was injected with a 1:5 split (sample; carrier gas) through 0.3% SD onto a HP-5MS column (30 m × 0.25 mm I.D., 0.25 µm film thickness, Agilent Technologies, Santa Clara, CA, USA) using He as the carrier gas at 1 mL min^-1^. The ion source was maintained at 250°C; the GC oven was programmed with a temperature gradient starting at 40°C (for 5 min) and gradually increased to 300°C (for 5 min) at 5°C min^-1^. Mass spectrometry analysis was performed in electron impact mode at an ionization potential of 70 eV. Mass spectra from m/z 40 to 800 were recorded. Identification of organic compounds was performed using Agilent Technologies Enhanced ChemStation (G1701EA ver. E.02.00.493) and The Wiley Registry of Mass Spectral Data (version 3.2, Copyright 1988–2000 by Palisade Corporation with, 8^th^ 213 Edition with Structures, Copyright 2000 by John Wiley and Sons, Inc., Hoboken, NJ, USA).

### 2.5 Genome mining

The bacterial genomic DNA was isolated using the Bacterial & Yeast Genomic DNA Purification Kit (EURx) as per the manufacturer’s protocols. The quality of the extracted DNA was measured and verified using the NanoDrop 2000 spectrophotometer (Thermo Fisher Scientific, Wilmington, USA) and gel electrophoresis in ChemiDoc XRS+ (Bio-Rad, USA). Finally, the whole-genome sequencing was outsourced to Eurofins Genomics Europe Shared Services GmbH, Germany, and was performed using the Illumina HiSeq platform.

Raw sequencing reads were quality filtered and trimmed using fastp software run with the following flags –detect_adapter_for_pe –correction (Chen et al., 2018). Reads assembly was performed using SPAdes assembler (version 3.15.5) and its quality was assessed using QUAST (version 5.2.0) (Mikheenko et al., 2018; Prjibelski et al., 2020). Comprehensive Genome Analysis service from the Bacterial and Viral Bioinformatics Resource Center (BV-BRC) information system was used to annotate the genome assembly (Olson et al., 2023). ReCOGnizer tool was used to assign COG functional categories to the identified gene sequences (Sequeira et al., 2022). Further annotation, such as metabolic pathways mapping and mining for secondary metabolite biosynthetic gene clusters, was conducted using KEGG Automatic Annotation Server and antiSMASH services, respectively (Moriya et al., 2007; Blin et al., 2021).

Additionally, the genome was inspected for presence of 145 proteins covering the following processes or reactions involved in biocontrol and plant growth promotion: ACC deamination (genes: acdS), acetoin and 2,3-butanediol synthesis (genes: *budA*, *budB*, *budC*), achromobactin biosynthesis and transport (genes: *acsA*, *acsB*, *acsC*, *acsD*, *cbrA*, *cbrB*, *cbrC*, *cbrD*), alkaline protease biosynthesis (genes: *aprA*), ammonia assimilation (genes: *gltB*, *gltD*, *gltP*, *gltS*, *gltT*), auxin biosynthesis (genes: *ipdC*), bacillibactin biosynthesis (genes: *dhbA*, *dhbB*, *dhbC*, *dhbE*, *dhbF*), biofilm formation (genes: *efp*, *flgB*, *flgC*, *flgD*, *flgE*, *flgF*, *flgG*, *flgH*, *flgI*, *hfq*, *motA*, *motB*), chitinase biosynthesis (genes: *ChiC*), elastase biosynthesis (genes: *lasA*, *lasB*), enterobactin biosynthesis (genes: *entD*, *fepA*, *fes*), exopolysaccharides biosynthesis (genes: *algA*, *algB*, *algD*, *algE*, *algG*, *algK*, *algL*, *algX*), fengycin synthesis (genes: *fenA*, *fenB*, *fenC*, *fenD*, *fenE*), GABA biosynthesis (genes: *gabD*, *gabT*), HCN biosynthesis (genes: *hcnA*, *hcnB*, *hcnC*), IAA biosynthesis (genes: *trpA*, *trpB*, *trpC*, *trpD*, *trpE*, *trpF*, *trpG*), lipase biosynthesis (genes: *lipA*, *lipB*), nitric oxide synthesis (genes: *nirK*), phenazine biosynthesis (genes: *phzA*, *phzB*, *phzC*, *phzD*, *phzE*, *phzF*, *phzG*, *phzH*, *phzM*, *phzS*), phosphate solubilization (genes: *pqqA*, *pqqB*, *pqqC*, *pqqD*, *pqqE*, *pqqF*, *pqqG*, *pstA*, *pstB*, *pstC*, *pstS*), protease IV biosynthesis (genes: *prpL*), pyochelin receptor (genes: *fptA*), pyoverdine receptor (genes: *fpvA*), pyoverdine biosynthesis (genes: *pvdA*, *pvdD*, *pvdE*, *pvdF*, *pvdG*, *pvdH*, *pvdI*, *pvdJ*, *pvdL*, *pvdM*, *pvdN*, *pvdO*, *pvdP*, *pvdQ*, *pvdS*, *pvdY*), rhamnolAAipid biosynthesis (genes: *rhlA*, *rhlB*, *rhlC*), surfactin biosynthesis (genes: *srfAA*, *srfAB*, *srfAC*, *srfAD*), urease biosynthesis (genes: *ureA*, *ureB*), vibrioferrin synthesis (genes: *pvsA*, *pvsB*, *pvsC*, *pvsD*, *pvsE*). Furthermore, the following 24 *Pseudomonas* sp. were screened for the aforementioned genes to enable a suitable comparison: *Pseudomonas aeruginosa* B18 (GCF_013395035.1), *Pseudomonas aeruginosa* FG106 (GCF_021044445.1), *Pseudomonas aeruginosa* L10 (GCF_002223805.1), *Pseudomonas aeruginosa* M18 (GCF_000226155.1), *Pseudomonas aeruginosa* PAO1 (GCF_000006765.1), *Pseudomonas chloropsis* JD37 (GCF_000761195.1), *Pseudomonas chlororaphis* HT66 (GCF_000597925.1), *Pseudomonas chlororaphis* PA23 (GCF_000698865.1), *Pseudomonas chlororaphis* subsp. aurantiaca (GCF_030908505.1), *Pseudomonas fluorescens* CHA0 (GCF_900560965.1), *Pseudomonas fluorescens* SBW25 (GCF_000009225.2), *Pseudomonas fluorescens* UM270 (GCF_000836415.1), *Pseudomonas protegens* FD6 (GCF_003363755.1), *Pseudomonas protegens* Pf-5 (GCF_000012265.1), *Pseudomonas psychrotolerans* CS51 (GCF_006384975.1), *Pseudomonas putida* BIRD-1 (GCF_000183645.1), *Pseudomonas putida* LWPZF (GCF_022354785.1), *Pseudomonas seleniipraecipitans* D1-6 (GCF_001839645.2), *Pseudomonas simiae* WCS417 (GCF_000698265.1), *Pseudomonas* sp. ANT_H12B (GCF_008369325.1), *Pseudomonas* sp. B10 (GCF_900156235.1), *Pseudomonas* sp. UW4 (GCF_000316175.1), *Pseudomonas* sp. VI4.1 (GCF_002029345.1), *Pseudomonas syringae* GR12-2 (GCF_001698815.1). Proteomes were subjected to comparison against reference protein sequences using BLASTp (Camacho et al., 2009). Only alignments meeting the following thresholds were subjected to further analysis: e-value not exceeding 1e-5, minimum sequence identity of at least 50% and sequence query coverage per HSP higher than 70%.

### 2.6 Evaluation of the antagonist effect of selected isolate against *B. cinerea* on *Solanum lycopersicum* var. *cerasiform*e postharvest

#### 2.6.1 Bioassay in controlling *B. cinerea* on cherry tomato

Biocontrol efficacy of selected isolate against *B. cinerea*-caused post-harvest gray mold rot on tomato was performed. Fruit of cherry tomato (*Solanum lycopersicum* var. *cerasiforme*) were obtained from a local organic vegetable market (Warsaw, Poland). Fruit were surface sterilized by washing with tap water, then soaked with 70% alcohol solution for 3 min, rinsed three times with sterile distilled water, and surface dried (Bu et al., 2021). As a control (CT), some of the surface-sterilized tomatoes were used. Every other tomato was pierced with a sterile toothpick to obtain a wound (5 mm wide, 3 mm deep). In T1 and T2 treatments, 10 µL of selected bacterial culture (10^11^ CFU mL^-1^) and CFS were added to each wound, respectively. After incubation for 4 h at 25°C, the wounds of tomato fruit were inoculated with mycelial disks of *B. cinerea*. Treatments T3 and T4 served as controls for T1 and T2; therefore, only inoculated with 10 µL of selected bacterial culture (10^11^ CFU mL^-1^) and CFS, respectively into the tomato wounds. T3 and T4 aimed to exclude the negative effects of bacterial cells or supernatant on tomatoes. In the T5 treatment, tomato wounds were inoculated with mycelial disks of the pathogen and phloroglucinol (10 µL, 1 g L^-1^) as an efficient antifungal agent. In addition, two more controls were performed by adding sterile distilled water (CW) or sterile distilled water (dH_2_O) and inoculated with mycelial disks of *B. cinerea* (CWB) on tomato’s wound (**Table 1**).

**Table 1:** Experimental design for evaluating the effect of selected PGPB in controlling *B. cinerea* on tomatoes.

Code	Treatment description
CT	Without puncturing
CW	Punctured + sterile dH_2_O
CWB	Punctured + sterile dH_2_O + *B. cinerea*
T1	Punctured + bacterial cell suspension + *B. cinerea*
T2	Punctured + cell-free supernatant + *B. cinerea*
T3	Punctured + bacterial cell suspension
T4	Punctured + cell-free supernatant
T5	Punctured + phloroglucinol + *B. cinerea*

All fruit were stored in a sterile transparent plastic box at 24 - 25°C. The experiment was performed in triplicate for each treatment with 5 cherry tomatoes. The diameter of the disease lesion and *B. cinerea* growth was measured at four, and seven days post–inoculation (dpi). The inhibition rate was calculated according to Xu et al. (2021).


Eq. (E) $$\text{Inhibition rate}=\frac{\text{LS}cwb-\text{LS}t}{\text{LS}cwb}\text{x }100\%$$

LS*cwb* is the average area of lesions infected by *B. cinerea* on fruit inoculated with distilled water; LS*t* is the average area of lesions infected by *B. cinerea* on fruit inoculated with bacterial culture or CFS or phloroglucinol.

#### 2.6.2 Evaluation of postharvest quality parameters of cherry tomato

Different parameters *viz.* total antioxidant, total phenolic content, ascorbic acid content, and titratable acidity were analyzed to assess the postharvest quality of cherry tomatoes. Fruit were crushed using sterilized mortar and pestle, and homogeneous mass was centrifuged at 8,228× *g* and 4°C for 10 min. The collected supernatant (fruit extract) was used for the analysis.

##### 2.6.2.1 Total antioxidants

The total antioxidants content of fruit extract was measured by the 2,2-diphenyl-1-picrylhydrazyl (DPPH) assay described by Hosseini et al. (2018). 50 μL of fruit extract was added to 250 μL of 0.025 g L^-1^ DPPH (free radical, 95%; Sigma-Aldrich, St. Louis, USA) in methanol. The mixture was thoroughly mixed before being incubated in the dark for 30 min. The OD was measured with spectrophotometer at 515 nm, with methanol as a blank.

##### 2.6.2.2 Total phenolic content

The Folin-Ciocalteu method was performed according to Vallverdú-Queralt et al. (2011). Tomato extract (200 μL) was mixed with 1800 μL distilled water. Then, 120 μL Folin reagent (Sigma-Aldrich, St. Louis, USA) and 300 μL Na_2_CO_3_ (20%) was added to the mixture. Following an hour of dark incubation, OD was measured at 760 nm. Gallic acid was used as a standard and the total phenolic content was expressed as gallic acid equivalent in g gallic acid kg^-1^ fresh weight (FW).

##### 2.6.2.3 Ascorbic acid content

The ascorbic acid concentration in fruit extract was determined according to the protocol of Hosseini et al. (2018) with slight modifications. The solution containing iodine (I) and potassium iodide (KI) (2 g KI and 1.3 g I in 1 L water) was used for titration until color of sample turned dark blue and was stable for a few seconds. The volume of I+KI solution was recorded and the concentration of ascorbic acid was calculated.

##### 2.6.2.4 Titratable acidity

Titratable activity was quantified by diluting 10 mL of tomato extract with an equal volume of distilled water. Then two drops of 1% (v/v) phenolphthalein were added as an indicator. The sample was then titrated with 0.1 N sodium hydroxide (NaOH) solution until color changed to light pink and persisted for at least 15 sec. Titratable activity was expressed and calculated as a percentage of citric acid according to Zainal et al. (2019).

### 2.7 Statistical analysis

Data were statistically analyzed using IBM Statistic SPSS 22 computer software. One-way analysis of variance was performed to evaluate the effect inoculation of selected isolate in inhibiting the growth of *B. cinerea* BC21 and improving postharvest quality of cherry tomatoes. Differences between means at P< 0.05 were evaluated by Duncan’s multiple range tests.

## 3 Results

### 3.1 Antagonistic activity of bacterial isolates against *B. cinerea* BC21

**Figure 1:**
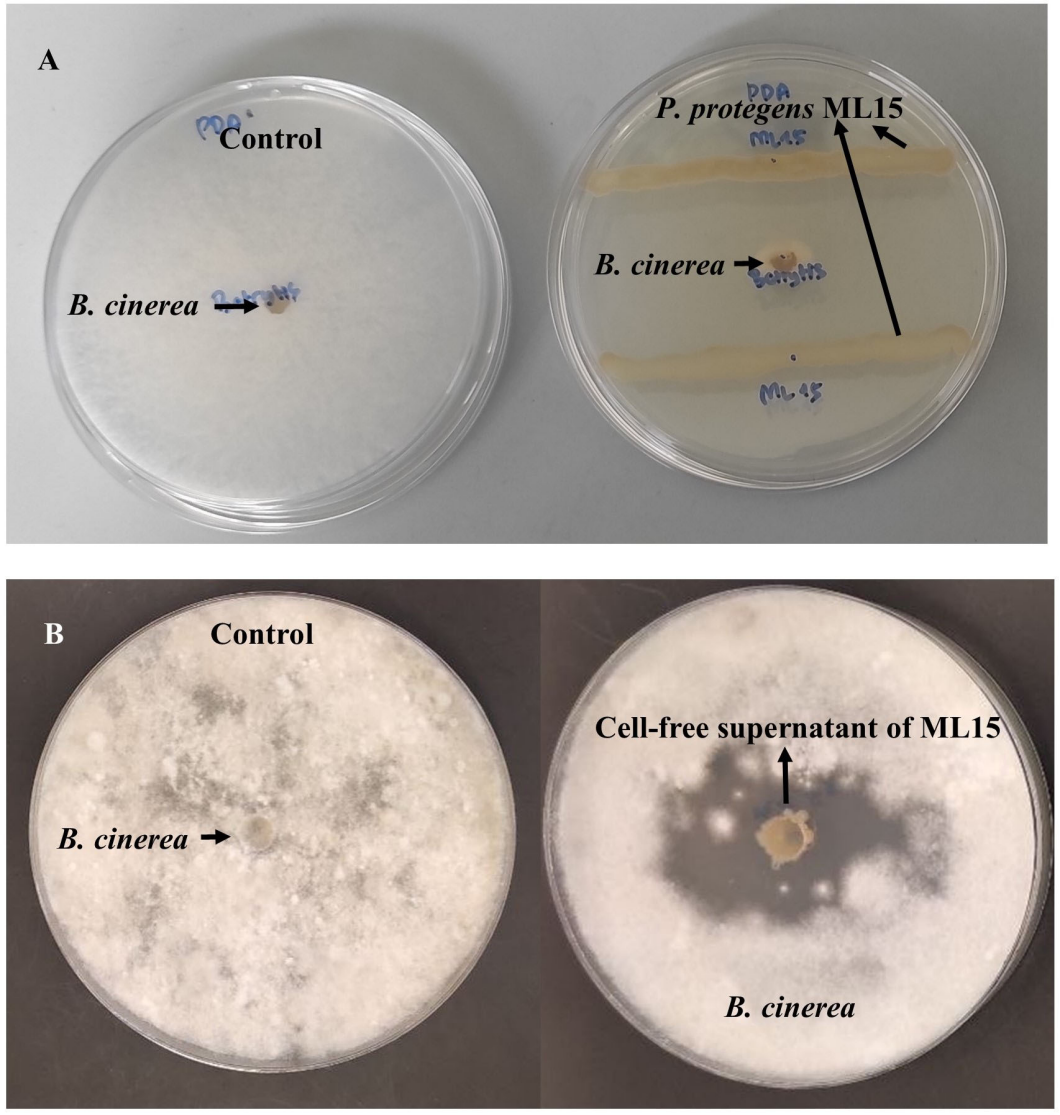
Antagonistic activity of *P. protegens* ML15 against *B. cinerea* BC21. [The antagonistic activity of *P. protegens* ML15 against *B. cinerea* BC21 was evaluated through two different methodologies, **(A)**: dual culture growth inhibition method; **(B)**: agar well diffusion method using cell-free supernatant].

Amongst eight tested isolates, only *P. protegens* ML15 demonstrated antagonistic activity against *B. cinerea* BC21 using dual culture growth inhibition method with inhibition rate achieved 77% (**Figure 1**). Moreover, the antagonistic activity of CFS derived from *P. protegens* ML15 was detected against *B. cinerea* BC21 by the agar well diffusion method. The maximum zone of inhibition, measuring a remarkable 24.5 mm, was observed when utilizing the three-day-old culture filtrate supernatant (CFS) of *P. protegens* strain ML15, as depicted in **Supplementary Figure 1**. As a result of this significant finding, we decided to consistently employ three-day-old CFS in all subsequent investigations. Application of CFS of *B. ambifaria* AF8II10, *B. cereus* AF8II13, *B. thuringiensis* EDC1, *K. aerogenes* AF3II1, *P. putida* AF1I1, *S. marcescens* AF8I1, and *S. plymuthica* EEPC5 did not reduce the mycelium growth of *B. cinerea* BC21.

### 3.2 Biocontrol activity of *P. protegens* ML15

*P. protegens* ML15 was chosen as the selected isolate because had ability to effectively inhibit the growth of *B. cinerea* BC21. Subsequently, an investigation was conducted to explore the various biocontrol mechanisms employed by this selected isolate against phytopathogenic fungi. The production of siderophores by *P. protegens* ML15 was evidenced by the development of an orange halo zone with an index of 1.5 after five days of incubation around the colony on the CAS agar plate (**Supplementary Figure 2**). Moreover, HCN production was confirmed in a simple plate test by development of a brown color in filter paper soaked with picric acid. The formation of a halo zone in Tween 20-containing medium indicated lipolytic activity by the lipase production. No activity was detected indicating the presence of extracellular chitinolytic, cellulolytic, and proteolytic enzymes. Furthermore, our study revealed that *P. protegens* ML15 was capable of producing EPS at a concentration of 570 mg L^-1^ and ammonia at 14.61 mg L^-1^ after five days of culture incubation. The biosurfactant produced by strain ML15 exhibited emulsification activity, with an emulsification index of 48.8%. Moreover, the growth of *B. cinerea* BC21 mycelia was significantly hampered by *P. protegens* ML15, primarily due to the production of VOCs. After seven days of incubation, the inhibition rate reached 76.8 ± 2.4% compared to the control. These findings highlight the diverse and effective biocontrol mechanisms employed by *P. protegens* ML15 to combat phytopathogenic fungi, making it a promising candidate for further research.

### 3.3 Identification and estimation of antifungal metabolites of *P. protegens* ML15

The cell-free supernatant (CFS) obtained from a three-day-old culture of *P. protegens* ML15 demonstrated the most pronounced antifungal activity. Consequently, we selected a three-day incubation period as the reference for the identification of secondary metabolites produced by *P. protegens* ML15. The secondary metabolites from *P. protegens* ML15 culture were first investigated by the TLC method. By observing the appearance of compounds from the cell-free extract of *P.protegens* ML15 which were compared with standard on TLC plate, indicated the compounds could be phloroglucinol (**Supplementary Figure 3**). Furthermore, we assessed that 2,4-DAPG from *P. protegens* ML15 reached 9.4 mg L^-1^ after three days of incubation by the HPLC method, confirming the production of phloroglucinol.

Subsequently, the identification of bioactive organic compounds secreted by *P. protegens* ML15 was carried out using GC-MS. The analysis of the ethyl acetate extract revealed the presence of 29 bioactive metabolites, with a similarity of over 90% to the library data within The Wiley Registry of Mass Spectral Data and Agilent Technologies Enhanced ChemStation (G1701EA ver. E.02.00.493) (**Supplementary Figure 4**). The primary chemical compounds with area fraction greater than 1% were identified as ethylbenzene, m-xylene, o-xylene, 1-isopropyl-2-methylbenzene, 1-tetradecene, tetradecane, 2,6-di-tert-butyl-p-benzoquinone, cyclododecane, 2,4-di-t-butylphenol, 2-methyl-1-hexadecanol, 1-tetradecanol (**Table 2**). The presence of these bioactive organic compounds may considerably contribute to the biocontrol abilities of *P. protegens* ML15.

**Table 2:** GC-MS profile of bioactive compounds produced by *P. protegens* ML15.

Bioactive compound	Chemical formula	Retention time (min)	Area (%)
Methylbenzene	C_7_H_8_	7.331	0.85
1,2-Dimethylcyclohexane	C_8_H_16_	7.959	0.03
Ethylbenzene	C_8_H_10_	10.035	1.54
m-Xylene	C_6_H_4_(CH_3_)_2_	10.365	3.49
3-Methylbutyl acetate	C_7_H_14_O_2_	10.844	0.39
o-Xylene	C_6_H_4_(CH_3_)_2_	11.284	1.12
Nonane	C_9_H_20_	11.659	0.10
Tricyclo [2.2.1.0(2,6)]heptane	C_7_H_10_	12.513	0.13
(1-methylethyl)benzene	C_11_H_16_	12.746	0.04
2,2-Dimethyl-3- methylidenebicyclo[2.2.1]heptane	C_10_H_16_	13.826	0.25
1-Methyl-2-ethylbenzene	C_9_H_12_	14.764	0.10
1,3,5-Trimethylbenzene	C_9_H_12_	15.049	0.09
O-Ethyltoluene	C_9_H_12_	15.637	0.04
Decane	C_10_H_22_	17.009	0.08
3,7,7-Trimethylbicyclo[4.1.0]hept-3-ene	C_10_H_16_	17.371	0.14
1-Isopropyl-2-methylbenzene	C_10_H_14_	18.328	1.90
Pentylcyclopentane	C_10_H_20_	18.988	0.19
5-Methyl-5-undecene	C_12_H_24_	28.704	0.17
1-Decene	C_10_H_20_	29.952	0.67
Dodecane	C_12_H_26_	30.528	0.14
(5e)-5-Dodecene	C_12_H_24_	35.535	0.11
Cyclotetradecane	C_14_H_28_	41.861	0.50
1-Tetradecene	C_14_H_28_	43.420	2.22
Tetradecane	C_14_H_30_	43.685	1.49
2, 6-Di-tert-butyl-p-benzoquinone	C_14_H_20_O_2_	45.373	2.28
Cyclododecane	C_12_H_24_	46.253	7.10
2,4-Di-tert-butylphenol	C_14_H_22_O	47.308	5.07
2-Methyl-1-hexadecanol	C_17_H_36_O	48.168	7.22
1-Tetradecanol	C_14_H_30_O	50.328	4.79

### 3.4 Genome mining: comparative analysis of genes, metabolic pathways mapping, and prediction of secondary metabolite clusters involved in biocontrol traits

The whole genome sequence and assembly of *P. protegens* strain ML15 was submitted at the NCBI GenBank under BioProject reference no: PRJNA943471. Genomic DNA sequencing yielded 7,313,913 paired reads (2,208,801,726 total base pairs). Initial genome assembly consisted of 131 contigs, with mean coverage of 308 and 7,003,685 bp total assembly length. Contigs with the coverage lower than 10 and shorter than 500 bp were discarded from the assembly. The final genomic assembly consisted of 24 contigs (L50 = 2; N50 = 1,127,941; 6,972,288 bp total assembly length). The genome had 6,358 protein coding sequences (CDS), 62 transfer RNA (tRNA) genes, and 6 ribosomal RNA (rRNA) genes.

**Figure 2:**
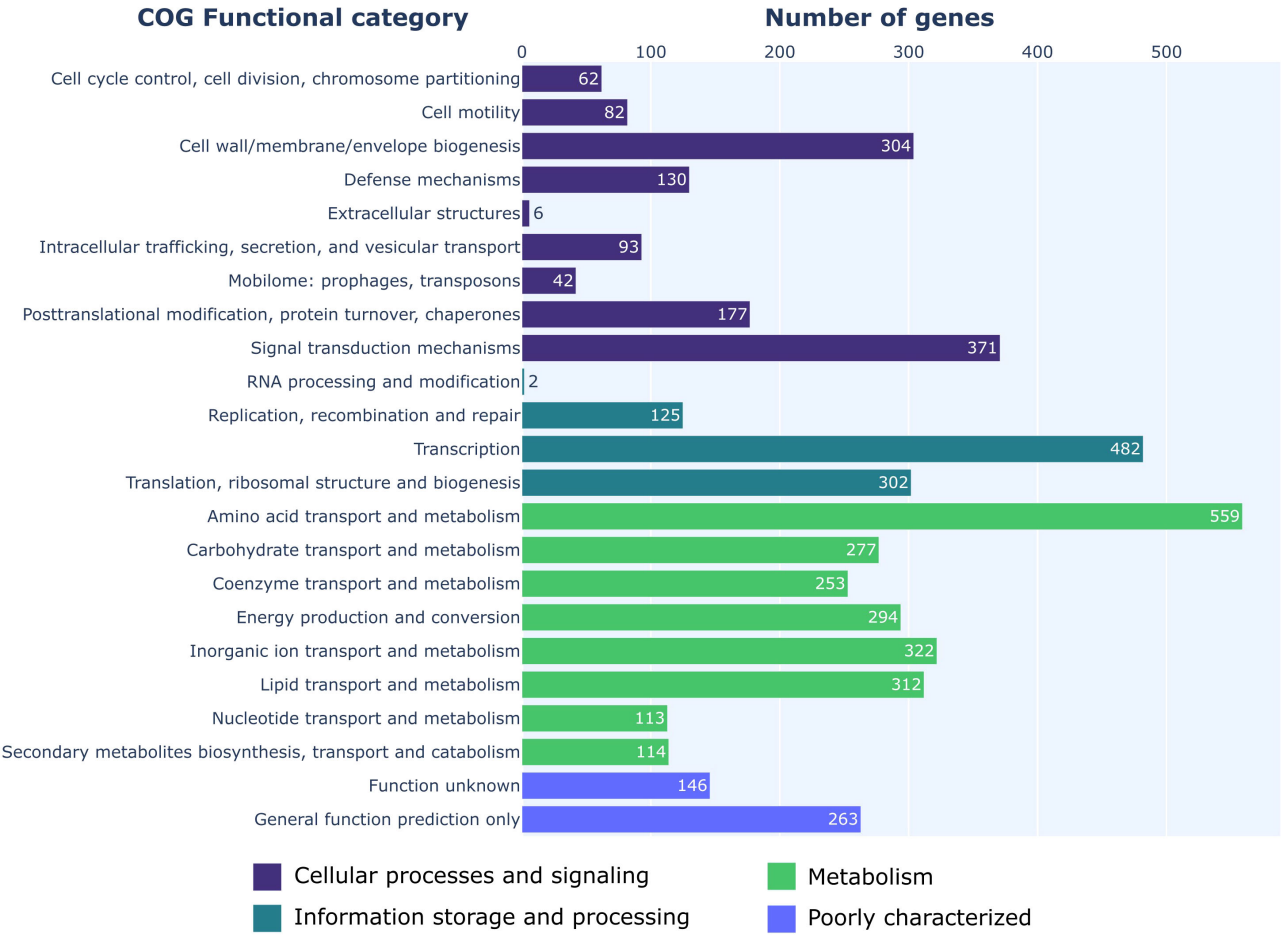
COG categories overview of *P. protegens* ML15.

**Figure 3:**
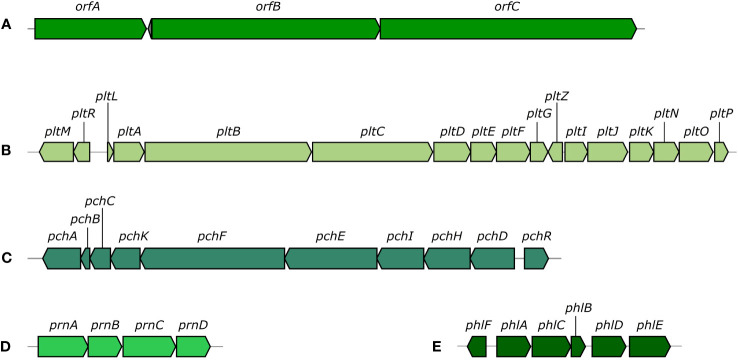
Biosynthetic gene clusters discovered using AntiSMASH. [**(A)**: orfamide A/orfamide C cluster, **(B)** pyoluteorin cluster, **(C)** enantio-pyochelin cluster, **(D)** pyrrolnitrin cluster, **(E)** 2,4-diacetylphloroglucinol cluster].

COG functional categories were successfully assigned to 4,831 genes (**Figure 2**). Of the available 26 COG functional categories, 23 unique categories were detected among the analyzed genes. Interrogation of the KEGG metabolic maps confirmed the strain’s ability to synthesize HCN and EPS matrix. Further investigation also highlighted the strain’s potential to synthesize a RTX-type toxin. AntiSMASH analysis was used to detect biosynthetic gene clusters (BGCs) that could be responsible for the production of antifungal agents. The analysis revealed 18 regions with potential for secondary metabolites biosynthesis. Out of those 18 regions, five showed near 100% similarity to known BGCs (**Figure 3**). Two of those clusters produced non-ribosomal peptides, orfamide A/C and enantio-pyochelin. Two produced polyketides, pyoluteorin and 2,4-DAPG, and one was responsible for synthesis of pyrrolnitrin (**Supplementary Table 1**).

**Figure 4:**
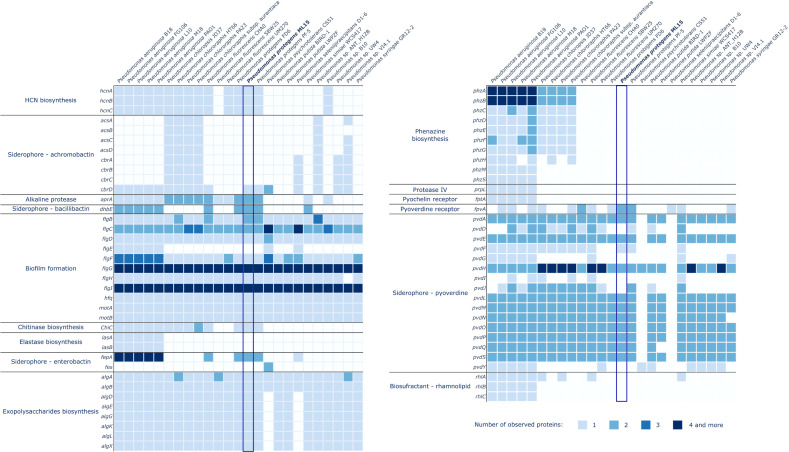
Distribution of genes involved in biocontrol in the genomes of *P. protegens* ML15 and 24 other, closely related *Pseudomonas* strains.

**Figure 5:**
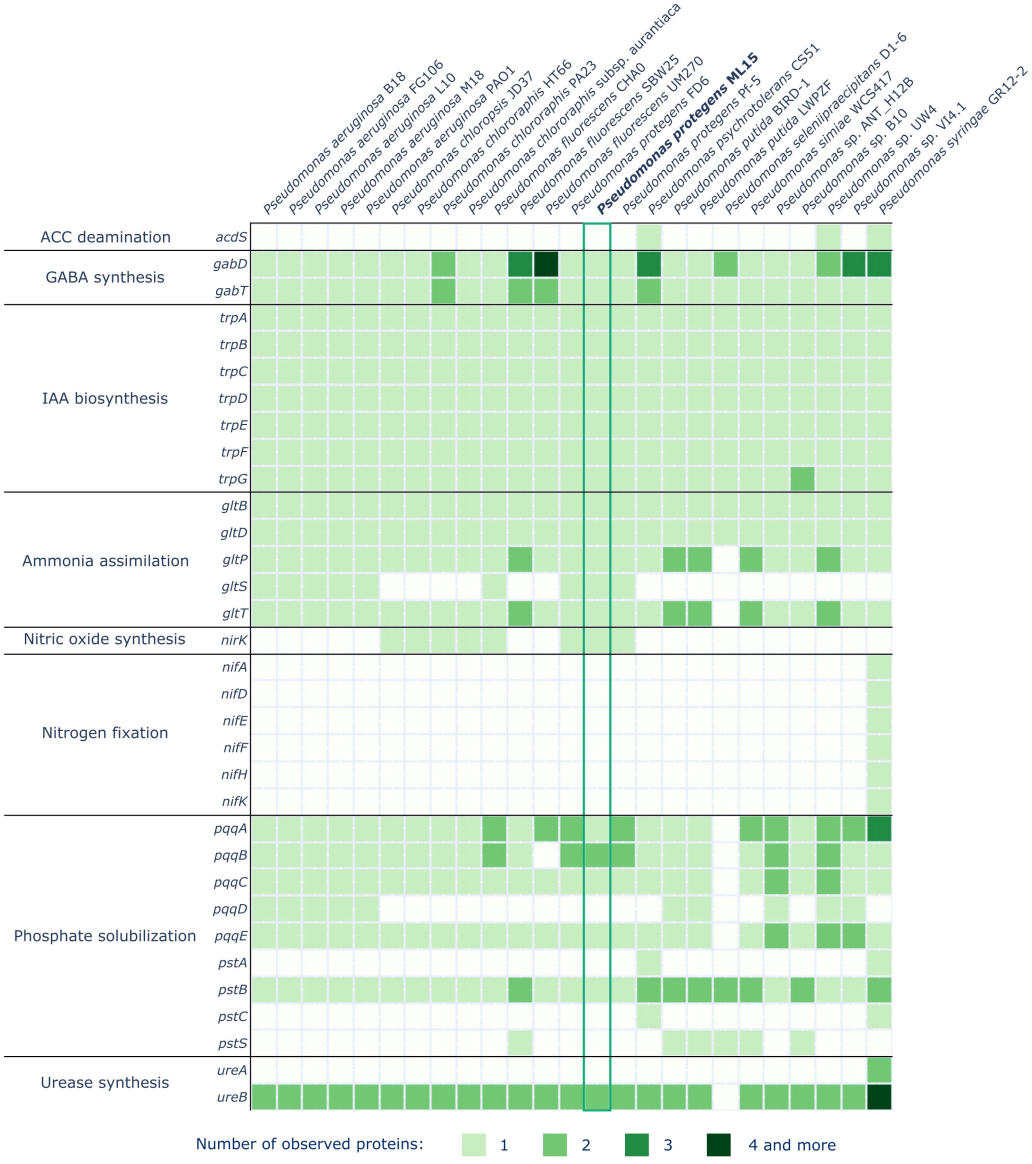
Distribution of genes involved in plant growth promotion in the genomes of *P. protegens* ML15 and 24 other, closely related *Pseudomonas* strains.

A comprehensive analysis of the *P. protegens* ML15 genome has revealed various genes encoding enzymes responsible for the production of biocontrol and plant growth promoting agents. Among the 145 investigated proteins, 59 were identified within the genome of *P. protegens* ML15, as illustrated in **Figures 4** and **5**. The investigation of enzymes associated with siderophore synthesis, distinct from those identified by the AntiSMASH program, has unveiled a nearly complete set of genes responsible for the production of pyoverdine. It is however important to note that while only 5 out of the 16 relevant genes were absent, the missing ones, and especially *pvdD*, *pvdI*, and *pvdJ* are encoding core biosynthetic enzymes. Consequently, it is highly probable that this strain lacks the capacity for pyoverdine production, at least using enzymes analogous to established references. Significantly, the genome does encompass the *fpvA* gene, encoding the pyoverdine receptor, a crucial element in pyoverdine biosynthesis (Shen et al., 2002). Furthermore, additional investigations for genes associated with siderophore biosynthesis did not uncover any novel, intact siderophore biosynthetic clusters. Nevertheless, the genome appears to contain the *dhbE* and the *fepA* genes, with the former being recognized for its involvement in bacillibactin biosynthesis and the latter in the uptake of enterobactin-bound iron.

In the context of genes related to biocontrol, the analysis has demonstrated the presence of nearly all the genes required for biofilm formation. However, the genome lacks the *flgE* gene, which encodes the flagellar hook protein, an essential component in the assembly of the flagellum in *Salmonella enterica*. Therefore, it is not clear whether the strain can synthesize a fully functional flagellum (Bonifield et al., 2000). Additionally, the screening also confirmed the strain’s ability to synthesize HCN (*hcnA*, *hcnB*, *hcnC*) and EPS matrix, which is most probably rich in alginate, as the analysis has identified the presence of the following genes: *algA*, *algB*, *algD*, *algE*, *algG*, *algK*, *algL*, and *algX*. The genome also encodes two alkaline proteases (both homologous to the *aprA* gene) and a chitinase (*ChiC*).

Within the context of plant growth-promoting agents, the screening revealed the strain’s capability to synthesize gamma-aminobutyric acid (GABA) and indole-3-acetic acid (IAA). Furthermore, the strain’s genome encodes a nitrate reductase (*nirK*), one of the urease subunits (*ureB*) and demonstrates the ability to assimilate ammonia through the utilization of enzymes encoded by the *gltBDSPT* operon. The strain also harbors several genes from two operons, *pqq* and *pst*, which are instrumental in phosphate solubilization and uptake. Sequences of the genes identified in the *P. protegens* ML15 genome are included in **Supplementary Data File 1**.

### 3.5 Bioassay of *P. protegens* ML15 in controlling *B. cinerea* on *Solanum lycopersicum* var. *cerasiforme* postharvest

**Figure 6:**
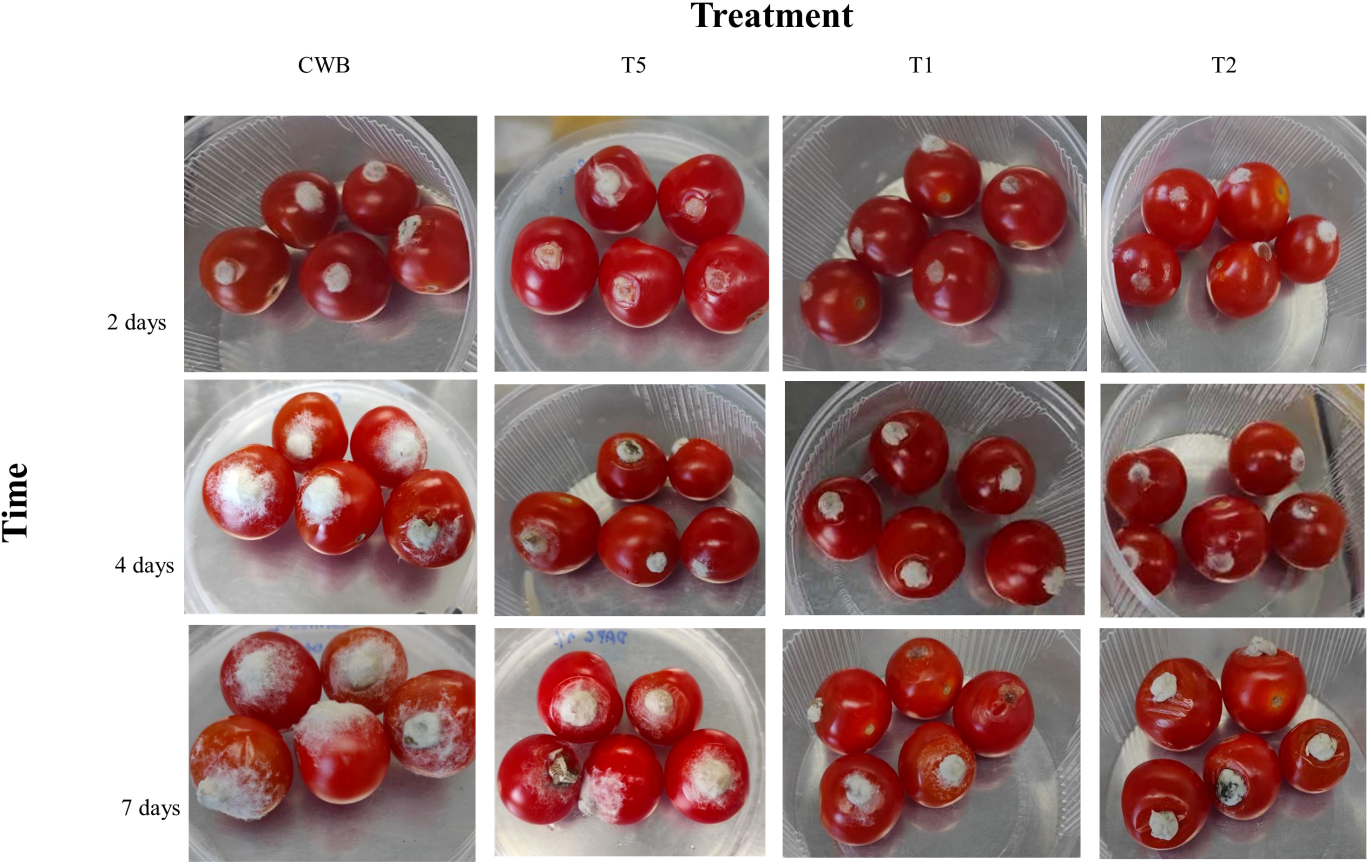
Tomatoes infected by *B*. *cinerea* after two-, four-, and seven-days post-inoculation with different treatments.

All tomatoes from treatments CWB, T1, T2, and T5 developed lesions around the inoculation site following *B. cinerea* inoculation. However, we did not examine the disease lesion and fungi growth on treatments CT, CW, T3, and T4 as they were not infected with *B. cinerea* BC21. Our study showed that bacterial treatments either T1 and T2 substantially reduced fungal growth (53.0 ± 0.63%) and disease lesion (52.8 ± 1.5%) on tomatoes compared to CWB treatments at four days post-*B*. *cinerea* inoculation. Importantly, there were no significant differences (P>0.05) in disease lesion size and growth of fungi diameter between treatments T1 and T2 (**Figure 6**; **Table 3**). According to these results, T1 and T2 treatments significantly inhibited the growth of *B. cinerea* and mitigated disease development in cherry tomatoes.

**Table 3:** Disease lesion, fungi growth, and inhibition rate of tomatoes infected by *B. cinerea*.

Treatment	Disease lesion (mm)		*B. cinerea* growth (mm)		Inhibition rate (%)	
	4 dpi	7 dpi	4 dpi	7 dpi	4 dpi	7 dpi
CWB	15.7 ± 1.5 ^a^	19.3 ± 1.0 ^a^	14.6 ± 1.8 ^a^	18.1 ± 1.2 ^a^	0	0
T5	9.1 ± 1.3 ^b^	18.0 ± 1.6 ^b^	8.5 ± 1.1 ^b^	17.7 ± 1.3 ^a^	42.1 ± 7.3 ^a^	7.6 ± 1.6 ^a^
T1	7.2 ± 0.9 ^c^	16.4 ± 1.1 ^c^	6.9 ± 0.9 ^c^	10.1 ± 1.0 ^b^	53.8 ± 7.5 ^b^	15.0 ± 3.2 ^b^
T2	7.6 ± 0.8 ^c^	16.5 ± 1.1 ^c^	7.1 ± 0.9 ^c^	10.4 ± 0.6 ^b^	51.7 ± 5.7 ^b^	14.1 ± 3.4 ^b^

Each data represents the mean ± SD of three replicates. Values with the same superscript letters are not significantly different at the test level of 5% based on Duncan’s t-test.

The application of either the bacterial culture or bacterial supernatant (T1 or T2) containing the natural secondary metabolites significantly inhibited spore germination and growth of the pathogenic fungus, thus significantly reducing the disease lesion compared to the control. Notably, the inhibitory impact of *P. protegens* ML15 was shown to be superior to that of phloroglucinol application in terms of disease lesion size and fungal development. These findings illustrated *P. protegens* ML15’s robust antifungal activity and emphasize its potential as a powerful biocontrol agent against phytopathogenic fungi, surpassing the performance of phloroglucinol, a known antifungal compound.

### 3.6 Effects of *P. protegens* ML15 on postharvest quality parameters of cherry tomatoes

The effect of biocontrol agents from *P. protegens* ML15 on the quality of tomato fruit infected by *B. cinerea* was evaluated. Treatments involving *B. cinerea* infection consistently revealed lower antioxidant activity in contrast to treatments without *B. cinerea*. However, the inoculation of bacterial culture-T1 (77.0 ± 0.76%) or CFS-T2 (79.8 ± 1.14%) significantly improved antioxidant activity compared to treatment CWB (69.9 ± 0.18%). Among the treatments, the most decrease level in antioxidant activity was observed in the CWB treatment (15.8%), followed by T1 (7.2%), T2 (3.8%), T3 (3.6%), T4 (2.4%), T5 (1.7%) and CW (1.5%) in comparison to CT treatment (**Table 4**).

**Table 4:** Postharvest quality parameters of tomatoes after seven dpi of *P. protegens* ML15 with or without being infected by *B. cinerea*.

Treatment	Antioxidant activity	Total phenolic content	Citric acid content	Ascorbic acid content
	(%)	(g kg^-1^)	(%)	(g kg^-1^)
T1	76.96 ± 0.76^b^	0.85 ± 0.03^b^	0.36 ± 0.03^b^	0.047 ± 0.007^b^
T2	79.76 ± 1.14^bc^	0.85 ± 0.01^b^	0.38 ± 0.01^b^	0.043 ± 0.003^b^
T3	79.92 ± 1.30^bc^	0.89 ± 0.03^b^	0.25 ± 0.01^a^	0.058 ± 0.002^c^
T4	80.93 ± 2.55^c^	0.86 ± 0.02^b^	0.25 ± 0.02^a^	0.046 ± 0.004^b^
T5	81.55 ± 0.77^c^	0.98 ± 0.01^c^	0.48 ± 0.08^c^	0.062 ± 0.004^c^
CWB	69.87 ± 0.18^a^	0.76 ± 0.05^a^	0.36 ± 0.02^b^	0.033 ± 0.004^a^
CW	81.69 ± 0.39^c^	0.98 ± 0.03^c^	0.38 ± 0.01^b^	0.062 ± 0.005^c^
CT	82.95 ± 1.02^c^	1.04 ± 0.02^c^	0.52 ± 0.06^c^	0.071 ± 0.004^d^

Each data represents the mean ± SD of three replicates. Values with the same superscript letters are not significantly different at the test level of 5% based on Duncan’s t-test.

The content of total phenolic compounds in tomatoes was determined by using Folin-Ciocalteu reagent, and expressed in terms of gallic acid equivalent. Across all treatments, the total phenolic content of tomatoes in all treatments were statistically significantly reduced compared to control CT. The lowest total phenolic content, which was 0.76 ± 0.05 g kg^-1^ was exhibited by CWB. In general, T1 and T2 (0.85 ± 0.03 g kg^-1^) showed slightly lower total phenolic content compared to T3 and T4 (0.86 – 0.89 ± 0.03 g kg^-1^). However, no statistically significant differences were observed among these treatments. On the other hand, both CW and T5 contained higher content of total phenolic (both 0.98 ± 0.03 g kg^-1^) than T1 and T2, but the same time lower than CT (1.04 ± 0.02 g kg^-1^).

Similar to other bioactive compounds, a decrease in ascorbic acid was observed in all treatments compared to the CT treatment. The most significant decrease was observed in CWB by 53% from 0.071 ± 0.004 g kg^-1^ to 0.033 ± 0.004 g kg^-1^. Otherwise, the lowest reduction rate of ascorbic acid occurred in CW and T5 was about 14.9% with a ascorbic acid content 0.062 ± 0.004 g kg^-1^, and followed by T3, with a ascorbic acid content around 0.058 ± 0.002 g kg^-1^. The same trend also observed in tomatoes infected with fungi and inoculated with bacteria, where T1 caused a smaller reduction in ascorbic acid than T2.

Titratable acid content of each tomato juice sample was quantified as % citric acid. Treatments with *B. cinerea* infection generally had a higher rate of citric acid than those without *B. cinerea* infection. However, there was no significant difference in citric acid content between CW (0.38 ± 0.01%), T1 and T2 (0.36 ± 0.03% and 0.38 ± 0.01%, respectively) as well as in CWB (0.36 ± 0.02%) treatments. The lowest percentage of citric acid was observed in treatments T3 and T4, with both measuring 0.25 ± 0.02%. In comparison, CT treatment had 0.52 ± 0.06% of citric acid. These findings suggested that the application of *P. protegens* ML15 and its bioactive substances may have an impact on tomato quality and nutritional value by altering the levels of important phytochemicals such as antioxidant activity, total phenolic content, and ascorbic acid. It also highlights *P. protegens* ML15’s potential as a biocontrol agent that can improve tomato fruit quality in the presence of pathogenic stress.

## 4 Discussion

Postharvest diseases significantly reduce the shelf-life of harvested fruits worldwide. Until now the solutions were synthetic fungicides and food preservatives, however, recently it was shown that they can be replaced with biological products based on the beneficial bacteria (Lastochkina et al., 2019).

In this study eight bacterial isolates were investigated for their antagonistic activity potential toward phytopathogenic fungi *B*. *cinerea* BC21. Either culture or CFS of *P. protegens* ML15 exhibited antagonistic effect toward *B. cinerea* BC21. Whereas other tested isolates didn’t showed antagonistic activity against *B. cinerea* BC21, therefore *P. protegens* ML15 was selected for further analysis. Several studies reported that members of *Pseudomonas*, including *P. protegens, P. fluorescens, P. putida, P. aeruginosa, P. libanensis, P. antarctica, P. rhodesiae* significantly suppressed mycelia growth of several phytopathogenic fungi, including *B. cinerea*, which is consistent with our research (Müller et al., 2018; Andreolli et al., 2019; Roca-Couso et al., 2021).

Furthermore, several mechanisms of action of the *P. protegens* ML15, potentially significant in its activity against phytopathogens, have been characterized in the current study. The selected strain showed the ability to produce siderophores, HCN, lipase, EPS, ammonia, and biosurfactant, which are well known as substances with strong antagonistic properties towards the growth of fungi. Microorganisms excrete the siderophores in response to low concentration of bioavailable iron. It can be used as a strategy to compete and inhibit the growth of pathogenic microorganisms by chelating iron from the environment (Sayyed et al., 2019). Specifically, *P. protegens* produces pyoverdine as iron-scavenging compound (Li et al., 2022). HCN is a toxic molecule to all aerobic organisms, including fungi since it can block cytochrome c oxidase and other metalloenzymes (Azeem et al., 2022). Multiple *Pseudomonas* species, such as *P. putida* R32, *P. chlororaphis* R47, and *P. chlororaphis* PA23 have been reported to be able to produce HCN and that contributed to the reduction of mycelia growth of pathogenic fungi (Nandi et al., 2017; Anand et al., 2020). Moreover, *P. protogens* ML15 displayed lipolytic action, which indicate probable lipase production, which is a useful feature in mycelium growth inhibition. The lipase action seems to deliver antagonistic activity against fungi by its cell wall degrading ability (Chalotra et al., 2019). Furthermore, *P. protegens* ML15 was positively checked for EPS synthesis. A study conducted by Tewari and Arora (2014) showed that EPS-producing *P. aeruginosa* PF23EPS significantly inhibited *Macrophomina phaseolina*. This feature, in addition to protecting plants against abiotic and biotic stress, has been assessed as important in the fight against fungal phytopathogens. As it is indicated that the production of ammonia contributes to the reduction of fungal growth, this feature was also checked for the analyzed strain. Prigigallo et al. (2021) reported that ammonia production from *P. protegens* CHA0’s played a major role in inhibiting *Hetereobasidion abietinum* growth both directly or through alkalization of the medium. Biosurfactant production confirmed for *P. protegens* ML15 suggested a probable role in the inhibition of fungi by disrupting fungal cell membranes as Goswami et al. (2015) suggested in their research.

In this research, we confirmed that, cell-free extract of *P. protegens* ML15 contained phloroglucinol. Phloroglucinol derivatives are a large class of secondary metabolites widely distributed in plants, brown algae, and some microorganisms (Biessy and Filion, 2021). One of phloroglucinol derivatives from some *Pseudomonas* strains that predominantly belong to the *P. protegens* and *P. corrugata* is DAPG (Höfte, 2021). This compound is well known for its ability to control numerous soil-borne plant diseases, including take-all of wheat, tobacco black root rot and sugar beet damping-off (Biessy and Filion, 2021; Trejo-Raya et al., 2021). *Pseudomonas protegens* DSMZ13134 was found to produce 2,4-DAPG at a concentration not exceeding 0.5 mg L^-1^ under various culture conditions, including variations in culture medium, temperature, and incubation duration (Pellicciaro et al., 2021). In a study by Patel and Archana (2017), they conducted a comparison of 2,4-DAPG production between the wild-type *P. protegens* Pf-5*, P. protegens* G22, and a recombinant *Pseudomonas* strain. The production of 2,4-DAPG from a recombinant *Pseudomonas* strain reached 12 – 14 mg L^-1^ at 96 h. This production level was approximately tenfold greater than that observed in the wild-type strains. However, in our study *P. protegens* ML15 produced 9.4 mg L^-1^ of 2,4 DAPG at 72 h. While this production level was slightly lower than that of the recombinant strain in the previous study, it still signifies a substantial capacity for 2,4-DAPG production.

Results of double plate experiment revealed that the VOCs from *P. protegens* ML15 vastly inhibited the growth of *B. cinerea*. According to Yue et al. (2023) VOCs obtained from *P. fluorescens* ZX were able to disrupt the integrity of *B. cinerea*’s cell membrane. In line with other studies, volatile compounds produced by *Pseudomonas* strain significantly reduced the germination of spores, mycelia growth, and can cause serious morphological changes of pathogenic fungi (Wang et al., 2021; Ni et al., 2022). Moreover, bacterial VOCs are categorized into several chemical classes, including aldehydes, alcohols, esters, alkanes, alkenes, butylphenol, and sulfur compounds are key inducers for stimulating the ISR responses against pathogen infection (Zhu et al., 2022). Induced resistance emerges as a novel approach for postharvest disease management, involving the initiation of immune responses in fruits and vegetables through the application of exogenous physical, chemical, and biological elicitors (Wang and Bi, 2021).

Furthermore, other antifungal compounds from the cell-free extract of *P. protegens* ML15 were identified using GC-MS. Several identified bioactive compounds including 2,6-di-tert-butylbenzoic-1,4-quinone; phenol, 2,4-bis(1,1-dimethylethyl)-; tetradecane; hexadecane; 2,4-di-t-butylphenol; and pentadecane have been identified as having potential antibacterial, antifungal, and antioxidant properties (Dharni et al., 2014; Devi et al., 2021). As a result, the potential bioactive substances derived from *P. protegens* ML15 could play a role in critical biological processes such as, acting as elicitors, mediating intercellular communication, facilitating environmental adaptation, and strengthening defense mechanisms against pathogen microorganisms (Del Rosario Cappellari et al., 2020; Biessy and Filion, 2021).

The genome was screened for genes, subsystems and metabolic pathways that could explain the source of the *P. protegens* ML15 antifungal activity. The KEGG metabolic maps confirmed that *P. protegens* ML15 had ability to synthesize HCN and EPS matrix. HCN can be synthesized from glycine as well as from serine, using either hydrogen cyanide synthase HcnA in case of glycine, or by initial transforming serine to glycine using glycine hydroxymethyl transferase. Biosynthesis of the EPS matrix, is most probably driven by three proteins: EpsA, a polysaccharide biosynthesis/export protein, EpsC an UDP-N-acetylglucosamine 2-epimerase and EpsP protein which is a low molecular weight protein-tyrosine phosphatase. Furthermore, AntiSMASH detected BGCs that could be responsible for the production of antifungal agents, including orfamide A/C, enantio-pyochelin, pyoluteorin, 2,4-DAPG, and pyrrolnitrin. Orfamids are a class of cyclic lipodepsipeptides produced by the members of *Pseudomonas* genus that exhibit biosurfactant and anti-microbial activities (Geudens and Martins, 2018). Enantio-pyochelin is a siderophore with high affinity for Fe^3+^ ions. In a study by Loper et al. (2007) on anti-fungal activity of *P. protegens* Pf-5, enantio-pyochelin has been highlighted as a major contributor to the strains antifungal activity. Pyoluteorin, 2,4-DAPG and pyrrolnitrin are natural antibiotics with strong antifungal activity. It has been recently shown that pyoluteorin and 2,4-DAPG play a crucial role in the *P. protegens* Pf-5 biocontrol against *B. cinerea*. Moreover, pyrrolnitrin is known for its broad-spectrum antimicrobial activity and low phototoxicity which has made it an interesting target for biotechnological investigations (Pawar et al., 2019; Balthazar et al., 2022b). Cluster Blast analysis showed that all five of the highly similar BGCs are conserved within the *Pseudomonas* genus, and most of them are specific to the *P. protegens* species.

Due to the suppression of *P. protegens* ML15 on the growth of *B. cinerea*. We artificially wounded and inoculated the tomato fruit with the pathogen fungal disc to check the ability of the potent *Pseudomonas* isolate to protect ripe tomato. Tomato fruit has been widely used as a model system for studying the development and ripening of fleshy fruit organs (Momo et al., 2022). Moreover, the interaction between tomato fruit and *B. cinerea* serves as a model for climacteric fruit-necrotroph interactions (Blanco-Ulate et al., 2015). Ripening tomato fruit is particularly susceptible to *B. cinerea*, while immature fruit is resistant to gray mold (Blanco-Ulate et al., 2015; Liu et al., 2022). *Botrytis* infection typically begins with penetration of the host surface and mostly infected tissues are characterized by a mass of gray conidia. Next step, killing of host tissue/primary lesion formation, lesion expansion, and sporulation (Abbey et al., 2019; Poveda et al., 2020). Our research found that bacteria inoculation (cell suspension or cell-free supernatant) significantly inhibited the spore germination (no gray spore formation), and the mycelium growth, which consequently reduced the disease lesion compared to the control. Furthermore, as stated previously, our selected bacteria were able to produce a variety of antifungal secondary metabolites which probably led to reducing gray mold rot infection. Moreover, those compounds potentially acted as inducers, triggering the activation of ISR responses against pathogens within tomatoes. Xu et al. (2021) reported that inoculating tomatoes with CFS of *Bacillus velezensis* QSE21 provided a 67.3% effective defense against *B. cinerea* mycelium infection after three days of incubation. In another study conducted by Gao et al. (2018) inoculation of *Pseudomonas* QBA5 supernatant reduced disease lesion by 81.2% in tomatoes infected by *B. cinera* spores suspension at three days of incubation. Our study showed that after four days of incubation, *P. protegens* ML15 had control effect of *B. cinerea* mycelium infection on tomatoes by 53.8% (**Table 5**). It is widely acknowledged that the mycelial form of fungi has a tendency to spread rapidly in comparison to spores. In our study, we deliberately utilized mycelium to demonstrate the enhanced efficacy of the selected strain. Our findings clearly indicated that tomatoes infected with fungal mycelium experienced more pronounced negative effects compared to the spore forms. Furthermore, the duration of the infection period can significantly influence the development of the fungus, as fungi thrive and grow over time.

**Table 5:** Comparative data for the bio-efficacy potential of *P. protegens* ML15 and other beneficial bacteria against *B. cinerea* on tomato.

*B. cinerea* inoculation	Bacterial inoculation	Bacterial strain	Incubation conditions	Inhibition rate (%)	Parameter	Reference
Spores suspension	Bacterial culture	*Bacillus tequilensis* XK29	3 d, 25°C	66.7	Disease lesion	Guo et al. (2023)
Mycelial disk	Cell-free supernatant	*B. velezesis* QSE-21	3 d, 25°C	67.3	Disease lesion	Xu et al. (2021)
Spores suspension	Cell-free supernatant	*Pseudomonas* sp. QBA5	3 d, 23°C	81.2	Disease lesion	Gao et al. (2018)
Mycelial disk	Bacterial culture	*P. protegens* ML15	4 d, 25°C	53.8	Disease lesion	Present study
Mycelial disk	Cell-free supernatant	*P. protegens* ML15	4 d, 25°C	51.7	Disease lesion	Present study

In the present research, inoculation of *P. protegens* ML15 not only control the *B. cinerea*-caused post-harvest gray mold rot, but also have positive effect on keeping the quality of tomatoes. However, Bu et al. (2021) reported that the inoculation of *Bacillus subtilis* L1-21 had significant effect to control *B. cinerea* on tomatoes but there were no significant difference of tomatoes quality (total acid, titratable acid, vitamin C, and reducing sugar). In this research, except titratable acidity, total antioxidant, phenolic content, and ascorbic acids content were mostly improved after bacterial inoculation compared to the tomatoes infected by *B. cinerea*. According to Dhall and Singh, the advancement of the ripening period affected the nutrient content in tomatoes, the ascorbic acid content had a tendency to increase, differently with titratable acid had declined trends (Dhall and Singh, 2016). Total antioxidant was affected by phenol and ascorbic acids content because both compounds are the major contributor to the antioxidant capacity (Vallverdú-Queralt et al., 2011). Tomato is a rich source of water, mineral nutrients, sugar, vitamin, and bioactive compounds (Chen et al., 2022). Consequently, bacterial or fungi inoculation on tomatoes resulted in lower quality than no inoculation because bacteria or fungi utilized tomato nutrients for growth.

## 5 Conclusion

The PGPB isolate, *Pseudomonas protegens* ML15, has showcased remarkable antagonistic activity against various plant pathogenic fungi, *Botrytis cinerea*. This compelling evidence is further reinforced by the analytical analysis of the cell-free supernatant/extract, which confirmed the presence of several potent antimicrobial compounds such as HCN, siderophore, biosurfactant, ammonia, exopolysaccharides, lipase, 2,4-DAPG, 1-tetradecene, tetradecane, 2,6-di-tert-butylquinone, cyclododecane, 2,4-di-tert-butylphenol, and 2-methyl-1-hexadecanol. The outcomes of the complete genome study of *P. protegens* ML15 further substantiated these findings. Additionally, it was observed that the inoculation of *P. protegens* ML15 significantly curtailed the incidence of gray mold, a devastating disease caused by the pathogenic fungus *B. cinerea*, while simultaneously enhancing the postharvest quality of tomatoes. These findings collectively emphasize the significant potential of ML15 in mitigating the damage inflicted by gray mold disease on tomatoes. Further studies focusing on the optimization, purification, and utilization of the antifungal compounds derived from *P. protegens* ML15, as well as the development of formulations to extend the storage period for postharvest fruits, would be intriguing avenues for exploration.

## Data availability statement

The data presented in the study are deposited in the NCBI GenBank repository under BioProject reference no: PRJNA943471.

## Author contributions

NA: Conceptualization, Formal analysis, Investigation, Writing – original draft. AF: Formal analysis, Writing – review & editing. MD: Formal analysis, Investigation, Writing – review & editing. RS: Formal analysis, Investigation, Writing – review & editing. JP: Writing – review & editing. LD: Writing – review & editing. KP: Conceptualization, Funding acquisition, Project administration, Supervision, Writing – review & editing.
