# Supplementary material for: Biocontrol potential of Pseudomonas protegens ML15 against Botrytis cinerea causing gray mold on postharvest tomato (Solanum lycopersicum var. cerasiforme)
Source: Front Plant Sci. 2023 Dec 7;14:1288408. doi: 10.3389/fpls.2023.1288408 (PMC10748600; doi:10.3389/fpls.2023.1288408)
Supplement: Supplementary file 1 [file DataSheet_1.docx]

Supplementary Material


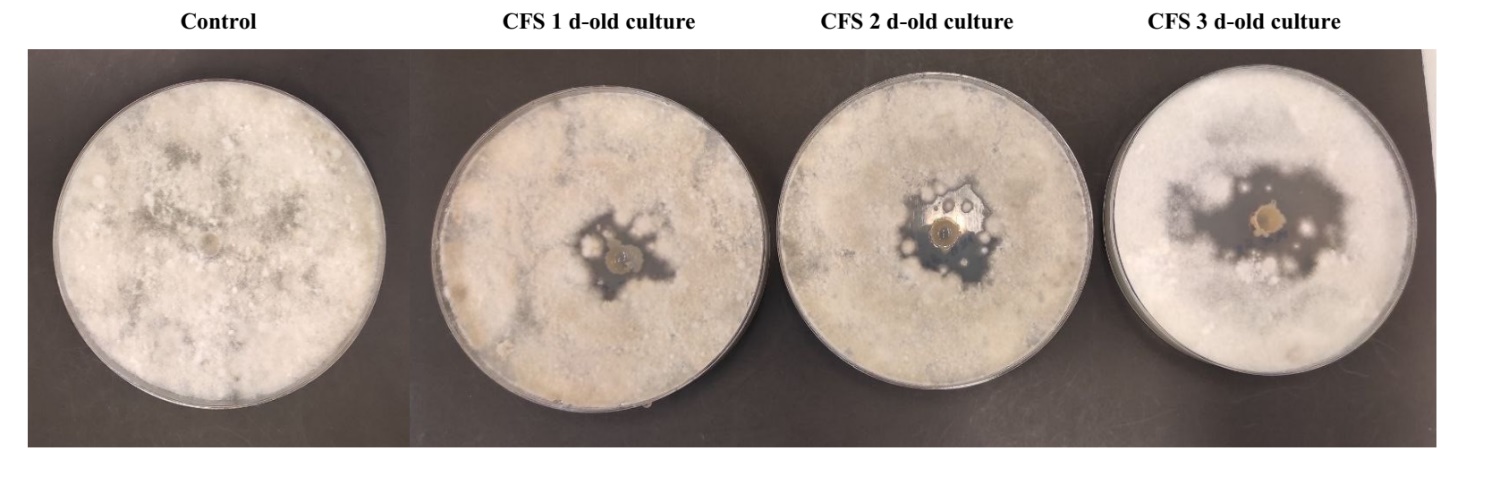


**Supplementary Figure 1.** Antagonistic activity of CFS from 1, 2, 3 d-old culture of *P. protegens* ML15 against *B. cinerea* BC21.


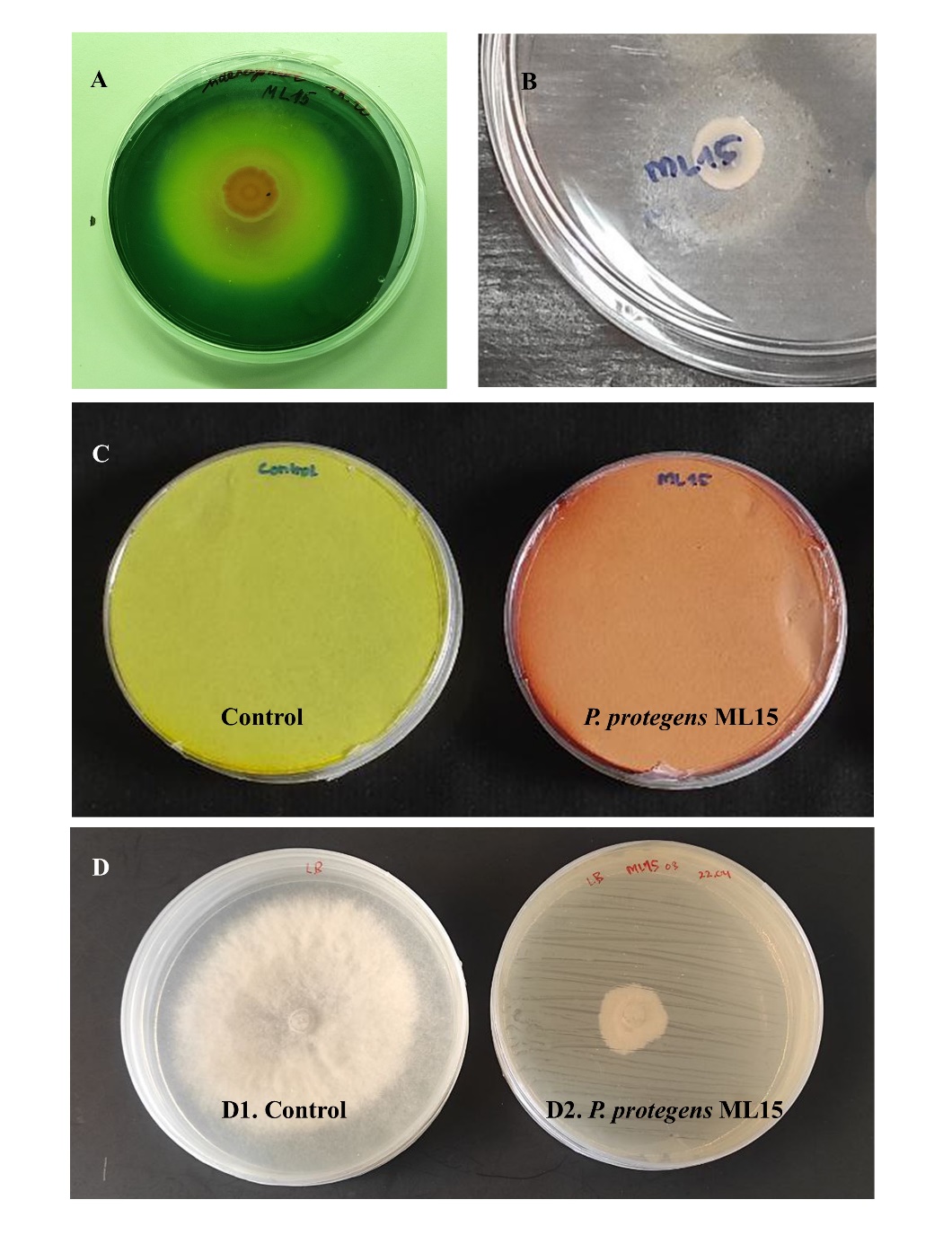


**Supplementary Figure 2.** Evidence of various biocontrol activities of *P. protegens* ML15. [**A.** siderophores production, confirmed by yellow halo on CAS agar plate; **B.** Lipase production, confirmed by the clear halo formation around the bacterial colony; **C.** HCN production, confirmed by developing of brown color in picric acid-soaked filter paper; **D.** Inhibition of *B. cinerea* mycelia growth as a result of VOCs from *P. protegens* ML15. D1. *B. cinerea* culture on solid media as control; D2. The Petri plate with *P. protegens* ML15 was inverted over the plate inoculated with the *B. cinerea*]


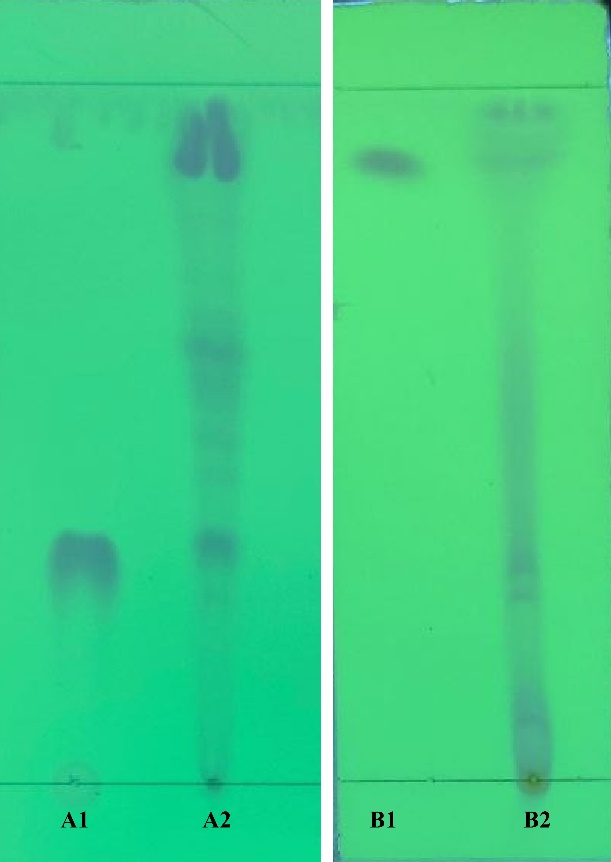


Phloroglucinol

**Supplementary Figure 3.** TLC profile of antifungal metabolites from *P. protegens* ML15. [**A1**. Phloroglucinol standard; **A2**. Cell-free extract of *P. protegens* ML15]


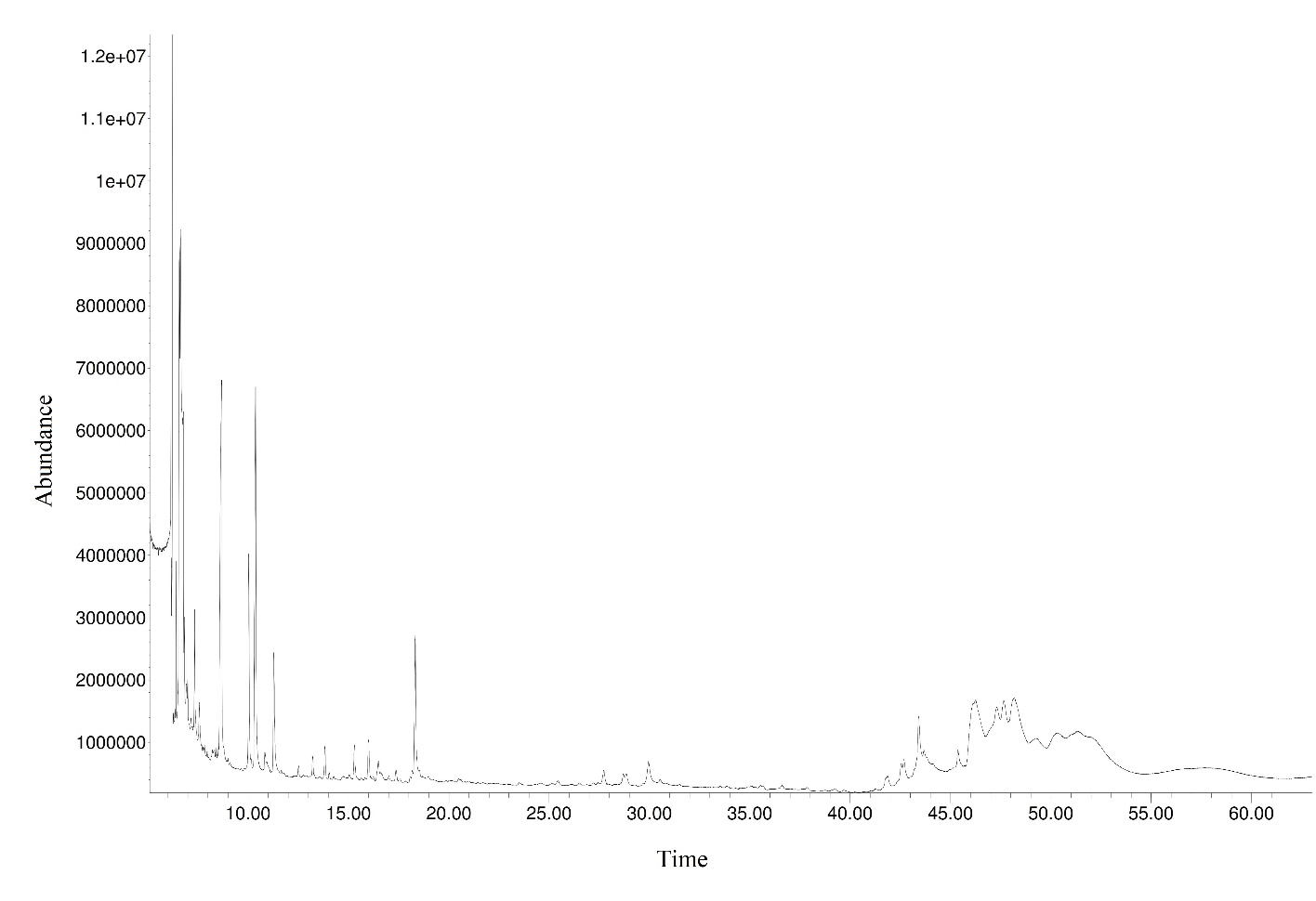


**Supplementary Figure 4.** GC-MS chromatogram of ethyl acetate extract from *P. protegens* ML15.

**Supplementary Table 1.** Predicted secondary metabolism gene clusters identified by antiSMASH in the genome of *P. protegens* ML15.

| No | Contig ID | From [bp] | To [bp] | Most similar known cluster | Similarity |
| --- | --- | --- | --- | --- | --- |
| 1 | NODE_1_length_2433100_cov_34.769616 | 116.482 | 189.438 | Orfamide A/orfamide C | 94% |
| 2 | NODE_1_length_2433100_cov_34.769616 | 860.733 | 913.482 | Pyoluteorin | 100% |
| 3 | NODE_1_length_2433100_cov_34.769616 | 1,718,569 | 1,767,037 | Enantio-pyochelin | 100% |
| 4 | NODE_1_length_2433100_cov_34.769616 | 1,865,995 | 1,907,080 | Pyrrolnitrin | 100% |
| 5 | NODE_1_length_2433100_cov_34.769616 | 2,238,494 | 2,261,708 | Fengycin | 13% |
| 6 | NODE_1_length_2433100_cov_34.769616 | 2,388,216 | 2,433,100 | Pf-5 pyoverdine | 16% |
| 7 | NODE_2_length_1127941_cov_43.754770 | 721.784 | 742.533 | MA026 | 3% |
| 8 | NODE_3_length_1059278_cov_42.697707 | 464.509 | 514.316 | Petrichorin A/petrichorin B | 4% |
| 9 | NODE_3_length_1059278_cov_42.697707 | 814.204 | 829.036 |  | N/A |
| 10 | NODE_3_length_1059278_cov_42.697707 | 974.446 | 1,027,462. | Pf-5 pyoverdine | 16% |
| 11 | NODE_4_length_812965_cov_50.857696 | 69.975 | 92.14 | Lankacidin C | 13% |
| 12 | NODE_4_length_812965_cov_50.857696 | 363.731 | 404.78 | 2,4-diacetylphloroglucinol | 100% |
| 13 | NODE_5_length_699929_cov_49.697822 | 357.529 | 401.146 | APE Vf | 40% |
| 14 | NODE_7_length_325046_cov_38.810611 | 68.866 | 89.432 |  | N/A |
| 15 | NODE_9_length_59490_cov_39.510587 | 19.612 | 59.49 | Pf-5 pyoverdine | 29% |
| 16 | NODE_12_length_10115_cov_41.754606 | 1 | 10.115 | Crochelin A | 16% |
| 17 | NODE_14_length_4239_cov_40.905399 | 1 | 4.239 |  | N/A |
| 18 | NODE_14_length_4239_cov_40.905399 | 1 | 2.532 | Nostocyclopeptide A2 | 28% |

**Supplementary Data File 1:** Details of sequences of the genes identified in the *P. protegens* ML15 genome

>ureB fig|380021.185.peg.5482 Urease alpha subunit (EC 3.5.1.5)

ATGAAGATTTCCCGTGAAGCCTACGCCGACATGTTCGGCCCCACCGTAGGCGACAAGGTG

CGGCTGGCCGACACCGAGCTGTGGATCGAAGTGGAGAAGGATTTCACCACCTACGGTGAA

GAAGTGAAGTTCGGCGGCGGCAAGGTGATCCGTGACGGTATGGGCCAGAGCCAGTTGCTG

GCCGCCGAGGTGGTGGACACCCTGATCACCAACGCCCTGATCATCGACCACTGGGGCATC

GTCAAGGCCGACGTCGGCCTCAAGGACGGGCGCATCGCCGCCATCGGCAAGGCCGGCAAT

CCGGACATCCAGCCCGACGTGAACATCGCCATCGGCGCCAGCACCGAGGTCATCGCCGGT

GAGGGCATGATCCTCACCGCCGGCGGCATCGACACCCATATCCACTTCATCTGCCCGCAG

CAGATCGAAGAGGCGCTGATGAGCGGCGTCACCACCATGATCGGTGGCGGCACGGGACCT

GCCACCGGGACCAACGCCACCACCTGCACCTCCGGGCCCTGGCACATGGCGCGCATGCTC

CAGGCGGCGGACGCCTTCGCCATGAACATCGGCTTTACCGGCAAGGGCAACGCCAGCCTG

CCGGAGCCCTTGATCGAACAGGTCAAGGCCGGGGCCATCGGCCTCAAGCTGCACGAGGAC

TGGGGCAGCACCCCGGCCAGCATCGACAACTGCCTGAGCGTGGCCGATGAGTACGACGTG

CAGGTGGCGATCCACACCGACACCCTCAATGAATCGGGCTTCGTCGAAACCACCCTGGGC

GCCTTCAAGGGCCGCACCATCCATACCTACCACACCGAAGGCGCCGGCGGCGGCCATGCC

CCGGACATCATCAAGGCCTGCGGCCTGGCCAACGTGCTGCCCAGCTCCACCAACCCGACC

CGGCCCTTCACCCGCAACACCATCGACGAACACCTGGACATGCTGATGGTCTGCCATCAC

CTGGACCCGAGCATCGCCGAGGACGTGGCCTTCGCCGAAAGCCGCATCCGCCGGGAGACC

ATTGCCGCCGAGGACATCCTCCACGACCTCGGGGCCTTCTCCATGATCAGCTCCGACAGC

CAGGCCATGGGCCGGGTCGGCGAAGTGGTGACCCGCACCTGGCAGACCGCCGACAAGATG

CACAAGCAGCGCGGCCCGCTGCCGGGGGATGGCCCGGGCAACAGCAACTTCCGGGTCAAG

CGCTACATTGCCAAGTACACCATCAACCCGGCGATCACCCACGGCATCAGCCATGAGGTG

GGCTCCGTCGAAGTGGGCAAGTGGGCCGACCTGGTGCTCTGGCGCCCGGCGTTCTTCGGG

GTCAAGCCGACCCTGATCCTCAAGGGCGGGGCCATTGCCGCCAGCCTGATGGGCGACGCC

AACGCCTCGATCCCGACCCCGCAGCCGGTGCACTACCGGCCGATGTTCGCCAGCTTCGGC

GGCAGCCGTCACGCCACCAGCCTGACCTTCATCAGCCAGGCGGCACTGGAGGCCGGGGTG

CCGGAGCAGTTGGGGCTGAAAAAACGCATCGCCGTGGTCAAGGGCTGTCGCAACGTGCAG

AAAACCGACCTGATCCACAACGACTACCTGCCGGATATCGAAGTCGACCCGCAGACCTAT

CAGGTCAAGGCTGATGGCGTCCTGCTGTGGTGCGAACCGGCGGACGTGCTGCCCATGGCC

CAGCGCTACTTCCTGTTCTAG

>pqqB fig|380021.185.peg.4273 Coenzyme PQQ synthesis protein B

ATGTTTGTCCAGATTCTAGGTTCCGCCGCCGGCGGTGGTTTTCCGCAGTGGAACTGCAAC

TGTGTGAACTGCGCCGGATTTCGCGACGGTAGCCTGCGGGCCCAGGCGCGCACTCAATCG

TCCATCGCGATTTCCGATGACGGGGTGAACTGGGTGCTGTGCAACGCCTCTCCGGATATC

CGCGCGCAGTTGCAAAGCTTCGCCCCCATGCAACCGGGCCGCGCACTGCGGGACACCGGC

ATCGGCGCCATCATCCTCATGGACAGCCAGATCGACCATACCACCGGCCTGCTCAGCCTG

CGTGAAGGCTGCCCGCACCAGGTCTGGTGCACCGACATGGTCCATGAAGACCTGAGCACC

GGTTTCCCGCTGTTCAAGATGCTCAGCCACTGGAACGGCGGCCTGAGCTGGAACCGCATC

GAGCTGGACCAGAGCTTCAGCATCCCCGCCTGCCCCAACCTGCGCTTCACGCCGCTGCCC

CTGCGCAGCGCAGCGCCGCCCTATTCGCCCCACCGTTTCGACCCGCACCCGGGGGACAAC

ATCGGCCTGATCGTCGAAGACCTGCGCAGCGGCGGCAAACTCTTCTACGCGCCGGGCCTG

GGCAAGGTCGACGCGCCACTCCTGGAGATCATGGCCGCCAGCGATGTGCTGCTGGTGGAC

GGCACCCTGTGGGAAGACGATGAAATGCAGCGTCGTGGCGTGGGCACCCGTACCGGCCGG

GAAATGGGCCACCTGGCGCAGAACGGCCCCGGCGGCATGCTCGAAGTGCTGGAACAGTTG

CCGCGCCAGCGCAAGGTGCTGATACATATCAACAACACCAACCCGATCCTCGATGAAGAC

TCCGCCGAACGTGCCGAACTGGTACGCCGAAAGGTGGAAGTGGCTTACGACGGAATGAGT

ATTGAGCTGTAG

>pqqC fig|380021.185.peg.4274 Pyrroloquinoline-quinone synthase (EC 1.3.3.11)

ATGACCGACACCCCGCTGTCCGCCGCCGAATTCGAGCAGGCACTGCGCGCCAAAGGCGCC

TACTACCACATCCATCACCCTTATCACGTGGCGATGTATGAAGGCCGGGCGACCCGCGAG

CAGATCCAGGGCTGGGTCGCCAACCGCTTCTATTACCAGGTGAACATCCCCATGAAGGAC

GCGGCGATCCTCGCCAACTGTCCGGACCGGGAGATTCGCCGCGAATGGATCCAGCGCCTG

CTGGACCATGACGGCGCCCCGGGCGAGGACGGCGGCATCGAGGCCTGGCTGCGCCTGGGC

CAGGCCGTGGGCCTGGACCCGGACCAGTTGCGCTCCCAGGAACTGGTGCTGCCCGGTGTG

CGCTTTGCCGTGGACGCCTATGTCAATTTTGCTCGCCGGGCCAGTTGGCAGGAGGCGGCC

AGCAGCTCGCTGACCGAACTGTTCGCGCCGCAAATCCACCAGTCGCGCCTGGACAGCTGG

CCCCAGCATTACCCATGGATCGACCCCACTGGCTACGAGTACTTCCGCACCCGCCTGGGC

CAGGCCCGCCGCGATGTCGAGCACGGCCTGGCGATCACCCTGCAGCACTACACCACCCGT

GCCGGCCAGGAACGCATGCTGGAAATTCTCCAGTTCAAACTGGACATCCTTTGGAGCATG

CTCGACGCCATGAGCATGGCCTATGAACTGAACCGCCCGCCCTATCACAGCGTCACCGAT

CAGCGGGTCTGGCATAAGGGGATCACCCTATGA

>pqqE fig|380021.185.peg.4276 Coenzyme PQQ synthesis protein E

GTGCTCAGCACTGGATCGAACTCGCCTGAACCGGCGGTTCTGCCCGGCAAACCGGAAGTT

GGCCTGCCGCTGTGGCTGCTGGCCGAGCTGACCTACCGCTGCCCGCTGCAGTGCCCCTAC

TGCTCCAACCCCCTGGACTTCGCCGAGCAGGGCAAGGAGCTGAGCACCGAGCAGTGGATC

AAGGTATTTCGCGAAGCGCGGGAAATGGGTGCAGCGCAGCTGGGTTTCTCCGGCGGCGAG

CCGCTGGTGCGCCAGGACCTGGCCGAACTGATCGGCGAGGCGCGCAAGCTGGGCTTCTAT

ACCAACCTGATCACCTCCGGCATCGGCCTCACCGAGCAGAAGATCAGCGACTTCAAGAAA

GCCGGCCTGGACCATATCCAGATCAGCTTCCAGGCCAGCGACGAACAGGTGAACAACCTG

CTGGCCGGTTCGAAAAAAGCCTTCGCGCAGAAGCTGGAAATGGCCCGTGCGGTCAAGGCC

CACGGCTATCCGATGGTGCTGAACTTCGTCACCCACCGGCACAACATCGACAAGATCGAC

CGCATCATCGAGCTGTGCATCGCCCTGGAAGCCGATTTCGTCGAGCTCGCCACCTGCCAG

TTCTACGGCTGGGCCCAGCTCAACCGCGTGGGCCTGCTGCCGACCAAGGAACAACTGGTG

CGCGCCGAGCGCATCACCAACGAATACCGCGCCAGGCTGGAAGCCCAAGGGCATCCCTGC

AAGCTGATCTTCGTCACTCCGGACTACTACGAGGAGCGCCCCAAGGCCTGCATGAATGGC

TGGGGCAGCATCTTCCTCACCGTGACTCCGGACGGCACCGCACTGCCCTGCCACGGCGCG

CGGCAGATGCCGGTGCAGTTCCCCAATGTGCGCGACCACAGCATGCAGCACATCTGGTAC

GACTCATTCGGTTTCAACCGCTTTCGCGGGTACGACTGGATGCCCGAACCCTGCCGCTCC

TGCGACGAGAAGGAAAAGGACTTCGGCGGCTGCCGCTGCCAGGCCTTCATGCTCACCGGA

GATGCCAGCAATGCCGATCCGGTGTGCAGCAAATCGCCACACCATGGGATGATCCTCCAG

GCCCGTGAAGAATCTGAAACGGCCACCCACACCATCGAGCAACTGGCTTTTCGCAATGAA

CGAAACTCCCGCCTCATCGCCAAGGGTTGA

>pstB fig|380021.185.peg.4731 Phosphate ABC transporter, ATP-binding protein PstB (TC 3.A.1.7.1)

ATGCAACACGAAGCACACACTCATGGCATCAACATGTCCGCCCTGGGCCGCGACAAGCAG

AGCCTGAGCCTGGAACAGGAAACCGTGGCCATCGAAGTGCCCGGCCTGAGCCTGTACTAC

GGCGAGAAACAAGCGCTGTTCGACGTCAGCATGAACATCCCCAAGCAGCGCGTGACCGCC

TTCATCGGCCCGTCCGGTTGCGGCAAGTCGACCCTGCTGCGGACCTTCAACCGCATGAAC

GACCTGGTGGACGGCTGCCGCGTAGAGGGTGCGATCAACCTCTACGGCAACAACATCTAC

CGCAAGGGCGAAGACGTGGCCGAGCTGCGTCGCCGGGTCGGCATGGTGTTCCAGAAGCCC

AACCCGTTCCCCAAGACCATCTATGAAAACGTGGTCTACGGCCTGCGCATCCAGGGCATC

AACAAGAAGCGCGTGCTCGACGAAGCCGTGGAATGGGCCCTCAAGGGCGCGGCGCTGTGG

GACGAGGTCAAGGACCGCCTGCATGATTCGGCCCTGGGCCTGTCCGGCGGCCAGCAGCAG

CGTCTGGTAATCGCCCGGACCATCGCCGTGGAGCCGGAAGTGCTGCTGCTGGACGAACCC

TGCTCGGCCCTGGACCCGATCTCGACCCTGAAGGTCGAGGAGCTGATCTATGAGCTCAAG

TCCAAGTTCACCATCGTCATCGTGACCCACAACATGCAGCAGGCGGCGCGGGTTTCCGAC

TACACCGCCTTCATGTACATGGGCAAACTGGTGGAGTTCGGCGATACCGACACGCTGTTC

ACCAACCCGGCGAAGAAGCAGACCGAAGACTACATTACCGGGCGCTATGGCTAG

>phlD fig|380021.185.peg.4565 1,3,6,8-tetrahydroxynaphthalene synthase (EC 2.3.1.233)

ATGTCTACACTTTGCCTTCCACACGTCATGTTTCCGCAACACAAGATCACCCAGCAACAG

ATGGTCGATCACCTGGAAAACCTGCACGCCGACCATCCACGCATGGCCCTGGCCAAGCGC

ATGATCGCCAACACCGAAGTCAACGAGCGCCACCTGGTGTTGCCGATCGACGAACTGGCA

GTGCACACCGGCTTCACCCACCGCAGCATCGTCTACGAGCGTGAAGCCCGGCAGATGTCG

TCGGCCGCGGCGCGCCAGGCCATCGAGAATGCCGGGCTGCAGATCAGCGACATTCGCATG

GTGATCGTCACTTCCTGCACCGGCTTCATGATGCCGTCGCTGACCGCGCACCTGATCAAC

GACCTGGCCCTGCCAACCTCCACCGTGCAGTTGCCGATCGCCCAGCTGGGCTGCGTGGCC

GGTGCCGCGGCCATCAACCGCGCCAACGACTTCGCCCGGCTCGATGCCCGCAACCACGTA

CTGATCGTGTCCCTGGAGTTCTCCTCGCTGTGCTACCAGCCGGACGACACCAAGCTGCAC

GCCTTCATCTCCGCGGCGCTGTTCGGCGATGCGGTATCCGCCTGCGTGCTGCGCGCCGAT

GACCAGGCCGGCGGCTTCAAGATCAAGAAGACCGAGTCGTACTTCCTGCCCAAGAGCGAG

CACTACATCAAGTACGACGTGAAGGACACCGGCTTTCACTTCACCCTCGACAAGGCGGTG

ATGAACTCCATCAAGGACGTGGCACCGGTCATGGAGCGGCTGAACTACGAGAGCTTCGAA

CAGAACTGTGCGCACAACGACTTCTTCATCTTCCACACCGGTGGTCGCAAGATCCTCGAC

GAGCTGGTGATGCACCTGGACCTGGCATCCAACCGGGTCTCGCAATCGCGCAGCAGCCTG

TCGGAAGCCGGCAACATTGCCAGCGTGGTGGTGTTCGACGTGCTCAAGCGGCAGTTCGAT

TCCAACCTCAATCGCGGCGACATCGGCCTGCTGGCGGCCTTCGGCCCGGGGTTCACCGCG

GAAATGGCGGTGGGCGAGTGGACCGCCTGA

>hcnA fig|380021.185.peg.627 Hydrogen cyanide synthase HcnA @ Opine oxidase subunit C

ATGCGTCAGATAGACCGCAACTTCGATATTCAGCCGCTGCAGCATGCGGACATGACCATC

AGCCTCAATGGCCAGCCAGTCACTGCCGCCCTGGGTGAAACCGTCCTCAGCGTGATCCAG

GCCACCGGCCTGCGCCAGGTGGCGCGCAACGATCACGGGCAACTGGTCGGCGCCTACTGC

GGCATGGGCGTATGCCATTGCTGCCTGGTGCAGATCGACGGCCGGCACAAGCGCCGAGCC

TGCCAGACCCTGGTCAAGCCGGGGATGCAGGTGCAGACCCTGAGCAACCGCATCACTGAA

ACGGAGCCCACGTTATGA

>hcnB fig|380021.185.peg.628 Hydrogen cyanide synthase HcnB @ Opine oxidase subunit A

ATGAGCCTGAACCCGGTGATCGTCGGCGGTGGGCCGGCGGGCATGGCGGCCGCCATCGAA

CTGGCCGAGCACGGCGTGCGCAGCACCCTGATCGAAGAGGCCTCGCGCCTGGGGGGCGTG

GTCTACCGCGGGCCGCTGCGCGACGGCGTGCAGCTGGATTACCTGGGGCCGCGCTACTGC

GAAATGCTGGCCAAGCTGCACGGCGATTTCGCCGACCACGAACAGATGATCGACGTGCGC

CTCAACAGCCGGGTAGTGGGCGCCGAGGGCACCCAGAGCCTGGTGCTGCTCGATGGCGAA

GAGCAGGTGCAGCAGGTCAGCTACGAGCAGTTGATACTGGCCGCCGGCTGCCATGAGCGC

AGTGTGCCGTTCCCCGGCTGGACCCTGCCCGGGGTCAAGCTGCTGGGTGGCCTGCAATTG

CAGATCAAGAGCGGGGTGGTCAAGCCCCAGAGCCCGGTGGTGATCGCCGGTACCGGACCC

TTGCTGCCCCTGGTGGCGTGCCAGCTGCATGCTTCCGGCGTGCGCGTGGCGGGGGTCTAC

GAGGCTTGTGCCCTGGGCAAGATCGCCAAGCAGAGCCTGGCCATGCTCAACAAGCCGCAA

CTGTTTCTCGACGGCCTGAGCATGCTCGCCTACCTCAAGCTGCACGGCATTGCCCTGCGC

TATGGCTGGGGCGTGGTCGAAGCCCAGGGCCAGGACGCCCTGAGCGTGGTCACCGTGGCG

CCGTACTCCAGCGACTGGCAGCCGGACATGGCCAAGGCCCAGCGCATTGCTGCCCAGACC

CTGGCGGTGGGCTACGGCTTCATCCCGCGCACCCAGTTGAGCCAGCAGATGGGCCTGGAA

CACAACTTCAGCGACGACGGCTACCTGCGCGCCAGCGCCAACGCCTGGCAGCAGAGCAGC

GAGCCCCACGTGCACCTGGCCGGCGACATGGGCGGCATCCGCGGCGGCGAGGCGGCCATG

CTCAGCGGGCGTATCGCCGCGCTGTCGATCCTCATGCAGCGTGGCGTACTGAGCAACGAA

GCGGCCCTGCAACGGCGCCAGGGTTACGAGCGCAAGCTGGCCTCGATCCTGCGTTTTCGT

GGTGCGGTGGACCGCTATACCGCGCGTGGCGCCGGGCAGGTAGAGCTGCCCAAGGGCGAC

ACCGTGATCTGCCGTTGCGAGCACACCACCCGCAACGATATCGAGCGTGCCCTGAGCCAG

GGCGTGCAGGACATGGCCAGCCTGAAAATGCGCACCCGGGTGAGCATGGGCGACTGCCAG

GGGCGCATGTGCGTGGGCTATTGCAGCGACCGCCTGCGCCAGGCCACCGGGCGCAAGGAC

GTGGGCTGGATCCGCCCGCGCTTCCCCCTGGACCCGATTCCGTTTTCCGCGTTCCCGCCG

TCTGACCAGGAGGTCTCCCAGCATGATTAA

>hcnC fig|380021.185.peg.629 Hydrogen cyanide synthase HcnC @ Opine oxidase subunit B

ATGATTAAGCATTACGACGTGGTCATTGCCGGTGGCGGCGTGATCGGCGCTTCCTGCGCC

TACCAGTTGTCCAAGCGCAAGGACCTGAAGGTGGCGCTGATCGACGCCAAGCGCCCGGGC

AACGCCAGCCGCGCTTCGGCCGGCGGGCTGTGGGCCATCGGCGAGTCGGTGGGGCTGGGC

TGCGGGGTGATCTTCTTTCGCATGATGTCGGCCAACCGCAAGCGCGAGGCCCAGGGTTCG

GCGGTGGTGGTGGATTCCAGCACTCCGCACATCCTGCCGCAGTCGTTCTTCGACTTCGCC

CTGCAGTCCAACGAGTTGTACCCGCGCCTGCACCGCGAGCTGATGGGCCTGCACAACATG

GATTTCAAGTTCGAGCAGACCGGGCTCAAGTTCGTCATCTATGACGAGGAAGACCGCCTG

TACGCCGAGCACATCGTCGGCTGCATCCCGCACCTGAGCGACCAGGTACGCTGGCTCGAC

CAGGCGGCCCTGCGCGCCGCGGAACCCAACGTCAGCCATGAAGCCCAGGGCGCCCTGGAG

TTCCTCTGCGACCACCAGGTCAACCCGTTCCGCCTCACCGACGCCTACACCGAAGGCGCA

CGCCAGAACGGCGTGGATGTGTACTTCAACACCAACGTCACCGGGGTCCTGCACCAGGGC

AATCGGGTCAGCGGGGTGAAGACCGACGTTGCCGGGTTGTTCCGCTGCACGACCCTGATC

AACGCCGCCGGCGCCTGGGCCGCCGAGCTGAGCCTGCAGGCCACGGGCATCGAGATTCCG

GTGAAGCCGGTGAAGGGCCAGATCCTTCTCACCGAGCGCATGCCCAAGCTGCTCAACGGC

TGCCTGACCACCAGCGACTGCTACATGGCGCAAAAGGACAACGGCGAGATCCTGATCGGC

AGTACCACCGAGGACAAGGGCTTCGACGTCACCACCACCTACCCGGAGATCAACGGCCTG

GTGCAGGGCGCAGTGCGCTGCGTACCGGAGCTGGCCCATGTCAACCTCAAGCGCTGCTGG

GCGGGGTTGCGCCCGGGTTCGCCGGATGAGCTGCCGATCCTCGGGCCGATGGATGGGGTC

GAAGGCTACCTCAATGCCTGCGGGCACTTCCGCACCGGCATCCTGACCTCGGCCATCACC

GGCGTGTTGCTGGACAAACTGGTGAACGACGAAGCCCTGCCGCTGGACATCACCCCCTTC

CTGGCCCGGCGCTTCGCCACTGCCCCCGTGAAGAAACAGCCCGAACCGGCCTGA

>nirK fig|380021.185.peg.5679 Copper-containing nitrite reductase (EC 1.7.2.1)

ATGGGTAGCTTTCAAACGTGGTTGAAGTGCTGTGTGGTGGCCGGCAGCTGTGCCTCCGGC

CTGGTGTTGGCCGCCGGGGCCGATGGCTTGCAGAGGGTCAAGGTTGACCTGGTGGCGCCG

CCCCAGGTGCATGTCCATGAGCAGGCCGTCAACGGGCCGCCGAAGGTCGTGCAGTTCCGC

ATGAATGTGGAAGAGAAAAAGATGGTGGTGGATGACCAGGGCACCACCTTGCAGGCCATG

ACCTTCAACGGTTCCATGCCCGGCCCGACCCTGGTGGTGCACGAGGGCGACTACGTCGAG

CTGACCTTGAGCAATCCGGCCAGCAACAGCATGCCCCATAACATCGACTTTCATGCGGCC

ACCGGGGCCCTGGGCGGGGCGGCCCTGACTCAGGTTTTGCCGGGGCAGGAAGTGGTGCTG

CGCTTCAAGGCCGACCGCAGCGGAACCTTCGTCTACCACTGCGCACCACCGGGCATGGTG

CCCTGGCACGTGGTCTCGGGCATGAGCGGGACCTTGATGGTGCTACCGCGCGAAGGCCTG

AAAGACCCTGCCGGCAAACCCTTGCACTATGACCGCGCCTACACCATCGGCGAGTTCGAC

CTGTACATTCCCAAGGACAAGGACGGGCACTACAAGTCCTACCCGGACCTGGCCTCCAGC

TACCAGGACACCCGCGAGGTGATGCGTACGTTGACCCCGAGCCATGTGGTGTTCAACGGA

CGGGTTGGCGCTCTGACCGGGGCCAACGCGCTGACCGCCAAGGTCGGCGAAAGCGTGCTG

TTCATCCACTCCCAGGCCAACCGCGACAGCCGCCCGCACCTGATTGGCGGCCATGGCGAC

TGGGTCTGGACCACCGGCAAGTTCGCCAATGCCCCGCAACGCAACCTGGAGACCTGGTTC

ATCCCCGGCGGCTCGGCGTTGGCGGCGCTCTACACCTTCAAGCAGCCGGGCACCTACGTG

TACCTCAACCACAACCTGATCGAGGCCATGGAGCTGGGGGCGCTGGCCCAGGTCAAGGTC

GAAGGGCAGTGGGACGATGACCTGATGACCCAGGTAAAGGCCCCCGGGCCCATTACTCCG

GCCAAGTGA

>gabT fig|380021.185.peg.5030 5-aminovalerate aminotransferase (EC 2.6.1.48) / Gamma-aminobutyrate:alpha-ketoglutarate aminotransferase (EC 2.6.1.19)

ATGAGCAAGACCAACGCATCTTTGATGAAACGCCGTGAAGCCGCTGTACCGCGCGGTGTT

GGCCAGATTCACCCGATCTTCGCCGACAGCGCGAAGAACGCCACCGTCACCGACGTTGAA

GGCCGCGAGTTCATCGACTTCGCCGGCGGTATCGCGGTACTGAACACCGGTCACGTGCAC

CCGAAGATCATCGCCGCGGTGACCGAGCAACTGAACAAGCTGACCCACACCTGCTTCCAG

GTCCTGGCCTACGAGCCTTACGTGGAGCTGTGCGAGAAAATCAACGCCAAGGTGCCAGGT

GATTTCGCCAAGAAAACCCTGCTGGTGACCACCGGTTCCGAAGCCGTAGAGAACGCCGTG

AAGATCGCCCGTGCCGCCACTGGCCGCGCCGGCGTGATCGCCTTCACCGGCGCCTACCAC

GGCCGCACCATGATGACCCTGGGCCTGACCGGCAAGGTCGTGCCTTACTCGGCCGGCATG

GGCCTGATGCCAGGCGGCATCTTCCGCGCGCTGTACCCGAATGAACTGCACGGCGTGAGC

ATCGACGACTCCATCGCCAGCATCGAGCGCATCTTCAAGAACGACGCCGAGCCTCGTGAT

ATCGCCGCGATCATCATCGAGCCGGTGCAGGGCGAAGGCGGCTTCTACGTCGCGCCCAAA

GAGTTCATGAAGCGCCTGCGCGCCCTGTGCGACCAGCACGGCATCCTGCTGATCGCTGAC

GAAGTGCAGACCGGCGCTGGCCGTACCGGCACCTTCTTCGCCATGGAACAGATGGGCGTT

ACCGCCGACCTGACCACCTTCGCCAAGTCCATCGCCGGCGGCTTCCCGCTGGCCGGTGTC

TGCGGCAAGGCCGAGTACATGGACGCCATCGCTCCAGGCGGCCTGGGCGGCACCTACGCC

GGTAGCCCGATCGCCTGTGCGGCGGCCCTGGCGGTGATGGAAGTGTTCGAGGAAGAGCAC

CTGCTGGATCGCTGCAAGGCTGTTGGCGAGCGCCTGGTGACCGGCCTCAAGGCCATCCAG

GCCAAGTACCCGGTGATCGGTGAAGTGCGTGCCCTGGGCGCGATGATCGCGGTGGAACTG

TTCGAGAATGGCGACAGCCACAAGCCGAACGCCGCTGCCGTGGCCCAGGTCGTGGCCAAG

GCTCGCGACAAGGGGCTGATCCTGCTGTCCTGCGGCACCTACGGCAACGTTCTGCGGGTT

CTGGTACCGCTGACCTCGCCGGATGCCCAGCTGGACAAAGGCCTGGCCATCATCGAAGAG

TGCTTCGCTGAACTCTGA

>gabD fig|380021.185.peg.5029 Succinate-semialdehyde dehydrogenase

ATGCAGCTCAAAGACGCCCAGTTGTTCCGCCAACAAGCCTTCATCGATGGAGCTTGGGTC

GACGCGGACAACGGCCAGACGATCAAGGTCAACAACCCCGCTACCGGTGAAATCCTCGGT

ACCGTGCCAAAGATGGGCGCTGCCGAAACCCGCCGCGCCATCGAGGCCGCCGACAAGGCC

CTGCCGGCCTGGCGTGCCCTGACCGCCAAGGAGCGCGCCACCAAGCTGCGCCGCTGGTAT

GAATTGCTGATCGAGAACCAGGACGACCTGGGTCGCCTGATGACCCTGGAGCAGGGTAAG

CCGCTGGCCGAAGCCAAGGGCGAAATCGCCTACGCCGCCTCCTTCATCGAATGGTTCGCC

GAAGAAGCCAAGCGCATCTACGGCGACGTGATTCCCGGCCACCAGCCCGACAAGCGCCTG

ATCGTGATCAAGCAGCCGATCGGCGTGACCGCGGCCATCACCCCGTGGAACTTCCCGGCG

GCGATGATCACCCGTAAAGCCGGCCCGGCCCTGGCCGCCGGTTGCACCATGGTCATCAAG

CCCGCTTCGCAAACCCCGTTCTCGGCCCTGGCCCTGGTGGAACTGGCGCACCGTGCCGGC

ATCCCGAAAGGCGTGCTGAGTGTGGTCACCGGCAGCGCCGGCGACATCGGCGGCGAGCTC

ACCAGCAACCCGATCGTGCGCAAGCTGTCGTTCACCGGCTCCACCGAGATCGGTCGCCAG

CTGATGGCCGAATGCGCCAAGGACATCAAGAAAGTCTCCCTGGAGCTGGGCGGCAACGCG

CCGTTCATCGTGTTCGACGACGCGGACCTGGATAAGGCCGTCGAAGGCGCGATCATCTCC

AAGTACCGCAACAACGGCCAGACCTGCGTCTGCGCCAACCGCCTGTACATCCAGGATTCG

GTCTACGACGCCTTCGCCGAGAAATTGAAGGCAGCAGTGGCCAAGCTGAAGATTGGCAAC

GGCCTGGAAGAAGGCACCACCACTGGCCCGCTGATCGACGAAAAAGCCGTGGCCAAGGTC

CAGGAACACATCGCCGACGCCCTGAAAAAAGGCGCGACCCTGCTGGCCGGCGGCAAGTCC

ATGGAAGGCAACTTCTTCGAGCCGACCATCCTGGTCAACGTGCCCAAGGATGCAGCCGTG

GCCAAGGAAGAAACCTTTGGCCCACTGGCGCCGCTGTTCCGCTTCAAAGACGAAGCCGAA

GTCATCGCCATGTCCAACGACACCGAATTCGGCCTGGCCTCGTACTTCTATGCCCGCGAC

CTGGGCCGTGTGTTCCGTGTGGCCGAGGCCCTGGAATACGGCATGGTCGGGGTCAACACC

GGCCTGATCTCCAACGAAGTGGCGCCGTTCGGCGGCATCAAGGCCTCGGGCCTGGGCCGT

GAAGGTTCCAAGTACGGGATCGAGGATTACCTGGAAATCAAATACCTCTGCCTGGGTATC

TGA

>pvdH fig|380021.185.peg.4172 Pyoverdin biosynthesis protein PvdH, L-2,4-diaminobutyrate:2-oxoglutarate aminotransferase (EC 2.6.1.76)

ATGTCAGCCGTTACCAGCCTGATGGAAGATTCGCCGGTGGGCCTTGGCGCCGCCCCGCAA

GAGACGCTTTACCAGTTCGACGAGTCGCCCCTGCTGGCCCGCCAGAGCCGCCAGGAATCC

AATGCCCGCAGTTATCCACGGCGCATTCCCCTGGCCCTGAAGCGGGCCAGCGGCATTCAC

GTCGAAGACGTCGAGGGCCGGCGTTTCATCGATTGCCTGGCCGGTGCCGGGACTTTGGCC

CTGGGCCACAATCACCCGGTGGTGATCGCCGCGATCCAGCAGGTGCTGGCCGATGAGTTG

CCGCTGTTGACCCTGGACCTGACCACCCCGGTCAAGGATCAGTTCGTCCAGGACCTGTTC

GGCCTGTTGCCCGAGGCCCTGGCCGCGGAGGCCAAGATCCAGTTCTGCGGTCCCACCGGC

ACCGATGCCGTGGAAGCCGCGCTGAAGCTCGTGCGCACCGCCACCGGACGCAGCACCGTG

CTGTCGTTCCAGGGCGGTTACCACGGCATGAGCCAGGGCGCGCTGAGCCTGATGGGCAGC

CTGGGGCCCAAGCGCGCCCTGGCCGGGCTGCTGAACAACGGCGTGCAGTTCCTGCCGTTC

CCCTATGACTACCGCTGCCCGTTCGGGCTGGGGGGCGCCGAAGGGGTCAAGGTCAACCTG

CATTACCTGGAAAACCTGCTCACCGATCCGGAAGCCGGGGTGGCCCTGCCAGCGGCAGTG

ATTGTCGAGGCGGTGCAGGGCGAGGGCGGGGTGATCCCGGCCGACCTGGAATGGCTGCAA

GGTGTGCGCCGCATCACCGAGAAGGCCGGGGTGGCATTGATCGTCGATGAAATCCAGAGC

GGCTTCGGTCGCACCGGCAAGATGTTCGCCTTTGAGCACGCGGGGATCATTCCCGATGTG

GTGGTGATGTCCAAGGCCATCGGCGGCAGCCTGCCGCTGGCGGTGATGGTCTATCGCGAC

TGGCTCGACACCTGGCAGCCGGGTGCCCACGCCGGCACCTTCCGCGGCAATCAGATGGCC

ATGGCCACCGGTTCCGCGGTGATGCGCTACCTCAAGGAACACCGGGTGCCCGAGCACGCG

GCGGCCATGGGCGAGCGCTTGCGCGAGCACCTGCTGATCCTGCAGCGCGACTTCCCGCAA

CTGGGGGATATCCGTGGTCGTGGCCTGATGCTCGGGGTGGAGCTGGTGGACCCGGCCGGC

GTGCCGGATGCCCAGGGCCATCCACCCCAGCATGCGCGCCTGGCACCGCTGGTGCAGCGC

GAATGCCTCAAGCGCGGGCTGATCCTGGAGCTGGGCGGGCGCCACGGCGCCGTGGTGCGT

TTCCTGCCGCCGCTGGTAATCACCGCAGCGCAGATCGACCAGGTGGCCCAGATCTTCAGC

AAAGCCGTAGCGGCGGCAGTGGCCAGCCTCTAA

>pvdL fig|380021.185.peg.4159 Pyoverdine chromophore precursor synthetase PvdL @ Siderophore biosynthesis non-ribosomal peptide synthetase modules

ATGACGGACGCGTTCGAACTCCCCAGCACCCTGGCCCAAGCCCTTCAACGCCGGGCAGTC

CTGGCACCTGATCAAGTGGCGTTGCGCTTTCTCGCCGAAGAGCAGGATCAGAGCGTGGTC

CTCAGTTACCGGGACCTGGACCTTCGTGCGCGCACCATTGCTGCGGCTCTGCAAGCCAAC

GCCGAATTCGGCGATCGCGCGGTATTGCTGTTTCCCAGCGGCCCGGACTACGTCGCGGCG

TTCTTCGGTTGCCTGTACGCCGGGGTGATCGCGGTGCCGGCCTATCCGCCGGAGTCCACC

CGTCGTCATCACCAGGAGCGCCTGATTTCCATCATGGCCGACGCCGAGCCACGGTTGCTG

CTGACCAGCGACGGGCTGCGGGATTCACTGTTGCAGATGGACGAACTCAAGGCCCCAGGC

GCGCCGCAACTGCTGTGTGTGGACACCCTGCAAGCCGGGCTGGCCGAAGACTGGCAGGCC

GTGGCGCTGCAGGGCGACGACATCGCCTTCCTGCAATACACCTCGGGCTCCACCGCGCTG

CCCAAGGGCGTGCAGGTCAGCCATGGCAACCTGGTGGCCAACGAACTGCTGATCCGTCGC

GGCTTCGGTATCGACCTCAACCCGGACGACGTGATCGTCAGCTGGCTGCCGCTGTACCAC

GACATGGGCTTGATTGGCGGCCTGCTGCAACCGATTTTCAGCGGCGTGCCCTGTGTGCTG

ATGTCACCGGCCTACTTCCTGGCGCGGCCCGTACGCTGGCTGGAAGCGATCAGCGAATAC

GGTGGCACCATCAGCGGCGGCCCGGATTTCGCCTACCGCCTGTGCAGCGAACGGGTCAGC

GAGTCGGCCCTGGAGCGCCTCGACCTCAGCGGCTGGCGCGTGGCCTATTCGGGGTCCGAG

CCGATCCGCCTGGACACCCTGGAGCGTTTCGCCGAGAAGTTCAGCGCCTGCGGTTTCAGC

CATGAAAACTTCCTCGCCTCCTACGGCCTGGCCGAAGCCACCCTGTTCGTGGCCGGTGGC

GTGCGCGGCCAGGGCATCCCGGCCCTGCACCTGGACGACCAGGCCCTGGCGCAGAACCGC

GCCGAGCCGGGGCAGGGCAGCGCGATGATGAGCTGCGGCTTCAGCCAGCCGGAACACGCC

GTGCTGGTGATGGATCCGCAGCAACTGGGCGAATTGCCTGACAACCGTATTGGCGAAGTC

TGGGCCGCCGGGGCGAGCATTGCCCATGGCTACTGGCGCAATCCTGAAGCCACCGCCAAG

ACCTTCGTCCAGCATGCCGGTCGTACCTGGTTGCGCACCGGTGACCTGGGCTTTATCCGC

GATGGCGAGCTGTTCATCACCGGGCGCCTGAAGGACCTGCTGATCGTGCGCGGGCACAAC

CTCTACCCCCAGGACATCGAAAAGACCATCGAGCGCGAGGTGGAAGTGGTGCGCAAGGGC

CGGGTGGCGGCGTTTGCGGTCACCGAGCAGGGCCAGGAAGGCATCGGCATCGCCGCGGAA

ATCAGCCGCAGCGTGCAGAAGATCCTGCCCCCCGAGGCGCTGATCAAGGCCATCCGCCAG

GCGGTGGCCGAAGCCTGCCAGGAAGCGCCGAGCGTGGTGGTGCTGCTCAACCCCGGGGCC

CTGCCGAAAACCTCCAGCGGCAAGTTGCAGCGTTCCGCCTGCCGCACGCGGTTGGCCGAT

GGCAGCCTGGATTACTACGCGCTGTTCCCGCAGGCCGGCAGCGCAACGGCTGACGCCGCA

GAAAGCGCCGGTGACGCGCTGCAAGCCCTGATCGGGCGCATCTGGCAGGAGCAACTGCAA

TGCGCTTCAGTGGCGGCCGACGATCACTTTTTCCTCCTGGGGGGCAACTCGATTGCCGCT

ACCCAGGTGATCGCCCGCCTGCGTGAAGAGCTGGGGCTGGAACTGGGCTTGCGCCTGCTG

TTCGAGGCTCCGACCCTGGGTGCCTTCAGCGCTGCCGTGGCGCGCCAGCAGCAGGACGGC

GGCGTGGCCCAGGGCAGTATCAATGCCCTGTCGCGCCAGCAGCCGCTGCCCCAATCCCTG

GCGCAGAACCGCCTGTGGATCACCTGGCAACTGGACCCGCAGAGCAGCGCCTACAACATT

CCCGGCGGCCTGCGCTTGCGTGGCGAGCTGGATGAAGAGGCCCTGCGCGCCAGCTTCCAG

CAACTGATCCAGCGCCACGAAGCGCTGCGCACGCGCTTCTTCGAGCGTGACGGCCAGGCC

CTGCAACAGGTGGATGCCGCCGGCGAATTCAACCTGCAGGTGATCGATATCAGCGACCTG

CCACCGGCCGAGCGTGAAGCCCGGGCGCGGCAGATCCGCGAAGACGAGGCCCGGACCCAG

TTCGATCTGGAGAAGGGGCCGCTGTACTGGGCGACCCTGGTGCGCCTGGATGACGAAGAA

CACCAACTGCTGCTGACCCTGCACCACATCATCGCCGACGGCTGGTCGCTGAATGTGCTG

ATCGACGAGTTCTCGCGGCTCTATGCCGCCGCGGCCCAGGGCCAGAGCCTGGAGCTGGCG

CCCCTGGCCCTGCAATACGCCGACTACGGCAGCTGGCAGCGCCAGTGGCTGGCCCAGGGC

GAAGGCCAGCGCCAGCTGGACTACTGGAAACAGCAGTTGGCGGACGAAGCCCCGGTGTTG

AACCTCGCCACCGACCACCCGCGCTCGGCCCAGGTCCGCCACAGCGCCGCGCGCCATGAC

CTGCGCCTGAGCGCGAGCCTCAGTGAAGCCGTGCGCCAGACCGCCCAGGCTCGGCAGGCC

ACGCCGTTCATGCTGTTGCTGGCGGCGTTCCAGACCCTGCTGCACCGCTACAGCGGGCAG

AGCGATATCCGCATCGGTGTGCCCAACGCCAACCGCCCGCGCCTGGAAACCCAGGGCCTG

CTGGGCTTTTTCATCAACACCCAGGTATTGCGTGGGCAGTTGGATTCGCGCCAGTCCTTC

GCCGACCTGCTGCAGCAGACCCGCCGCACCACCCTGGAGGCCCAGGCCCACCAGGACCTG

CCTTTCGAACAATTGCTCGAAGCCTTCCCCGAAGCCCGCGAGCAGGGCCTGTTCCAGGTC

ATGTTCAACCACCAGCAGCGCGACCTCAGTGCCTTGCGCCGCCTGCCGGGGCTGCTGGCC

GAAGAGCTGCCGTGGCACAGCCGCGAAGCCAAGTTCGACCTGCAACTGCACAGCGAAGAG

GACCGCAACGGCCGCCTGAGCCTGGCCTTCGACTACGCCGACGAGCTGTTCGACGCGGCC

AGCATCGAGCGTCTGGCCGGGCATTTCATCAGCCTGCTGGAGCATGCCTGCCTGCAACCG

GACTGCGCCCTGGGCGACTTGCCGTTGCTCGGCAACGCCGAAGCGGCGCAACTGCAGCAA

TGGAGCGTGGCCCCCTGCGCGCCGGCCGAGCAGTGGCTGCCGGAGCGCCTGCACGAACAA

CTGCGCCAGAGCCCGGAACGCACCGCGCTGGTCTGGGACGGCGGGCAACTGGGCTTTGCC

GAACTGCATGCCCAGGCCAACCGCCTGGCCCACTACCTGCGGGACAAGGGGGTCGGCCCG

GACGTGTGCGTGGCCATCGCCGCCGAGCGTTCGCCGCAACTGCTGATCGGCCTTCTGGCA

ATCATCAAGGCCGGCGGCGCCTACGTACCCCTGGACCCGGACTACCCTGCCGAACGCCTG

GCCTACATGCTCGAAGACAGCGGCGTGGGCCTGCTGCTGACCCAGACCCATCTGCTGGGC

CGGATGCCCGAAGCCCCGGGTGTCAGCGCCATCGCCATGGACAGCCTCAAGCTCGACAGC

TGGCCGAGCCACGCGCCGGGCCTGCACCTGCACGGCGAACACCTGGCCTATGTGATCTAC

ACCTCCGGTTCCACCGGCCAGCCCAAGGGCGTCGGCAACACCCATCGCGCGCTGATGGAG

CGCCTGCAATGGATGCAGGACAGCTACCAGCTGCAAGCCGACGATGTGCTGATGCAGAAG

GCTCCCATCAGCTTCGACGTGTCGGTGTGGGAGTGCTTCTGGCCGCTGATCACCGGCTGC

CGCCTGCTGATCGCCGCCCCCGGCGAGCACCGCGATCCCCATCGCATCGCCCAGCTGGTG

CAGGAGTACGGCGTCACCACGCTGCACTTCGTGCCGCCGCTGTTGCAGTTGTTCGTCGAT

GAGCCGCTGAGCGCCGAATGCCACAGCCTGCGCCGGGTGTTCTCCGGTGGCGAAGCCCTG

CCGGCCGAGCTGCGCAATCGCCTGTTGCAGCAGTTGCCCAATGCGCAACTGCACAACCGC

TATGGCCCGACCGAAACCGCGATCAACGTCACCCATTGGCATTGTTCAGTCGATGACGGC

GAGCGTTCGCCCATTGGCCGGCCCCTGGGCAACGTGGTGTGCCGGGTGCTGGACGCGGAC

CTCAATCCGTTGCCGGCGGGAGTTCCGGGTGAGCTGTGCATCAGCGGCCAGGGCCTGGCC

CGGGGTTACCTGGGACGCCCGGCGCTGACCGCAGAACGCTTTGTGGTCGACCCCCTGAGT

GAGGAGGGCGCGCGCCTGTACCGCACCGGTGACCGCGTACGCTGGTGCGCCGACGGCGTG

CTGGAATACCTCGGTCGCCTCGACCAGCAGGTCAAGCTGCGGGGCTTTCGGGTCGAGCCG

CAGGAAATCGAAGCGCGGCTACTGGCCCAGGACGGCGTGGCCCAGGCCGCGGTGCTGGTG

CGCGACACCCTGGCCGGCCCGCAGTTGATCGGCTACTACACGCCCAATGCGCACGATGAA

GATGAACAGCAGCAAAGCGCCCGGCTGAAAACCGCCCTGGCCGCCGAGCTGCCGGAATAC

ATGGTGCCGGCGCAACTGCTGCGGCTGGACGCCATGCCCCTGAGCCCCAGTGGCAAGCTC

GACCGCCGGGCCCTGCCGGAGCCGCAATGGCAGGTGCGCGAGCACGTCGAACCGAGCACC

GAACTGGAACAGCAGATCGCGGCTATATGGCGCGAAGTGCTGGGCCAGCCACGCATCGGC

CTCAAGGACGATTTCTTCGCCCTGGGCGGGCATTCGCTGCTGGCCACCCAGATCATTTCC

CGCACCCGCCAGGCCTGCGACGTCGAGCTGCCACTGCGGGCGCTGTTCGAAGCCAGCGAA

CTGGGGGCCTTTGCCGAGCAGGTGCTGCTGATCCAGGAGTCCGGCGCGCGCAACCAGCAG

CCGCCGATTGCCCGGGTAGACCGCAGCCAGCCGGTGCCGCTGTCCTATTCCCAGCAGCGC

ATGTGGTTCCTCTGGCAGATGGAGCCCGACAGCCCGGCCTACAACGTCGGTGGCATGGCG

CGGCTGTCTGGCGTGCTCGACGTGGGCCGCTTCGAGGCCGCGCTGCAAGCGCTGATCCTG

CGTCACGAAACCCTGCGCACCACCTTCCCCAGCGTCAATGGCGTGGCCCATCAGCAGGTG

CATGCCGACACCGGCCTGCGCATGGCCTGGAAGGACTTCTCGGCCCTGGCCCCGGATGCG

CGTCAGCAGCGCCTGCAACAATTGGCGGACAGCGAAGCGCATCAACCCTTCGACCTGGAG

ACCGGCCCCTTGCTGCGCGCCTGCCTGGTCAAGGCCGGCGAGCAGGAGCATTACTTCGTC

CTGACCCTGCACCACATCGTCACCGAAGGCTGGGCCATGGACATCTTCGCCCGCGAGCTG

GGCGCGCTGTACGAAGCCTTCCTCGACGACCGCGAATCGCCCCTGGAACCCTTGCCGGTG

CAGTACCTGGACTACAGCGTGTGGCAGCGCCAGTGGCTGGAATCCGGCGAGCGCCAGCGC

CAGCTGGACTACTGGACCGGGCAACTGGGCCGCGAACACCCGCTGCTGGAGCTGCCCAGC

GACCGGCCACGCCCGGCGGTGCAAAGCCACCAGGGCGAACTCTATCGCTTCGATTTGAGC

GACGAGCTGGCGGCCCGGGTGCGGGCCTTCAATGCCGAGCATGGCCTGACCCTGTTCATG

ACCATGACCGCCGCGTTGTCGCTGCTGCTCTATCGCTACAGCGGCCAGAGCGACCTGCGC

ATCGGCGCGCCGGTGGCCAACCGCATCCGCCCGGAAAGCGAAGGGCTGATCGGCGCCTTC

CTCAATACCCAGGTGCTGCGTTGCCAGCTCGACGGGCAGATGAGCGTCGGCCAGTTGCTG

GAGCAGGTACGCCACACCGTGATCGAGGGCCAGTCGCACCAGGACCTGCCGTTCGATCAT

CTGGTGGAAGCCTTGCAACCGCCGCGCAGCACCGCCTACAACCCGCTATTCCAGGTGATG

TGCAACGTCCAGCGCTGGGAATTCCAGCAAAGCCGGCAACTGGCGGGCATGACCGTGGAA

TACCTGGTCAACGATGCCCGGGCCACCAAGTTCGACCTCAACCTGGAAGTCACCGACCTG

GACCAGCGCCTGGGCTGCTGCCTGACCTACAGCACCGACCTGTTCGACGAGCCGCGCATT

GCGCGCATGGCCGGCCACTGGCGCAATCTGCTGGAAGCCTTGCTCAGCGACCCGCAACGG

CGCCTTTGCGAATTGCCACTGCTGCAGGCCGATGAACAGCAGCACCTGCTGGACAGTTTG

AGCGTCGAGCCCGGCGAGCAACGTCTGGACCAGTGCATCCACCACCTGTTCAGCGAACAG

GCCCTGGCCCGCAAGGATGCCCCGGCCCTGACCTTCGCCGGGCAGACCCTGAGCTACAGC

GAGCTGGACAGCCGCGCCAACCGCCTGGCCTGGATGCTTCGTGAGCGTGGCGTGGGCCCG

CAGGTGCGAGTCGGCCTGGCCCTGGAGCGTTCCCTGGAAATGGTCGTCGGCCTGCTGGCG

ATCCTCAAGGCCGGTGGCGCCTACGTGCCCCTGGACCCGGAATACCCCCTGGACCGCCTG

CACTACATGATCGAAGACAGCGGCATCGGCCTGCTGCTCAGCGATGCGCGGATGTTCGCC

GCCCTGGGCGAGCTGCCGGCCGGAGTCGGCCGCTGGTGCCTGGAAGAGGACGGTGCGCTG

CTGGCCGACTACCCGGCGGGCGAATTGCCCTTCATCAGCCTGCCCCAGCATCAGGCGTAC

CTGATCTACACCTCCGGCTCCACCGGCCAGCCCAAGGGCGTGGTGGTGTCCCACGGGGAA

ATCGCCATGCACTGCCAGGCGGTGATCCGCCGCTTCGGCATGCGCGCGGACGACTGCGAG

CTGCACTTCTATTCCATCAACTTCGACGCCGCCACCGAGCGCTTGCTGGTGCCGCTGCTC

AGCGGCGCCCAGGTGGTACTGCGGGCCCAGGGCCAGTGGGACGCCGAAGAGATCTGCCAA

CTAATCCGTCAGCACCGCATCAGCATCCTCGGTTTCACCCCCAGCTACGGCAGCCAGTTG

GCCCAGCATCTGGCGACCCAGCAGCAGACCCTGCCGGTGCGCATGTGCATCACCGGCGGC

GAGGCCCTGACCGGCGAACACCTGCAGCGCATCCGTGCGGCCTTCCAGCCGAACCTGTTC

TTCAACGCCTACGGCCCCACCGAAACCGTGGTCATGCCCCTGGCCAGCCTGGCGCCGCAG

CAGTTGGCCGAAGGCGTGGCCAGCGTTCCCATCGGCAGCATCGTCGGGGCCCGGGTGGCT

TACATTCTCGATGCCGATCTGGCGCTGGTGCCCCAAGGGGCGACCGGTGAGCTGTATGTC

GGCGGCGCCGGTCTGGCCCAGGGTTATCACCGTCGTCCGGGGATGAGTGCCGAGCGTTTT

GTCGCCGATCCGTTCGCCACCGATGGCGGGCGCCTGTACCGCACCGGGGACCTGGTGCGC

CAGTGCGCCGACGGCCAGGTGGAATACATCGGCCGGGTGGACCATCAGGTGAAGATTCGC

GGTTTCCGCATCGAGCTGGGGGAAATCGAAACCCGCCTGCTGGATCATCCCGCGGTGCGT

GAAGCCGTGGTACTGGCCTTGGATACCCCGGCCGGCAAGCAGCTGGCTGGTTACCTGGTG

ACCGAGGTGGCCGAGCACAACGAGGTGCAGCAGGCCAACCTGCGTGAAGCCCTCAAGCAG

CAGCTGAAAACCCAGCTGCCGGACTACATGGTGCCGACCTACCTGATCCTCCTTGCCAGC

ATGCCGCTGACCGCCAACGGCAAGCTCGATCGCCGAGCCCTACCGATGCCCGATCCGGAG

CTCAACCGCCAGCAGTACGTGGCGCCGAGCAACGAGCTGGAACAGACCCTGGCCAGGGTC

TGGGGCGAAGTGCTGAACGTGCAGCAAGTGGGCCTCAACGACAACTTCTTCGAACTGGGG

GGCGACTCGATCCTGTCGATCCAGGTGGTCAGCCGCGCCCGCCAGTTGGGGATTCACTTC

ACCCCGCGGGACCTGTTCCAGCACCAGACGGTGCAAACCCTGGCGCGGGTTGCCAGCCAC

ACTCAGCGCGTCAGCGCCGAGCAGGGCCAGCTCAGTGGCGAAGCGCCGCTGACGCCGATC

CAGCACTGGTTCTTCGACAGCCGCATTCCCCAGCCGCAGCACTGGAACCAGGCCTTGTTG

CTGGAACCGCTGGGCACCCTGGATGCGTCGTTGCTGGAGCAAGCCCTGCTGGCGGTGCTG

GAGCAACACGACGCCCTGCGCCTGCGCTTCAACCAGGCGGGCGGGCAGTGGCGCGCCGAG

TACCTGCCGTTGTCCGATGCTCCCTTGTTGTGGCAGGTGCGGGTGCCGTCCATGGCCACC

TGCGAGGCACTGTTCGCCGATGCCCAGCGCAGCCTCGACCTGGAACACGGGCCGCTGCTG

CGGGCGGTGCTGGTGGACGGCCCTGAAGGCGAGCAACGCCTGTTGCTGGCCATCCACCAC

CTGGTGGTGGACGGTGTGTCCTGGCGCGTGCTGCTGGAAGATTTGCAAAACGCCTATCGT

CAGTTGCAAGCCGGAAAGGCCCTGAACCTGCCGGCCAAGACCAGTGCCCTGCGGGACTGG

TCGAGCCGCCTGCTGGCCTATGCCGGCAGCGAATCCCTGCGTGAAGAGCTGAGCTGGTGG

CAGCAGCAATTGGCCGGCCCGGCCGCGCAACTGCCGGGGCTCAAGGCCACTGGCAGCCAG

CAGCATCAACAGGCTCAAAGCCACAGCGTGACCCTGGATGCCGAGCGCACCCGTCAGTTG

CTGCAACAGGCTCCAGCGGCCTACCGCACCCAGGTCAACGACCTGCTGCTGACCGCCCTG

GCCCGGGTGCTGTGTCGCTGGAGCGGGCAGCCTTCGGCCCTGGTGCAATTGGAAGGCCAC

GGCCGCGAGGCGCTGTTCGACGAGATCGACCTGACCCGCACCGTCGGCTGGTTCACCAGC

GCCTATCCGTTGCGCCTGACCCCGTTGCAGGTGGAAGAAGCGGCCGGGCAGGGCGCCTCG

ATCAAGGCCATCAAGGAACAACTGCGGGCCGTGCCGCACAAGGGCCTGGGTTACGGCGTG

CTGCGCTACCTGGCGGACGACGCCTGCCGCGAGGCCCTGGCGGCGTTGCCGCTGGCGCCG

GTGACCTTCAACTACCTGGGGCAGTTCGACCAGAGCTTCGGCGCCGACGCACTGCTGCGT

CCGCTGGATGAATCCGTCGGCCCGGCCCACGCACCGGAGGCACCGTTGCCCAACGAACTG

AGCATCGACAGCCAGGTCTATGGCGGCGAGCTGGTGCTGCGCTGGACCTACAGCAGCGAA

CGTTTCGACGCCGAGCTGATCGGCGAGCTGGCGGACGCCTACCTGGGCGAGCTGCACAGC

CTGATCGGCCATTGCCTGAAGGACGATGCCGGTGGCCTGACGCCGTCGGACTTCCCCCTG

GCGCGCCTGACCCAGGCCCAGCTAGATGGCCTGCCGGTACCGGCGGCGCAGATCGAAGAC

GTCTACCCGCTGACCCCGATGCAGGAGGGCATGCTGTTGCACACCCTGCTGGAGCCGGGC

ACCGGCCTGTATTACATGCAGGACCGCTACCGCATCAACAGCGAACTGGACCCGGAGCGT

TTCGCCCAGGCCTGGCAGGCGGTGGTGGCGCGCCACGAAGCCTTGCGCGCGTCTTTTTGC

TGGAACGTCGGCGAAGACATGCTGCAGGTTATCCACAAGCCGGGCCGCACCCCGATCGAG

TTTCTCGACTGGAGCGCGGTGCCCGAGGCCGAGCAGGAAGCCAAGCTGCAAGCCCTGCAC

AAACAGGAGCGTGAGGCCGGCTTCGATCTGCTCAACCAGGCGCCGTTCCACCTGCGGCTG

ATCCGCGTTGGTGCGGCCCGTTACTGGTTCATGATGAGCAACCACCACATCCTCATCGAT

GCCTGGTGCCGCTCGCTGCTGATGAACGACTTCTTCGAGATCTACACCGCCCTCGGTGAA

GGTCGCGAGGCGCAACTGGCGGTGCCACCGCGTTACCGCGACTACATCGGCTGGCTGCAA

CACCAGAGCCTGTCCGAGGCGCGGCAGTGGTGGCGGCAGAACCTTGAGGGCTTTGAACGC

ACCACGCCGATCCCCAGCGACCGGCCGTTCCTGCGCGAACACGCCGGCGACAGCGGCGGC

ATGACAGTCGGCGACTGCTACACCCGCCTGGATGCCCGGGACGGCGCGCAACTGCGGGAA

CTGGCGCAACAGCACCAGCTCACCGTCAACACCTTCGCCCAGGCGGCCTGGGCCCTGGTG

CTGCGGCGCATGAGCGGCGATCGCGATGTATTGTTCGGGGTCACCGTGGCCGGGCGCCCG

GTGGAAATGCCGGAGATGCAACGCACCGTCGGCCTGTTCATCAACAGCATCGCCCTGCGG

GTCAAGCTGCCCCAGGACGGTGAGCGCTGCAGCGTGCGCCAGTGGCTCAGCGGCTTGCTC

GACAGCAACATGCAGCTGCGCGAATACGAGTACCTGCCGCTGGTGGCGATCCAGGAAACC

AGCGAACTGCCCAAGGGCCAGCCGCTGTTCGACAGCCTGTTCGTGTTCGAGAACGCGCCG

GTGGAAGTCTCGGTGCTGGACCGTGCCCAGAGCCTGAACGCCAGTTCGGATTCCGGCCGT

ACCCACACCAACTTCCCGATCACCGCGGTGTGCTACCCGGGGGACGACCTGGGCCTGCAC

CTGTCCTACGACCAGCGCTACTTCGAGCAGGCGACCATCGAACGCATGCTCGGCGATTTC

AAGCGCCTGCTGCTGGCCCTGATGCAGGGCTTTCATGGCGACATGGCCGAACTGCCGCTG

CTGGGTGAAGAGGAGCAGGACTTCCTGCTGGCCGGCTGCAACCAGAGCGAGCATGAGTAC

CCGCTGGAGCGCAGCTACGTGGAACTGTTCGAGGCCCAGGTGGCGGCCCATCCGCAACGC

ATCGCCGCCAGTTGCATGGACCGGCGCTACAGCTATGCCGAGTTGAACCGCTGCAGCAAC

CGCCTGGGCCACGCCCTGGTTGCCAACGGCGTAGGCTTTGATCAGCCGGTGGCCCTGCTG

GCCGAGCGCGGCCTGGAACTCTTGGGCATGATCATCGGCAGCTTCAAGGCCGGGGCCGGC

TACCTGCCCCTGGACCCGGGCCTGCCGGGCCAGCGCCTGGGCCGTATCATCGAGCTCAGC

CGCACGCCGATCCTGGTGTGCAGCGCGGCCTGCCGCGAACAGGCCCAGGCCCTGCTGGAC

GAGTTCGGCTGTGCCGGGCGTCCGCGGCTGCTGGTCTGGGAAGAGCTGCAAGCCGCCGGG

CATGCCGAGCACAACCCGGGCCGCTACAGCGCGCCGGACAACCTGGCCTACGTGATCTAC

ACCTCCGGTTCCACCGGCCTGCCCAAGGGCGTGATGGTGGAGCAGCGCGGCATGCTCAAC

AACCAGTTGAGCAAGGTGCCGTACCTGCAACTGAGTGAGGCCGACGTGATCGCCCAGACC

GCGTCCCAGAGCTTCGACATTTCGGTCTGGCAGTTCCTTGCCGCACCGCTGTTCGGCGCT

CGGGTGGACATCGTGCCCAACACCATCGCCCACGATCCCCAGGGCCTGTTGGCTCACGTC

CAGGAGCAGGGCATCACGGTGCTGGAGAGCGTGCCGTCGCTGATCCAGGGCATGCTCGCC

CAGGACGCCATCGCCCTGGATGGCCTGCGCTGGATGCTACCCACCGGCGAGGCCATGCCA

CCGGAGTTGGCGCACCAATGGCTGCTGCGTTATCCACAGGTCGGCCTGGTGAACGCCTAC

GGTCCGGCGGAATGCTCCGATGACGTGGCCTTCTTCCGTGTGGACATGGCTTCGACCCGG

GGCGCCTACCTGCCCATCGGCACGCCCACCGACAACAACCAGTTGTACCTGATGGACGAA

GCCCTGGAGCTGGTGCCCCTGGGAGCGGTGGGTGAATTGTGCGTGGCTGGCACCGGTGTT

GGCCGTGGCTATGTCAGCGACCCGTTGCGTACCGCGCTGGCCTTCGTGCCCCATCCGTTC

GGTGCTGCGGGCGAACGCCTGTACCGCACCGGCGACCTGGCACGCCGGCGCAGCGACGGC

GTACTGGAATACGTTGGCCGGATCGACCATCAGGTGAAGATCCGTGGCTATCGCATCGAA

CTGGGGGAAATCGAAGCGCGCCTGCACGAACAGCCGGAACTGCGCGATGCCGCCGTGGGC

GTGCAGGAGGGCGTCAACGGCAAGCACCTGGTTGGGTACCTGGTGGCCAGTGACACGAAC

CTGAGCCCGAGCGAGTGCCTGGAGCGGATCAAGCCGCGGCTGCGCGCCGAACTGCCGGAA

TACATGGTGCCGCTGCATTGGCTGTGGCTTGCGCGCCTGCCCCTCAACGCCAACGGCAAG

CTCGATCGCAAGGCCCTGCCGGCCCTGGAGATCGGCCAGCTGCAGAGCCAGGACTACCTG

GCCCCGGGCAATGAACTGGAGCAAACCCTGGCGGACATCTGGGCCGAGGTGCTCAAGGTC

GAACGGGTGGGCGTGCGCGACAACTTCTTCGAGTTGGGCGGGCATTCCCTGCTGGCGACC

CAAATCGCCTCACGGGTGCAGAAAACCCTGCAACGCAACGTGCCGCTGCGGGCCATGTTC

GAATGCAGCACGGTGCAGGAGCTGGCCGCCTACATCGATGGCCTGGGGGCCAATGAAATC

AGCGAGGAGAAGGTCGACCGGCTGAGTGATCTGATGGCCGAGCTGGAGGGGCTGTGA

>pvdS fig|380021.185.peg.4158 Sigma factor PvdS, controling pyoverdin biosynthesis

ATGACGGAACAAGTATCCACAAGCAGGTGCGATTCACCGTTACTCCAGGCTTTTGTCGAC

AACCGTCTGATCCTGGTGAAGATTGCAGCCCGCATTACCGGCTGCCGATCCCGGGCTGAA

GACGTGGTTCAAGATGCGTTTTTCCGCCTGCAGTCGGCGCCGCAGATCACCTCCTCGTTC

AAGGCCCAGCTCAGTTACCTGTTCCAGATCGTGCGCAACCTGGCCATCGACCACTACCGC

AAGCAGGCGCTGGAGCAGAAGTACTCGGGTACTGAAGAGGAAGGCTTGAATGTGGTTATT

CATGGCGCTTCACCTGAAACCTCCCACATGAACTTTTCCACCCTGGAAAACATCGCCGAA

GCCCTGACCGAGCTGCCCAGCCGCACGCGCTACGCCTTCGAGATGTACCGCCTGCATGGC

GTCCCGCAAAAGGACATCGCCAAGGAGCTGGGGGTATCGCCGACCCTGGTCAACTTCATG

ATTCGCGATGCCCTGGTGCATTGCCGCAAAGTCTCGGGCAACCGCCCCGATACCTTCGCC

CGTCGCTGA

>pvdQ fig|380021.185.peg.942 Acyl-homoserine lactone acylase PvdQ (EC 3.5.1.-), quorum-quenching

GTGATCATTTCCAGGCAGTTACCGAGTTTTTGCCTTGCAGCCCTGTTTCTAAGCTTCAGC

GGTGGCGCCCATGCCTTGGCGCAGCCCGAGCAGACCCGCGCCGAGATTCGCCGCACCAGT

TTTGGTGTGCCGCACATCCGTGCCGACGACGAGCGCGGCCTGGGCTACGGCATCGGCTAC

GCCTACGCCCAGGACAACCTGTGCCTGATGGCCAATGAGGTGCTGACGGTCAACGCCCAG

CGCTCGCAATACTTCGGCGCCGAGGGCCAGACCCTGGAACAGCGCGACAACCTCAGCAGC

GACCTGTTCTTCAGCTGGCTCAACACTCCCCAGGCCGTGGCGGCCTTCTGGCAGGCCCAG

ACCCCGGCGATGCGCGAGCGCATGCAGGGTTACGTGGAGGGCTACAACCGCCAGCTGGCG

GAGCGCCAGGCCCAGGGCCTGCCCGAGCAATGCCGGGGTGACTGGGTGCGGCCGCTGGCC

ACCTCGGACCTGGTAAAACTGACCCGACGCCTGTTGGTGGAAGGCGGTGCCGGGCAGTTC

GCCGAGGCCCTGGCCGGGGCCACGCCACCTGGAGCTACGGCCCAGGCCGGGCTGCCGGCC

GAGCACTGGCAACTGGCGGCGGCACGCCAGCAGCGTTTCGCCCTGGACCGTGGCAGCAAC

GCGGTGGCCATCGGTAGCGAGCGTTCGTTCAACGGCCGTGGGCTGCTGCTGGCCAACCCG

CATTTCCCCTGGGTCGGCGGCATGCGCTTCTACCAGATGCACCTGACCATTCCCGGACAA

CTGGATGTGATGGGCGCGGCCTTGCCCGGCCTGCCGCTGATCAATATCGGCTTCAACCAG

CACCTGGCCTGGACCCACACCGTGGACGCCTCCAAGCACTTCACCCTGTACCGCTTGCAA

CTGGACCCCAAGGACCCGACCCGCTACCTGCTGGACGGCCGTTCGCTGCCCCTGGAGCGC

CAGACCCTGACCGTGCAGAGCAAGGGCCCGGACGGCCAGTTGCAGCCGCGTACGCGCACG

CTCTACAGCTCGGTCTTCGGCCCGATCGTGCAGTGGCCGGGGGAACTGGACTGGGATCAT

CAATATGCCTACAGCCTGCGCGACGCCAACCTGGACAACAGCCGGGTGCTGGCCCAGTGG

TACGCGATGAACCAGGCCAGCAGCGTGGCCGGCCTGCAAGACAGCGTGCACCAGTTGCAG

GGCATTCCCTGGGTCAACACCCTGGCGGTGGATGACCAGGGCCGGGCGCTGTACATGAAC

CAGTCGGTGGTGCCCAACGTCACCCAGGCCAAGCTGGCGCAGTGCAGTGATCCGCGGGCC

GGCACCCGGGTCATCGTTCTGGATGGCTCGCGCAGTGCCTGTGCCTGGGATATCGACCCG

GCAGCGGCGCAACCGGGGATCTTCGCCGCCAGCCAGTTGCCGCAACTGACGCGCAACGAC

TACCTGCAGCATTCCAACGATTCGGCCTGGATGGTCAACCCGGCGGCGCCGCTGCAAGGC

TTTTCGCCGGTGATCAGCGAGCAGGACGTGCCGCTGAAGATGCGCACGCGCTTTGCTCTG

GATCGCCTGAGCCGGATGCACAAGGCCCAGGTCAGCGACCTGCAACACCTGGTCACGGAC

GATCAGGTGTACCTGGCCGGCCAGGTGATGCCCGATCTGCTGCAGTTCTGTGAGCAGGAC

CTGGGGGCGGATGCCCAGCGCCTGGGGCCGGTGTGCGCCAGCCTGAAAGCCTGGGACCGC

AGCGCGGGGTTGCAGGCCGGCCTGGGCTTCGTGCACTTCCAGGGCATCATGCAGCCGCTG

TTGCAGGACCCCAGCGTGTGGCGCGTGGCCTTTGACCCGAAAGATCCCCAGCACACCCCA

CGGGGCCTGGCCATTGGCCGGCCAGCGGTGGCCCGGGCACTGCGCGAGTCGATGCTGGCT

TCGGCGCAGCAGGTCGCCGAGGCCGGGCTGGGGAGTGACGTTCGTTGGGGCGATATCCAG

CAGGTCAGCCAGGGCGGCCAGCCGACCCCGGTGCCCGGCGGCCCTGAAAGCCTGGGGGTG

TACAACGCCATCCAGAGCGTGCCGGCGGCCGATGGCAAGCGTGAAGTGGTCAGCGGTACC

AGCTACCTGAACGTGGTCAGCTTCGACGAGCAGGGGCCGCGCGCCCTGGGGCTGCTGGCG

TTCTCCCTGTCCAGCGACCCGGCCTCAGCGCATTTTCGCGACCAGACGGCTGCTTTCGCC

CGCAACCAGTGGAGCGTGCTGCCCTTTACCGAAGCGCAGATCCGTGCCGATGGCCAGTAT

CAGTTGCAGGTGATCGAAGAGCCGCGCAAGGAGGCGGTACTGGCGCGCCAGTAA

>pvdE fig|380021.185.peg.2161 PvdE, pyoverdine ABC export system, fused ATPase and permease components

ATGACCGACCCCAAGCGCGGCGCCTTCAACGGTCTGCTCGCATTGCTCAGGCCCTTTCGC

ACCATCGTGGTGATCTCCGTTGCCCTGGGCATGGCCGGCGGCCTGGCCATCACCCTGTTG

CTGGCCACCATCAACAACGCCCTGCACTCGGCGACCGGCATGACCCAGGGCGTGGTCCTG

ACCTTCGCCGCCCTGTGCGTGCTGGCCCTGATCAGTTCCATCGTCTCGGACATCGGCACC

AACTATGTGGGACAACGGATCATTGCCGCCCTGCGCAAGGACCTGGGTGAAAAAGTACTC

TCGGCGCCCATCACCCAGATCGAGCGCTATCGCTCCCACCGCCTGATCCCGGTACTGACC

CACGACGTCGACACCATCAGCGACTTCTCTTTCGCCTTCACTCCCCTGGCCATCGCCACC

ACCGTCACCCTGGGCTGCCTGGGCTACCTGGCGTACCTCTCGGTGCCGATGTTCCTGATG

ATGGTGGTGGCCATCATCATCGGCACCAGTGTGCAGCTGGTGGCCGGCGGCAAGGGCATC

AAGGGCTTCGATGAGGCTCGCGACCATGAGGACGAGCTGCAGCGCTACTACAACGCGATC

GCCTCCGGTGCCAAGGAACTGCGTATCCACCGGCCCCGACGCTTTCGCATGAATACCCAG

CGCATCCAGAAAACCGCGGATCGCATCAGCGATATCCAGGTGCGCTCGGTGAACATCTAC

ATCCTGGCCAAGAGCTTCGGCTCGATGCTGTTCTTCGTGGTCATCGGCCTGGCCCTGGCC

ATGCAGGCCTACTACCCGAACCCGGACCCTGCGGTGATCACCGGTTTCGTGCTGGTGCTG

CTGTACATGAAGGGCCCGCTGGAACACGTGGTCGGCTACCTGCCCATCGTCGGCAAGGCC

AAGATCGCCTTCGCCCGGATCAGCGAACTGTCCGAACGCTTCTCTTCTCCCGAGCCGCAC

CTGCTGATGGACGACAGCGAAGCGCCGCAAGCGGTGGTGCACAGCCTGGAACTGCGCGAG

GTGCGCTACAGCCCGCCGCCGGTGGAAGGCAGCGAACCGTTCCACCTGGGCCCGATCAAC

CTGAACATCAAGCAGGGCGACATCGTCTTCATCGTCGGCGAGAACGGCTGCGGCAAGACC

ACCCTGATCAAGCTGTTGCTGGGCCTGTACCAGCCCCAGTCCGGGGAGATCCGTCTCAAT

GGCGAAGCGGTGACCGACCGGGCCCGGGATGACTACCGGCAGCTGTTCACCACGGTATTT

GCCGACTACTACCTGTTCGACGACCTGGTCCAGGGCAATGCCGGCAAGTCGCTGGACGTC

GCCACCCAGTACCTGAACCGCCTGGAAATCGCCCACAAGGTCAGCGTCAAGGATGGCGCC

TTCACCACCACCGACCTGTCCACCGGCCAGCGCAAGCGCCTGGCCCTGGTCAATGCCTGG

CTGGAAGAGCGCCCGGTGCTGGTGTTCGACGAATGGGCCGCCGACCAGGACCCGGCCTTC

CGGCGGATTTTCTACACCGAGCTGCTGCCCGATCTCAAGCGCCTGGGCAAGACCATCATC

GTGATCAGCCACGACGATCGCTACTTCGACATTGCCGACCAGTTGGTGCGCATGAAGGCC

GGACGAGTCCTGACTGAACTGCAACCGGCGTGA

>pvdO fig|380021.185.peg.2159 PvdO, pyoverdine responsive serine/threonine kinase (predicted by OlgaV)

ATGAAACCCATCGCCTGTTCCTCCCTGGCCACCCTGGCCGGTTGCCTGATCAGCCTCAAC

GCACACGCGGTAGAACCGCCCAAGCCCGGCAGCGTGTTCAAGGACTGCAAGAACTGCCCG

GAAATGGTGGTCCTGCCGGCCGGCAGCTTTGTCATGGGCACCCCGGAAGACGAAGTGGGC

CGCGAGCCCGATGAAGGCCCGCAGCACAGCGTGACCTTCAAGAACGCCTTCGCCATGAGC

CGCTTCCATGTCACTGCCGCCGAGCTTGATGCCTACATCCGCGAAACCGGTACCGTGATC

AAGGACGGCGACGACCGCCCCGGCCGCCTGTGCCAGGCCAGCAAGCCACGCTATGAACAG

GGGCCGCGCCAGCCGGCGGTCTGCGTCGACTACGCCGACGTGCAGGCCTATACCCAGTGG

CTGTCGAAGAAAACCGGCAAGCACTACCGCATGGTCAGCGAGGCGGAACGCGAATATGCC

GCACGCGCCGGCAGCACCGGTTCCTTTCCCTTCCCTTTCGACGAGGAAGGCCAGTACCAG

ATCACCAAGCACGCCAACACCTACGGCCCCAAGGACGGCTTCAGCTTCACCGCCCCGGTG

GGCAGCTATCCGCCCAATGCCTTCGGCATGTACGACATGCACGGCAATACCTATGAGTGG

GTCGCCGACTGCTGGCACCCCGACTACGTGGGCGCACCTGCCGATGGCCGGGCCTGGATG

GAAGAGTCGGAAGGGGTGTGCCTGGATGCCCAGATTCGCGGCAACGACTGGGGCGAGGCA

CCGGTATTCTCGCGTTCGGGCAACCGCAACAGCCGCAAGCGCGAAGTGCGCGGGGACTGG

CTGAGCTTCCGGGTCGTGCGTGAGCTGGAACAGCCCGGCGCCAGCCACAAGTAG

>pvdN fig|380021.185.peg.2158 Pyoverdin biosynthesis protein PvdN, putative aminotransferase, class V

ATGACCACGAGCCTTCCTGTGACCAACCGTCGGACATTCCTCAAACAAGCAGGCCTGTTG

GCCGCCGCCCTGCCCCTGGGCAACCTCGCCATCAGCCAGGCCCGGGCCGACAGTGCCACC

GCCAGTGCTTCGCAGTGGACCCAATTGCGCCAGTTGTTCGACCTCGACCCGGACTACGTG

CACCTGGCCAATTTCCTCATCACCTCGCACCCGAGGCCGGTGCGCGAGGCCATCGAAAAA

TACCGCGCAGTGCTCGACCGCAACCCGGCCATGGCCATGGACTACGACACCCAGTACACC

TGGAAGCGCGAGGCCCAGGTGCGCGAAAGTGTCGGCCGCTACCTGCAGATCAAGCCCGGC

CAGGTGGCCCTGACCGGCAGCACCACCGAAGGCCTGGCCCTGATGTATGGCGGCATCCAC

GTGCGCCCCGGACAGGAGATCCTGACCACGGTTCACGAGCACTACTCGACCCGCAACGCG

CTGAAGTACCGCACCCAGCGCGACGGCACCCAGGTGCGCACCATCGAACTGTTCAAGTCG

CCCCACCGGATGTCCACCGACGAAGTGCTGGGCAACATCGACCGCAACATCCGCGCCAAC

ACCCGCGTACTGGGCATGACCTGGGTGCAGTCGGGCAGCGGCGTGAAACTGCCGATCGGC

GAGATCGGCAAGCTGGTGGACCAGCACAACCGCAATCGCGATGAGCACGAGCGGATTCTC

TACTGCGTCGATGGTGTGCACGGGCTGGGGGTGGAGGACATCACCTTTGCCGACCTGAAC

TGCGACTTCTTCGTCGCCGGCACCCACAAATGGATGTTCGGCCCCAGGGGTACCGGGATC

TTCTGCTCGCGCTCGGAACAACTCGAACACCTGACGCCGATGGTGGCGACCTTCTCCGAA

AACCAGAACTTCGCCACCACCATGACCCCCGGTGGCTACCACGGCTTCGAGCACCGCTGG

GCTGTGGACCAGGCTTTCGACCTGCACATGCAGCTGGGCAAGGCCAACATCCAGTCGCGC

ATCCATGAACTCAACAGCTACCTCAAGCAGCGCCTGCAGGAGCACCCGGGCATCGAACTG

GTCACGCCCCTGAGCCCGGAGCACTCCGCCGGCTTCACCTTCTTCCGCGGCCAGAACCTG

GACACCGACGCCACCGCCGCCTGGCTGATCGAGAACCGCATCCTGGTGGACGCGGTGGAC

CGCGATGCCGGGCCGGTAGTGCGCATGGCCCCCGGCCTGCTCAACAGCGAGGCGGATATC

GACCGGACCATGGACCTGCTGGGCAAGCGCCTGCGCAGCAGCAAGACCGCCTGA

>pvdM fig|380021.185.peg.2157 Putative dipeptidase, pyoverdin biosynthesis PvdM

ATGATCAAAATATCTGGTAAGAAAACCCTCTATCTGAGCCTGCTGATACTTATCATCGCC

GCCCTCGGCGTCGGTGCCGGCGCGGCCTGGCACTACTACTGGAAGGATCGGGTGTCCTAC

CCCAACGAAATCGTCAAGCACGCCAACGACCTGCAAGAACGCATCATCTCCTTCGACAGC

CACATCACCGTGCCCCTGGATTTCGGCACCGAAGGCAGCGAAGCGGACAAGGACACCTCT

CGCCAGTTCGACCTGGTCAAGGCCGGTCGCGGCCGCATGTCCGGCGCCGCCCTGAGCATC

CTGGCCTGGCCGGAGATGTGGAACGGCGCCAACGCGCCGCACCGCCCCACCGCCGGCTTC

GTCGAGGAAGCCCGCCACCAGCGCGAGACCCGCTACCGGATCATCCAGGGCATGGTGCGT

GACTTCCCCAACCAGGCCGCCATCGCCTACACCCCCGCCGACCTGCGCCGCATTGCCTCC

GAAGGCAAGGTGGCGGTGGTGATCAGCCTGCTCAATGCCTACGCCATGGGCGACGACCTG

GACCAGCTGGACCAGTGGGCCGCACGGGGCATGCGCCTGTTCGGCTTCAGCTATGTGGGT

AACAACGACTGGGCCGACTCGTCGCGCCCGCTGCCCTTCTTCAATGACACCCGGGACGCC

CTCGGCGGCCTCTCGCCCGTCGGCAAGCAGGCGGTGCAGCGGCTCAATGACCTGGGGGTG

GTGATCGACGTCTCGCAGATGTCGAGCAAGGCCCTGGACGACGTTGCGAGCCTGACCCGG

GCACCGGTGGTGGCTTCCCACTCCGCCCCCAGGGCGCTGGTGGATATCCCGCGCAACCTC

AGCGACCAGGAAATGCAACAGATCAAGGCCACCGGCGGGGTGATCCAGGTCGTGGGCTTC

TCCACCTACCTGCGCCCCCTCAGCCAGCCGACCCTGGACAAGCTCAATGCCTTGCGCAAG

CGCTTCGACCTCGGGCCACTGCAAGGCCTGGAGAACGCACTGATGCCCGGCGACGCCGTG

ATCACCATCTGGCCGGAACAGCGCTTTGGCGAATACGCCAGCTCCCTGTACGGCATCCTC

GATGAGGAACCCAAGGCCAGCCTCAAGGACTATGTCGACGCCATCGATTACACGGTGAAG

AAAGTCGGCATCGACCACGTCGGCATCAGCTCGGACTTCAACGAGGGCGGTGGCGTCAAT

GGCTGGATGAGCGTGGCGGACAACCGCAACGTCACCGCCGAGCTGATCCAGCGTGGCTAC

AGCGATATCGAGATCGCCAAGCTCTGGGGCGAGAACTATCTGCGCGTCTGGGAACAGGTG

CAGAAACTGGCCAAGCCTGCAAAACCCGCCCCTCAACCCGCCATCGCCGGCTAA

>pvdP fig|380021.185.peg.2156 Pyoverdine biosynthesis related protein PvdP

GTGGCGTACTACACCCAGAAGGAGTTGCTGCGTCAGGATGTTCGCCGCAAGGAAGAGCAG

GAGATCACCCCCGGGGAAGCCAAGCTGGAGGTGGCCCTGGACCCTGGCGTGCGCCTTGCC

GATCACCTGCGGGGCATCTGGGATATCGAGTTTCTCGACGCCAACGGACCGCAATCCCTG

CCTCGTAAAGGTTGTGAGTTGTTACTGGATGCCGGCCCCACCGGGCGCGGCTTGCGTGGC

CTCCTGGGGCGTGCGCAGAGCCTGCGAGGCGACGGCCCGGTGGAGTTCATGGTGCAGGGC

GACCTGGCGTCGGTGGACGCCGCCAGGTTGCGCTGGCGCTTGTTTGCCGGTGGCCGCATC

GCCGGTGCCCCCAGCCATGAATGCCAGGTGGTGCTCGATGAGGTCTGGGCCAGTTGGGGC

AATGCCGGCAGCGGCACCCTCAGTGGCAGCCTGCAGTCGCTGCAGGCGTCGCCACTGGCG

CCGGTATCCGCGGCGCGTTTTGTGGCGCGCAAGCGAGTCTTTCCTGCCGCCCGGGACCAG

AGCGGGCTGAGCCCCGCCTTGCTGGCGTGGCTGGTATCGCCGCAGCACCGCCTGTTCCAT

CAGCTCTGGCACGCTTCGCGGGACCGTTGGCACACCCTGCCCAAGGACAAGCGCAACAGC

CTGCGGGGGCTCGGCTGGCAGCCGGGCCCGGTCAAGCATGAACGTGACGCCCGTGGGCGG

CGCAAGCACCGCAATGCCTCGGGCATCGATTTTCTGTTCATGCACCGGCACATGCTCCTG

CACGCCCGCTCGTTGCAGCCGGACCTGGTGTCCTGGCAGCAGTTGCCGCAGCCCTGTGCC

CGTCTTGAGCAGGACCGGCAGGCCTTTATCCGCTATTACGAAAACCACGACGGCTGTTCG

GTGCCACCGGCCTGGGTGGCCGACGATGATGAAGAGTTCACCCAATGGCTGCACGGGCTG

AAAAGTGATGCGGCGTTCTATGGCAACTTCCAGGTCTGGGAGTCGCAGTACCAGGACCCC

GAGTACCTCAGCCGCCTGACCCTGGGCCAGTTCGGGTCCGAGGTCGAGCTGGGCCTGCAC

GACTGGCTGCACATGCGCTGGGCGGATGTCGCCCGCGATCCTTCCAACGGCATGCCGGTG

ATGGAAGCGCGCCTGTCCTCGGACTTCGCCGGGCGCTGGTACCAGCCGCAGAATGACTTT

CTCGGCGATCCCTTCTCGTCCCACGTGCACCCCATGTTCTGGAAATTCCATGGCTGGATC

GATGACCGGATCGACGACTGGTTCCGGGCCCACGAGCGCTACCACCCCGGCGAAGTATTG

CGCCGCGAGGTCAATGGCGTGCCCTGGTTCGCCCCCGGGCGTTGGGTCGAGATCGACGAC

CCGTGGCTGGGGGCCTCGACCCATGGCTGCGGCCCCCTGGGCAATGCCCCGGGAGAAATG

TCCCTGGAGATGGATCAGGAGGTCATGAAGCTGGCCTTGCGCATCGCCCTGAGCCGCGAC

GAAGAGGTGCCGGACCTGCTCAAACGGGCACCGCGCCGGCCTTGGTATGCCCAGCACTTG

AAGCTCTGA

>pvdA fig|380021.185.peg.2149 L-ornithine 5-monooxygenase (EC 1.13.12.-), PvdA of pyoverdin biosynthesis @ Siderophore biosynthesis protein, monooxygenase

ATGACACAGGCAATTGCATCGGCCCACGTTCACGATTTGATCGGTATCGGTTTCGGCCCC

TCGAACCTGGCGCTGGCCATTGCGCTGGAGGAGCGCGGGCAGGAGCACGGTCCGCTGGAC

GCGTTGTTCCTCGACAAGCAGGCTGACTATCGCTGGCACGGCAACACCCTGGTAACCCAG

AGCGAACTGCAGATTTCCTTCCTCAAGGACCTGGTGACCCTGCGCAACCCCACCAGCCCC

TATTCCTTCGTCAACTACCTCAAGCACCACGGGCGCCTGGTGGACTTCATCAACCTGGGC

ACCTTCTATCCATGCCGCATGGAGTTCAACGACTACCTGCGCTGGGTGGCCGGGCATTTC

CAGGAACACAGCCGCTACGGCGAAGAAGTGCTGGCCATCGAGCCAGTGCTGCACAACCAG

CAGGTCGAGGCGTTGCGGGTGATTTCCCGGGATGCCCAGGGCAGCGAACTGGTGCGCACC

ACCCGTTCGGTGGTGGTCAGTGCCGGCGGCACGGCGCGGGTTCCGGAAACCTTCAAGGGG

CTCAAGGGCGATGCGCGGGTGTTCCACCATTCCCAGTACCTGGAACGCATGGCCAGCCAG

CCCTGTGTCAGTGGCAAACCGATGAACATCGCCATCATCGGCGGCGGGCAGAGCGCGGCA

GAAGCCTTCATCGACCTCAACGACAGTTTCCCTTCGGTGCAGGCCGACATCATCATGCGC

GGTTCGGCACTCAAGCCGGCGGATGACAGCCCGTTCGTCAACGAAGTGTTCTCCCCGGCC

TTCACCGACCTGGTGTTCCAGCAGACTGGCAGCGAGCGCGAGCGCCTGGTCAGCGAATAC

CAGAACACCAACTACTCGGTGGTGGACATCGACCTGATCGAGCGCATCTACGGGATTTTC

TACCGGCAGAAGGTTTCCGGCATCGCGCGCCACACCTTCCGCACCATGACCACGGTGGAG

AAGGCCACCGCTACCGACCTGGGCATCGAACTGATCCTGCGCAACAGTGCCAGCGGCGAG

CGCGAAGTACGTCACTACGATGCGGTGGTGCTGGCCACCGGCTACGAGCGGCAGATGCAT

CGTCAACTGCTGGCGCCGCTGCAGGCCTACCTGGGCGACTTCGAGGTGGACCGCAATTAC

CGCCTGGTGACCGACGAACGCTGCAAGGCCGGCGTCTACATGCAGGGCTTCAGCCAGGCG

AGCCATGGCCTGAGCGACACCTTGCTGTCGGTGCTGCCGATCCGTGCCCAGGAGATTGCC

GATTCGCTCTACGAACACGGCAAGAGCCGCGGTCACAGCCGTTCGGTGCGTGACATGCTG

CTGGCCACCGCCAGCTGA

>dhbE fig|380021.185.peg.1583 2,3-dihydroxybenzoate-AMP ligase (EC 2.7.7.58) of siderophore biosynthesis

ATGTCCACTTTCGATGACCTCAAGGATTGCCCCTCTTGGCCCGAAGACTTCGCCCAGCGC

TACCGCCAGGCCGGCTACTGGCGCGACGAAACCTTTGGCGACCTGCTGCGCAGCGCCGCC

CAGGCCTTTGCCGAGCGTGAAGCACTGACCGAGGGCGAGCAGCACCTGAGCTACCGGCAA

CTGGACCTGCGGGTCGACCAACTGGCCGCCGGCTTATACCGGCTGGGTCTGCGGGCCGGC

GACAACGTGGTGCTGCAACTGCCCAACAGTGCGGCCTTCGTCGAAGTCTGCTTTGCCCTT

TACCGCCTCGGGGTGCGGCCGATCTTCGCGCTGCCGGCCCACCGGCACCTGGAAATCGGC

CGCTTCTGCGAGTTCGCCCGGGCCCGGGCCTACTTCTGCGCCGACCGGGACGCCAGCTTC

GACTATCGGGCCATGGCCCGCGACCTCAAGGACCGCAACCCGCAGCTGGAGTGGGTGGTA

GTCGCCGGCGAGGCCGAGGAATTCACGGCCTTGCACAGCCTGTATGAGCCGGCGCCTGCG

CGCACCTTCCCGTCCCCCAGCGCCGACGCGGTGGCCTGTTTCCAGCTCTCCGGCGGCTCC

ACCGGGGTGCCCAAGCTGATCCCGCGCCGGCACCACGAATACCTCTACAACCTGCGGGCC

AGCGCCGAGCGCTGTGGCCTGTCCGAGGCCAGCGTGTACCTGGTGGCGCTGCCCATGGCG

CACAACTTTCCCATGTGCTGCCCAGGGTTCATCGGCACCTTTTCGGTGGGCGGGCGGGTG

GTGCTGAGCCCCTCGCCGAGCCCGGAAGTCTGCTTCGAACTGATCGAGCGCCAGGGGGTG

ACCCACACCGCCCTGGTGCCGCCCCTGGCCCTGGTCTGGCTGGAAGCGGCCCAGGCCCGC

GGGCGTGGCCTGGTGCCGTTGCAACTGCTGCAAGTGGGCGGCGCCAAGCTCAGCTACGAG

GCCGCAAGGCGCATCGAACCGGTGCTCGGCTGCCGCCTGCAGCAGGTGTTCGGCATGGCC

GAGGGGCTGATCTGCTACACCGACCCCGAGGACCCGCCACAGCGGGTGCTGCACACCCAG

GGCCGGCCGCTGTCGCCGGCGGACGAAATTCGTGTGGTGGACGAGCACGACCAGCCGGTG

CCGGTGGGGCAGGTGGGGCAGTTGCTGACCCGCGGCCCCTACACCATCCGCGGTTACTAC

CGTTACCCCGAGCACAACGCCCAGGCCTTCACGGCCGATGGTTTCTACCGCACCGGCGAC

CGGGTCATGCTCACTGCCGACGGCTACCTGATGGTGGAAGGGCGCGACAAGGACCTGATC

AACCGCGGGGGCGAAAAGATCGCTGCCGAAGAAGTGGAAAACCTGCTGCTCAGCCACCCG

TCGGTGGCCGACATCGCCCTGGTGGCGATGCCTGATGCCTTCCTGGGCGAACGCACCTGC

GCCTTCGTCATCCCCCGCGGCACCGCGCCCCGGGCCCCGGAGCTGCTGCGTCACCTAAGG

GCCCAGGGCCTGGCGGCGTTCAAGCTGCCGGACCGCTTCGAGTTCATCCCGGCCTTCCCC

CAGACCGGGGTCGGCAAGGTCAGCCGCAAGCACCTGCGCGAGGCGATCCAGGCCCTGTAC

TTCGGCGCCCAGGCCGAACCCCTGGAGGGAAGTGGCGCCCGTGGCTGA

>motA fig|380021.185.peg.5407 Flagellar motor rotation protein MotA

ATGGCTAAAATTATCGGCATCATCGTCGTATTCGCGAGCGTGCTCGGCGGATACGTGCTT

TCCCACGGCAAAATCGCGGCGCTGATCCAGCCTTTCGAAGTGCTGATCATCGGCGGCGCA

GCCTTTGGCGCATTCCTCCAGGCCAACCCGGGCTACATGACGATGCACGTCGTCAAGAAA

TCCCTGGGCATGTTCAGCTCGCGCTTCACCCACACTTTCTACCTGGAAGTGCTGGGCCTG

ATCTACGAGATCCTCAACAAGAGTCGTCGCGAAGGCATGATGGCCATCGAAGGCGATATC

GAGGACGCCGCGGCCAGCCCGATTTTCGCCAAGTACCCGGCGGTGCTCAAAGATGAACGC

ATGACTGCCTTCGTCTGTGACTATCTGCGCATCATGTCCTCCGGCAACATGGCTCCCCAC

GAGCTGGAAGGCCTGTTCGACATGGAGCTGTTCAGCCTCAAGGAAGACCTCGAGCATCCA

TCCCATGCGGTCAACGGGATTGCCGACGGCATGCCCGGTTTCGGTATCGTCGCGGCGGTA

TTGGGGATCGTGGTGACCATGGCCTCCCTGGGCGATGGCGACCAGAAGTCCATCGGCCTG

CACGTAGGTGCGGCGCTGGTGGGTACTTTCTTCGGTATTCTCGCGGCCTATGGTTTCTTC

GGCCCGCTGGCCCACTCCCTGGCCCACGACGCCAAGGAAGAACTGAACGTCTACGAAGCC

ATCAAGGCGTCGCTGGTGGCCTCGGCTTCCGGCATGCCGCCGTCCCTGGCCGTGGAGTTC

GGGCGCAAGGTTCTGTACCCGGCGCACCGTCCTAGCTTCGCCGAGCTGGAACAAGCGGTT

CGCGGTCGCTAA

>flgI fig|380021.185.peg.3066 Flagellar P-ring protein FlgI

ATGCTCAATTTCAAGCACCTGATGGCGGCGGCGTTGTTGCTGTCCACTTCCCTTGGCGTC

CAGGCCGAGCGGTTGAAGGACATCGCCAGCATTTCCGGCGTGCGTTCCAACCAATTGATC

GGCTATGGCCTGGTGGTCGGGCTTAACGGTACCGGCGACCAGACTACCCAGACGCCTTTC

ACCTTGCAGACCTTCAACAACATGCTGTCGCAGTTCGGCATCAAGGTGCCGGCCGGCTCC

GGCAACGTGCAGTTGAAGAACGTCGCGGCGGTGTCGGTGAGTGCCGATCTGCCGGCGTTC

GCCAAGCCGGGCCAGCAGGTGGATATCACCGTGTCCTCCATTGGTAACTCCAAGAGCCTG

CGCGGCGGCACCTTGCTGCTGACGCCGCTCAAGGGCATCGACGGCAACGTCTACGCCATC

GCCCAGGGCAACCTGGTGGTGGGTGGTTTCGATGCCGAAGGACGCGACGGTTCGAAGATC

ACCGTCAACGTACCGTCGGCCGGGCGGATTCCTGGTGGCGCCTCGGTCGAGCGTGCCGTG

CCCAGTGGTTTCAACCAGGGCAACAGCCTGACCCTGAACCTCAACCGTTCGGATTTCACC

ACCGCCAAGCGCATCGTCGACAAGATCAACGACATGCTCGGCCCAGGCGTGGCCCAAGCC

ATCGACGGTGGCTCGATCCGGGTGACCGCGCCCCTGGACCCAAGCCAGCGTGTGGACTAC

CTGTCGATCCTGGAGAACCTGGAGATCGATCCGGGGCAGGCGGTGGCCAAGGTCATCATC

AACTCCCGTACCGGCACCATCGTCATTGGCCAGAACGTCAAGGTCTCGCCGGCGGCGGTG

ACCCACGGCAGCCTGACCGTGACCATTACCGAAGACCCGATCGTCAGCCAGCCCGGCCCT

CTGTCCAATGGCCAGACCGCGGTGGTGCCCCGCTCGCGGGTCAATGCTCAGCAGGAAGCC

AAGCCGATGTTCAAGTTCGGCCCGGGCACCACTCTGGATGAGATCGTCCGGGCGGTGAAC

CAGGTGGGCGCGGCGCCAGGCGACTTGATGGCCATCCTCGAAGCCTTGAAACAGGCCGGC

GCCTTGCAGGCCGACCTGATCGTGATTTGA

>flgH fig|380021.185.peg.3065 Flagellar L-ring protein FlgH

ATGAATCGGTTTATTTGTGTTCTAGCGCTGAGTGGGAGTGCCGTGCTCGCGGGCTGTGTC

GCCCCGCCGCCCAAGCCCAATGACCCTTACTACGCGCCGGTGCTGCCGCGTACGCCGCTG

CCTTCCGCGTCCAACAACGGTTCGATCTACCAGGCCGGCTTCGAGCAGAACCTGTACAGC

GACCGCAAGGCGTTCCGGGTCGGTGACATCATCACCATCACCCTGAACGAGCGGACCAAC

GCCAGCAAGGGTGCCAACTCGGCGCTGACCAAGACCAGTTCCAACAGCATTGGCCTGACC

TCGCTGTTCGGTGCCGTGCCCAACACCAACAACCCGCTGGGCGACGGCGACCTGACCCTG

AATGCCGGCTACAGCGGCAATCGCGCCACCAAGGGCGACAGCAAGGCGGCCCAGAGCAAT

AGCCTGACCGGTTCGATCACCGTGACCGTGGCCGACGTACTGCCCAACGGCATCATCGCG

GTGCGTGGCGAGAAGTGGATGACCCTCAACACCGGCGATGAGCTGGTGCGGATTGCAGGC

CTGGTACGGGCGGACGACATCGCCACCGACAACACCGTGTCCTCGACCCGGGTCGCGGAT

GCACGCATTACCTATTCGGGCACCGGCTCGTTTGCCGATGCCAACCAGCCTGGCTGGTTC

GACCGTTTCTTCCTCAGCCCGCTGTTCCCTTTCTAG

>flgG fig|380021.185.peg.3064 Flagellar basal-body rod protein FlgG

ATGCTTCCGGCTCTATGGGTTGCCAAAACCGGTCTGTCCGCCCAGGACACCAACCTGACC

GTCATTTCCAACAACCTGGCGAACGTCTCGACCACGGGCTTCAAACGTGATCGCGCCGAG

TTCCAGGACCTGCTGTACCAGATCAAGCGTCAGCCGGGGGCCCAGTCCACCCAGGACAGC

GAGCTGCCAACCGGCCTGCAGGTCGGTACCGGTGTGCGCATTGTCGGCACCCAGAAGAAC

TTCACCGCCGGCAGCCTGCAGACCACCGAGCAACCTCTGGACCTGGCCATCAACGGACGC

GGTTTCTTCCAGATCCTGCAGCCCGATGGCACCACGGCCTACACCCGTGACGGTACTTTC

CACCTGAACTCCGACGGCCAGATCGTCACCGCCAGCGGTTTTGCCCTGGAGCCTGCGGTT

GTCGTGCCCAACGATGCGCAGACCTTCACCGTAGGCCAGGACGGCACCGTGTCCATCACC

ATTGCCGGCAACCCGGCGTCCCAGGTGATCGGCAACCTGCAAACCGCCGACTTCATCAAC

CCGGCGGGCCTGCAGGCCCAGGGCAACAACCTGTTCCTGGAAACCGCCGCCAGTGGCGCG

CCGCAGATCGGTACCCCGGGCCTCAACGGTTTCGGCACCACGCTGCAGAACACCCTGGAA

GCCTCCAACGTCAGCACCGTTGAGGAGATGGTCAACATGATCACCACTCAGCGCGCTTAC

GAGATGAACTCCAAGGTGATCTCCACCGCGGACCAGATGCTCTCGTTCATTACGCAGAAG

CTGTAA

>flgF fig|380021.185.peg.3063 Flagellar basal-body rod protein FlgF

GTGGACAAGTACCTTTATGTGGCAATGACCGGCGCCAGCCAGAATGCACTGGCGCAAAAG

GCTCATGCCAACAATCTGGCGAACATCTCTACCAATGGTTTTCAGCGCGACCTGGAGCAG

GCCCGTGCGATGCCGGTATTTGGTGACAGCTTTCCGGCGCGCGCCTTTGCCATGAGCGAG

CGCCCGGCAACGGATTTCAGTGCCGGCTCGATGGTGGAGACCGGTCGTGACCTGGACGTG

GCCGTCAGCGGCAACGGCTGGATCGCCGTGCAGAGCCCCGACGGCAGCGAAAGCTATGTG

CGTACCGGCAGTTTGAATGTCGACGCCCTGGGTGTGCTGCGCGCCGGCAACGGCATGCCG

GTGATGGGCAATGGCGGCCCGATCGCCGTGCCACCCGAGCAGAAGATCGAAGTTGGCCAG

GACGGCACCATCAGTATCCGTGCCATGGGCGAGGGCCCGCGAGTCATGGCCGAGGTCGAC

CGCATCAAGCTGGTCAACCCGGACATCAAGAACCTCACCAAAGGCCTGGACGGCTCGATC

AAGACCAAGGACGGCCAGCCGGCACCCGCCGATGCCAATGTCCAGGTGGTCTCGGGCTTC

CTGGAGTCGAGCAACGTCAACGCCGTTGAAGAGATGACCGCGGTGCTGGCCCTGTCCCGG

CAGTTCGAATTGCACGTGAAGATGATGAACAGCGCCAAAGAAGACGACCAGGCCATGGCT

CGGGTCTTGCAGATCAGCTAA

>flgD fig|380021.185.peg.3862 Flagellar basal-body rod modification protein FlgD

ATGAGCGTTACTGATACCACCAACGCCCCATCCCTCAAGGATATCCTGGCGAATTCGTCG

AAAACGACGAGCAATACCAGCGCCGACGGCCTGGCCGCGGCGACCAACAGCGCCACCGGC

AAACAGAGCCTGGGCAAGGACGCATTCCTCAAGCTGCTGGTAACCCAGCTGAACAACCAG

AACCCGCTGGATCCGCAGGACAACAGTGCGTTCGTTGCGCAGTTGGCGCAGTTCTCCACC

CTGGAAGGCATCACCACCCTCAACTCCTCGGTGAATGCGATCACGGGTAACTACAAGTCT

TCCCAGGCCTTGCAGGCTTCGTCCCTGGTGGGGCGCTCGGTCATCGTGCAGACCGGCACT

ACCCAGGTCGACACCAGCAAGAGCATGACCGGCTCGGTCACCGTGCCATCCAGCGTGAAC

TCGGTCACCGTGACCGTCACCGACAAGGACGGCAATACCGTCAAGACCATCGACATGGGC

AGCCAGAAGGCCGGCAACGCCAGCTTTGTCTGGGATGGCACCAAGACTGACGGCACCAAG

GCGGATCCGGGCAACTACACCTTCAAGGCCAGCACCACCATCGATGGCAAGGGCACTGAT

CTGATCACCTACCTGCCGGCCACGGTCAATAGCGTGACCATCAGCCAGACCGGCGGCGAG

TTGATGTTGAACCTCGCCGGCATGAGCAGCCCGATTGCCCTGTCCAAAGTACAAACCATT

GGTATCTAG

>flgC fig|380021.185.peg.3861 Flagellar basal-body rod protein FlgC

ATGTCCCTCGCCAGTGTTTTCAATATTGCCGGTAGTGGCATGAGCGCCCAGACCACCCGT

CTGAACACCGTCGCCAGTAACATCGCCAACGCCGAGAGCGTCTCGTCGAGCATCGACCAG

ACCTACCGCGCTCGTCACCCGGTGTTTGCCACCATGTTCCAGGGTGCGCAAAGCGGCGGC

AGCGACTCGCTGTTCCAGAACCAGGACGCTGCCGGCCAGGGCGTGCAGGTACTGGGTGTG

GTCGAAGACCAGAGCAACCTCGAAGCTCGTTACGAGCCGAACCATCCCGCCGCTGATGCC

AAGGGTTATGTCTACTACCCCAACGTCAACGTGGTGGAAGAAATGGCCGACATGATTTCT

GCCAGCCGTTCGTTCCAGACCAACGCTGAAATGATGAACACCGCCAAAACCATGATGCAG

AAGGTCCTGACCCTGGGTCAGTGA

>flgB fig|380021.185.peg.3860 Flagellar basal-body rod protein FlgB

ATGAGCATCAGCTTCGATAAAGCGCTCGGTATCCACGAACAGGCCCTTGGCTTTCGCGCC

AAGCGTGCCGAAGTTCTGGCCAACAACATCGCCAACGCCGATACCCCGAACTACAAGGCT

CGGGACCTGGACTTCTCTGCCGTGCTTGCCGAGCAGAATCAGAAGGCCCAGAACGGTACC

TTCGCGTTGAACATGACCAACAACCGGCATATCGAAGCCCAGGGCTTGAGCAGTGGTGAT

GAATCGCTGCTGTATCGCACCCCGATGCAACCTTCGATCGACCAGAACACCGTGGACGCC

CAGCTGGAGCAATCGAGCTACGCGCAGAACTCGGTGGATTTCCAGGCCAGCTTCACGCTG

CTCAACAGCAAATTCAAAGGGCTGGTAGCAGCCCTGCGCGGAGAGTAA

>motB fig|380021.185.peg.5408 Flagellar motor rotation protein MotB

ATGGAAAATAATCAGCCGATTATCATCAAGCGCGTCAAGCGCTTCGCTGGCGGACATCAC

GGGGGCGCCTGGAAAATCGCCTTCGCCGACTTCGCCACGGCGATGATGGCGTTCTTCCTG

GTGTTGTGGCTGCTGTCCACGGCCACCCCGGAACAGAAGATCGCCATCGCCGGTTACTTC

AAGGACCCGATCGGCTTCTCGGAAAGCGGTACGCCCTACATCATCGACCTGGGCGGCTCC

CCTGAGCTGGCGCCGGACAACACCCTCAACCCCGAGATCAAGTCCCAGCCACAACCGGAC

AAGGTCACGGTGGATGCCGACCAGGTGGAGGGCATGGCCGAGCAGGTCGAGCGCGAGCGT

CTCGAGTTGCTGTTGCAAGAGTTGCAGAACAAGGTTGAAGAAAACCCGCAACTGCAGAAG

TTCAAGGACCAGATCCTCTTCGAGATCACCCCGGACGGCCTGCGGATCCAGATCATGGAC

GCCGAGAACCGGCCGATGTTCGACTCTGGCAGTGCACGTCTGAAGCCGTATTTCGAAGAC

ATCCTGCTGGCCATGGCTGACACCATCAAGACCGTGCCGAACAAGATCAGCATCAGCGGT

CACACCGACGCCAAGCCCTATTCGGGCACTGGCGACTTCGGCAACTGGGAACTGTCGGCC

AACCGGGCCAACGCTGCGCGTCGGGCGCTGGTGGCGGGTGGTTATCCTGATCCGCAAGTT

GCGCGTGTGGTGGGCTTTGCTTCGTCGGCTCTATTCGATCGCAAGGACCCGTTCAACCCG

GTCAACCGGCGCATCGATATCGTCGTGCTGACCAAGAAGGCCCAGCGTGCCATCGAGGGT

GAGCAGACCGATCCCAATGCTCCGGTACCGACCCAGGGTAGCGGTGCTCCCGGTGAAGTA

CCGGGGGCGGCAGCCGACCCGAATGCCTTGCCGCCCGGTAGCGAACCGCTGCCGGCGCAC

GAGGTGCGTCAACGTATGAATATCTTCGAGGACGGTGTGCTGAAGATGGACGAGCAGGGT

GCGGCGGGCCAGTCTTCCGCTCCGGCTCCGGCTCCCACTGCTCCGGTGGCGCCGCCCGCT

ACGCCGGGCACGGCGAAGTGA

>fepA fig|380021.185.peg.716 TonB-dependent receptor; Outer membrane receptor for ferric enterobactin and colicins B, D

ATGTCCATGCGCTCGATGCGTCCTTTCGCCCCCTTCCTGCTGTCCACCTGCTGCCTGCTC

AGCCAGGCCGTGCAGGCTGCCAATGAAGGCTCGACCCAGCCGGTCCTGGAGCTGGAACCC

CAGAGCGTCATCGCCACCGCCAAGGAAGAAACCAAGCAGGCACCGGGGGTCTCTGTGATC

ACCGCCGAGGACATCCAGAAGCGCCCGCCGGCCAACGACCTGTCGCAGATCATCCGCACC

ATGCCCGGGGTCAATCTCACCGGTAACTCCACCAGCGGCCAGCGCGGCAATAACCGGCAG

ATCGACATCCGCGGCATGGGCCCGGAAAACACCCTGATCCTGGTGGACGGCAAGCCGGTG

GGCAGCCGCAACTCGGTGCGCTACGGCTGGCGCGGCGAGCGTGACAGCCGCGGCGACACC

AACTGGGTACCGGCCGACCAGGTCGAACGCATCGAAGTGATCCGCGGCCCGGCGGCGGCC

CGCTACGGCAACGGCGCCGCTGGCGGGGTGATCAACATCATCACCAAGCAGGCCGGCATC

CAGACCCACGGCAATGCGACGATCTACAGCTCGTTCCCGACCCACAAGGACGAAGGGGCC

ACCCAACGCATGAGCTTCGGCCTCAACGGCCCGCTGACCGACAGCTTGAGCTACCGGGTC

TACGGCAACGTGGCCAAGACCGATTCCGACGACGCCGACATCAACGCCGGCCATGAATCC

CTGCGCACCGGCAACCAGGTCGGCACCCTGCCCGCCGGCCGCGAAGGGGTACGCAACAAG

GACCTCAACGGCCTCTTGAGCTGGCACCTGACCCCGGACCAGAGCCTGGACTTCGAAGCC

GGCTTCAGCCGCCAGGGCAACATCTACACCGGCGATACCCAGAACACCAACAGCAACAGC

ACGGTGAAAAACCTGCTGGGCCACGAGACCAACCGCAGCTACCGCGAGACCTATTCGGTG

ACCCATCGTGGCGAATGGGACTTCGGCAGTTCCATGGCCTACCTGCAATACGAAAAGACC

CGCAACACGCGGATCAACGAAGGCCTGGCCGGCGGCACCGAAGGCATCTTCAGCAACAGC

GAGTTCTACACCGCGGTGCTCCGCGACCTGACCGCTCATGGCGAGGTCAACCTGCCCCTG

CGCGCCGGCTTCGAGCAGACCCTGACCCTGGGCAGCGAATGGTCCCAGCAAAAGCTCGAC

GACCCCAGCGCCAACACCCAGAGCACCAGCGAAGGCGGCGCCGTGGGCGGCCTGAGCTCA

AGCAACCGCAGCACCCAGTCCAGCGCGCAGATCTTCTCGCTGTTCGCCGAAGACAACATC

GAACTGCAGCCGGGCACCATGCTCACCCCGGGCCTGCGCCTGGACCACCACAGCATCGTC

GGCGACAACTGGAGCCCGTCCCTCAATCTGTCCCACGCCCTGAGCGACACCCTGACCCTC

AAGGCCGGCATCGCCCGGGCCTACAAGGCGCCGAACCTGTATCAGCTCAACTCCGACTAC

CTGCTCTACAGCCGCGGCCAGGGCTGCTATGGCCAGAGCACCAGCTGCTACCTGCAGGGC

AACGACAAGCTCAAGGCGGAAACCAGCGTCAACAAGGAACTGGGCCTGGAATACAAGGCC

GACGGCTGGGTGGCCGGCCTGACCTACTTCCGCAACGACTACAAGAACAAGGTGGAATCC

GGCCTGGCCCCGGTGAGCCATGCCAGCGGCGGCAGCGGCGCTTACCGCAACTCGGCGATC

TACCAGTGGGAAAACGTGCCCAAGGCCCTGGTGGAAGGCCTGGAAGGCACCCTGACCATT

CCGTTGGCCAGCCAGCTGACCTGGAACAACAACTTCACCTACATGCTGCAATCGAAGAAC

AAGGAAACCGGCGACTACCTCTCGGTGACCCCGCGCTACACCCTCAACTCGATGCTCGAC

TGGCAGGCCACCCAGGACCTGTCGCTGCAGGCGACCGTGGCCTGGTACGGCCAGCAGACG

CCGAAGAAATACGACTACCACGGCGACCGCGTCACCGGCAGCGCCACCCAGCAACTGGCG

CCGTACGCCATTGCCGGGGTCAGCGGCACCTACGCCCTGACCCGCAACCTGAGCCTGACC

GCCGGGGTCGACAACCTGTTCGACAAACGCCTGTTCCGTGAAGGCAATGCCCAGGGCGTG

AACAACATCGCCGGGGCCGGCGCCGCCACCTACAACGAACCCGGGCGCACCCTGTACACC

AGCCTGACCGCTTCGTTCTGA

>algB fig|380021.185.peg.4943 Alginate biosynthesis two-component system response regulator AlgB

ATGGAGTCAGCGCCGGAGAATCAAGGCCGCATCCTTCTGGTGGATGACGAATCCGCAATC

CTTCGCACCTTCCGCTACTGCCTGGAAGACGAGGGCTACACCGTGGCCACCGCCAACAGC

GCGGCGCAGGCCGATGCTCTGCTGCAACGCCAGGTATTCGACCTGTGCTTCCTCGACCTG

CGCCTGGGCGAAGACAACGGCCTCGACGTACTGGCACAAATGCGTACCCAGGCACCCTGG

ATGCGGGTAGTGATCGTCACCGCTCACTCTGCCGTGGACACCGCAGTAGATGCGATCCAG

GCCGGCGCCGCAGACTACCTGGTCAAGCCCTGCAGCCCCGACCAGTTGCGCCTGGCCACC

GCCAAGCAGCTGGAAGTACGCCAGCTCTCGGCACGCCTGGAAGCCCTCGAAGGTGAGGTG

CGCAAGCCCAAGGACGGCCTGGACTCCCACAGCCCGGCAATGAAAGTGGTGCTGGAAACC

GCGCGTCAGGTGGCCAGCACCGACGCCAACATCCTTATCCTCGGCGAGTCCGGCACCGGC

AAGGGCGAGCTGGCGCGGGCCATTCACGGCTGGAGCAAACGCGCCAAGAAATCCTGCGTG

ACCATCAACTGCCCCTCGTTGACTGCCGAACTGATGGAGAGCGAACTCTTCGGCCACAGC

CGCGGCGCCTTTACCGGGGCCAGCGAGAGCACCCTGGGCCGGGTCAACCAGGCCGATGGC

GGAACCCTATTCCTCGACGAGATCGGCGACTTCCCGTTGACCCTGCAACCCAAGCTGCTG

CGCTTCATCCAGGACAAGGAATACGAGCGCGTCGGCGACCCGGTAACCCGCCGCGCCGAT

GTGCGCATCCTCGCCGCCACCAACCTCAACCTTGAAGACATGGTGCGCGACGGGCGTTTC

CGCGAAGACCTGCTGTACCGGCTGAACGTCATCACCCTGCACCTGCCGCCCCTGCGCGAG

CGCAGCGAAGATATCCTGACCCTGGCCGACCGCTTCCTCGCCCGCTTCGTCAAAGAGTAT

TCGCGCCCGGCCCGGGTCTTCAGTGATGAAGCCCGGGAAGCGCTGCTCAACTACCGCTGG

CCGGGCAACATCCGCGAACTGCGCAACGTGGTGGAACGGGCGAGCATCATCTGCCCGCAG

GAACGGGTGGAAATCAGCCACTTGGGCATGGCCGAGCAACCCACCAACAACGCCCCGCGG

ATCGGCGCCGCCCTGAGCCTGGACGAGCTGGAAAAGGCCCACATCGGTGCGGTCCTGGCC

ACCAGCGACACCCTGGATCAAGCCGCCAAGACCCTGGGTATCGACGCGTCGACGCTGTAC

CGCAAACGCAAACAGTACAACCTGTGA

>algD fig|380021.185.peg.2462 GDP-mannose 6-dehydrogenase (EC 1.1.1.132)

ATGCGCATCAGCATATTTGGTTTGGGTTACGTCGGTGCAGTATGTGCCGGTTGCCTGTCC

GCACGGGGCCATGAAGTGGTTGGCGTAGATATCTCCAAAGATAAGATCGACCTGATCAAC

GCCGGCAAGTCGCCGATCGTCGAACCGGGCCTGGGCGAACTTCTGGCGCAAGGAATCCAG

ACCGCTCGTCTGCGTGGCACCACCAACTTCGCCGAGGCCATTCGCGATACCGACCTGTCG

ATGATCTGCGTCGGTACCCCGAGCAAGAAGAACGGCGACCTGGAACTGGACTACATCGAA

TCGGTGTGCCGCGAGATCGGTTTCGTCCTGCGCGACAAGGCCACCCGCCACACCATCGTG

GTGCGCAGCACCGTGCTGCCGGGCACCGTGAAGAACGTGGTGATCCCGATCCTCGAAGAC

TGCTCGGGCAAGAAGGCCGGGGTGGATTTCGGCGTCGCGGTGAACCCCGAGTTCCTGCGT

GAAAGCACCGCGATCAAGGACTACGACTTCCCGCCCATGACCGTGATCGGCCAGTTCGAC

CAGGCCTCCGGTGATGTGCTGCAAGCGCTGTACGAAGAGCTCGATGCACCGATCATCCGC

AAGGACATCGAAGTCGCCGAGATGATCAAGTACACCTGCAACGTCTGGCACGCCACCAAG

GTCACCTTCGCCAACGAGATCGGCAACATCGCCAAGGCGGTGGGCGTGGACGGCCGTGAA

GTGATGGACGTGGTCTGCCAGGACAAGACCCTCAACCTGTCCCAGTACTACATGCGCCCG

GGCTTCGCCTTCGGCGGCTCGTGCCTGCCCAAGGACGTGCGCGCCCTGACCTACCGCGCC

GGCTCCCTGGACATTGAGGCGCCCCTGCTCAACTCGCTGATGCGCAGCAATGAATCCCAG

GTGCAGAACGCCTTCGACATCGTTTCCAGCCATGACAAGCGCAAGGTCGCCCTGCTGGGC

CTGAGCTTCAAGGCCGGCACCGACGACCTGCGCGAGAGCCCGCTGGTGGAACTGGCGGAA

ATGCTCATCGGCAAGGGCTTCGACCTGAGCATCTACGACAGCAACGTCGAGTACGCCCGG

GTTCACGGCGCCAACAAGGACTACATCGAGTCGAAGATCCCCCACGTTTCGTCCCTGCTC

AACTCGGACTTCGACGCGGTAATCGGCAACTCCGACGTGATCATTCTCGGCAACCGCGAC

GAGAAGTTCCGCGCCCTGGCCGAAGACGTGCCCCACGGCAAGCAGGTCATCGACCTGGTG

GGCTTCATGTCCAAGGCTACCTGCAGCAAAGGCCGTACCGAAGGGATCTGCTGGTAA

>algK fig|380021.185.peg.2459 Alginate export system AlgK/AlgE, periplasmic component AlgK

ATGCCTGTGACTAGCCTTTTCATTGGCGAGTGCTGCGCACTCGATCGCGAGCAAGCTCGC

TCCTACCAGAACACCGCCGCCTCCGGTAGGAGCGAGCTTGCTCGCGAAAGCGCTCAAGGC

CGCCGCCATCTGGCCCTGTGCGCCCTGGCGCTGGCCGTGAGCCTGGCCGGTTGCGCCGGC

CTGCCGGACCAGCGCCTGGCCAACGAGGCCCTGAAGCGCGGCGACACCGCCCTGGCCCAG

CAGAACTACCGCCAACTGGCAGACCTGGGCTACAGCGAAGCGCAAGTGGGCCTGGCCGAC

ATTCAGGTGGAAAGCCGCGACCCGGCGCAGATCAAGCAGGCCGAGGCCACCTACCGCGCC

GCGGCCGACACCTCGCCCCGTGCCCAGGCGCGCCTGGGGCGCCTGCTGGTGGCCAAGCCC

GGCTCCAGCGAGGCCGAGCAGCACGAAGCCGAAGGCCTGCTGAAGAAAGCCTTTGCCAAT

GGCGAAGGCAACACCCTGATCCCCCTGGCGATGCTGTACCTGCAATACCCCCACAGCTTC

CCCAACGTCGACGCCCAGCAGCAGATCAGCCAATGGCGCGCCGCCGGCTATCCGGAAGCC

GGTCTGGCCCAGGTCCTGCTGTACCGCACCCAGGGCACCTACGACCAGCACCTGGACCAG

GTGGAAAGCATCTGCAAGGCCGCCCTCGACAGCACCGACATCTGCTACGTCGAGCTGGCC

ACCGTCTACCAGAAACGCGGCCAGCCAGAACAGCAGGCCGAGCTGATCAAGCGACTGCAG

GCCGGCTACAGCCGTGGCAGCGTCAGTGCCCAGCGGGTCGACAGCGTGGCCCGGGTACTG

GGCGATGCCAGTCTTGGCAAGCCGGACGAAAAGACCGCCCAGGCCCTGCTGGAACAGGTC

GCCCCCGGCTACCCGGCCTCCTGGGTCAGCCTGGCGCAACTGCTCTACGACTTCCCCGAG

CTGGGTGACGTCGACAAGATGATGGAATACCTGGAGAACGGCCGCGCCGCCGACCAGCCC

CGGGCCGAACTGCTGCTGGGCAAGCTCTACTACGAAGGCAAGTGGGTGCCGGCGGACGCC

AAGGTGGCCGAGGCGCACTTCCAGAAAGCCGTGGGCCGCGAAGTCGCCGCCGACTACTAC

CTGGGGCAGATCTACCGCCGTGGCTATCTGGGCCAGGTGTACCCGCAAAAAGCCCTGGAC

CACCTGCTGACGGCTGCCCGCAACGGCCAGAACAGCGCCGACTTCGCCATTGCCCAGCTG

TTTTCCCAGGGCCGCGGCACCGCGCCCAACCCGGTCAACGCCTACGTCTTCAGCCAGTTG

GCCAAGGCCCAGGACACCCCACAGGCCAACGAACTGGCCCAGCAGCTGGATCAACAACTG

CCCCCCGGCCAGCGCGCCGAAGCCCAGCGCCTGCTGCAACAGGAGCAGGCGGTACGTGGC

GCCCTGACCCAGAACACGCTGCAACTGCACGCCCTGCAGGAAGAAGACGGCGAGGAATCC

CTATGA

>algE fig|380021.185.peg.2458 Alginate export system Algk/AlgE, outer membrane porin AlgE

ATGAAGCTCACCCCATTCAACACCGCCCCTGTAGCCGCTGCCGCAGGCAAGCGCTTCAGC

CTGTCGAAACTGGGCATGGCCCTGGGTTTTGCCATGATCTGGTCCAACCCGACCCTGGCG

GCCATCACCGACACCAAGAACTTCGGCCTGGAAGTGAAGATCACCGGCCAGTCCGAGGAT

GACCGCGACCTGGGCACCCAGGGCGGTGGCGACGTCAACGGCATCGGCCTCGACCTGCGG

CCCTGGGTCTATGGCGAAAGCGGCAACTGGAGCGCCTACGCCATGGGCCAGGCAGTGACC

GCAAGCGACGTGATCGAAACCGACACCCTGCAACAGTCCGGCGACGACGCCAGCCAGCAG

TCCAGCAACGATGACCGCAAGACCAAGAAGAACTACCTGGCCATGCGCGAGTTCTGGGTC

GGCTACAGCGGCTTCACCGCCTACCCCGGCGAGCAGTTGAAGTTCGGCCGCCAGCGCCTG

CGCAACGATGACGGGCAATGGCGCGACACCAACATCGAGGCGCTGAACTGGACCTTCGAC

ACCACCCTGCTGCGGGCCAACCTGGGGGCCGCCGAGCGCTTCAGTGAATACCGCACCGAC

CTCAAGGAGCTGGCGCCCAAGGACAAGGACCGCCTGCACCTGTACGCCGACGCCGCCTAC

CAGTGGACGCCCGGCCAGTGGGTGGGCATCCGCGCCCACCATACCCACGACGACGGCAAG

CTCGACTATGCCCAGCCCGGCGTGGCCAGCGACCCGCTGGACAAGAAGGAAAACGGCGAC

CTGACCTGGCTCGGCCTGGAAGCCAACAGCGACGCCTTTAACTGGCGCAACACCAATACC

GTCAACTACTGGGCCAGCCTCACCGGCATGCGCGGCGACCGCGATACGGTCAACCCGCTG

AACGCCGATGGCAGCCGCCCGACCCAAGCCAAGCGCGGGGACAACCTCAACGGCTGGGCC

ACCGACCTGGGCGTGCGCCTGCGCCTTGACCCGAACTGGCAAGTGGGCGCGGCCTATGCC

CGGGCCAGCGCCGAGTACGAACAGAACGGCCTGCAAAGCAACCGCTCCAACTACACCGGT

ACTCGCTCCCGGGTGCACCGCTTCGGCGAAGCCTTCCGGGGCGAAATGAACAACACCCAG

AGCGCCACCCTGTTCGGTTCCTGGCAACTGCGCGAGGACTACGACGCCAGCCTGGTCTAC

CACAAGTTCTGGCGTGTGGATGGCAACAAACCGGTGGGCAGCAACGGCATCAACGCAGTG

GACAACAACTACGACGACACCACCGGCGCCCTGCTCAGCAGCACCTCGCTGCCGCTCATG

GATGGCAAGAAGGACCTCGGCCAGGAGATGGACCTGGTGGTCACCAAGTACTTCAAGCAA

GGCCTGTTGCCCGCGGCCCTGAGCCAGTCGATCGATGAACCTTCGGCCCTGGTGCGTTTG

CGCGGTGGCGTGTTCAAGCCCGGCGACGCCTACGGCAAAGAGGTCGACTCCTACATGCAC

CGCGCCTTTATCGACGTGATCTGGCGCTTCTGA

>algG fig|380021.185.peg.2457 Poly (beta-D-mannuronate) C5 epimerase AlgG (EC 5.1.3.-)

ATGCCTAATCAAACTCTCCAGAGCCCGTTGTCCAGAGGCTCCCTGAGCCTCCTGGCCGGC

GCCCTGCTGCTGGCCAGCAGCAGCGCCTTCGCCACTGCCGAGACGGCCAAGGCGCCGATC

ATCGCCAAGGAACTGCAACAGGCCAAGACCTACACCGTCAGCAGTGCTCCCATCGAGCCC

CTGGCCCTGGCCAAGCCGACCCTGCCCGACCTCTCCGGCTACACCGCCGAGGCGGCCGCG

GCGAAGATCGTGCGCAGCAAGCCGGGCAAGATCAGCGTGCGCCGGATGATGCAGGAAGAC

GCCCTGAAGGACTTTATCGGCGGTGACAACAAGATGGCCGAATGGGTGGTGCGCCAGCAC

GGCATACCCCAGGCGATCTTCATCGACGACGGCTACGTCAACCTCAAGGAACTGGTGGGC

AAGCTGCCCAAGCAGTACATCAGCGAAACCGCGCCCGGGGTCTACCTGGCCCGCCTGCCC

ATCGTCGTCGGCCGCCACGGCATCCTGGAAATCGACAAACAGACCCAGGAGCTGCGCCTG

TCCCAGGAAAGCGGCTCGTTCCTGGTCAACGATGGCCAGCTGTTCGTGCGTGACACCAAG

GTCACGGGCTGGCGCGAGAAGGACAACGGCCCGGCGACCTTCCGCTCGCCCAAGGAATTC

CGCCCGTTCCTGCTGGCCTGGGGCGGCACCGAGACTTACATCGTCAACAGCACGATGGCC

AGCTTCGGCTACGCCAACTCCAAGTCCTACGGGGTGAGTATTTCCCAGTACACGCCGAAC

ATGGCCAAGGTGCTCAAGCGCCCCGAGCCCACCGGCTGGATCGTCGGCTCGACGTTCTCG

GACATGTGGTACGGCTTCTACTGCTACGAGACCCGGGACTTCGTGCTCAAGGGCAACACC

TACAAGGACAACATCGTCTACGGCATCGACCCCCACGACCGCTCCCATGGCCTGATCATT

GCCGACAACACCGTCCACGGCACCAAGAAGAAGCACGGCATCATCATTTCCCGGGAAGTG

AACGACAGCTTCATCTTCAACAACCGCAGCTACGACAACCACCTCTCCGGGCTGGTGATC

GACCGTAACAGCGTCAACAACCTGATCGCCTACAACGAGATCTACCGCAACCACACCGAC

GGCATCACCCTCTACGAGAGTGCCGACAACCTGATCTACGGCAACAAGGTGATCAGCAAC

CGCCGCCACGGCATCCGCATTCGCAACAGCGTGAACATCCGCCTGTACGAAAACATCGCC

ATGGCCAACGGCCTGACCGGGGTCTACGGCCACATCAAGGACCTTTCCGACACCGACCGC

GACATCAAGCTCGACCCGTTCGACGCCCAGGTCTCGCTGATCGTGGTCGGCGGCGAGCTG

GCCGCCAACGGCAGCGGGCCGATGTCCATCGACTCCCCCTTGAGCGTGGAGCTGTACCGG

GTCTCCATGCTGGCGCCGACCAAAAGCAGCGGCATCAGCTTCTCCGGGGTCCTGGGCGAC

CGCCAGGATGAAATTCTCGACCTGCTGGTGCGCCAGCAGAAAGCCGTGTTGATCGACCCC

GTCGAACGCCAGACCGAATTGCGGGATTGA

>algX fig|380021.185.peg.2456 Alginate O-acetyltransferase AlgX, periplasmic

ATGCACCCACACTGGATCAAACTCCTGGGCCTGTCGGCCCTGACCGCGGGGATTCTCGCC

GCCAGCGCCGGCGCCCGTGCCGACGAAACCAGCGCCACAAGCGCCGCGCCGAACTTCAAG

GCCGAGCCGTGCTGCAACCTGTGCCCGGCCGCCCACGACGCAAAGAACTACGTGACCCGC

TACCAGCAGAACTTCACCACCCTGGTGCAAGCCCAGGGCGACTGGCTGTTCCGTACCCAG

GAAGATTTGCGCACCGAGTTCAACACCACTCCCGAAGGCTACCGCCGCCTCAAGCAGTTG

CACGAAGCCTTCAAGAGCAAGGGCGTGGAACTGGTGGTGGTCTATCAGCCGACCCGCGGC

CTGGTGAACCGCAACAAGCTCAACCCGGCGGAGAAAGCCCGTTTCGACTATGACAAGGCG

CTGAGCAACTACAAGGCCATGCTCGGGCGCTTCGCGCAGATGGGCTACGTGGTGCCGGAC

CTCTCGCCGCTGACCAACGAACAACTGCCCGATACCCTGCCGGCCCACGACTTCTACTTC

CGCGGCGACCAGCACTGGACCCCTTATGGCGCCCAGCGCACGGCGAAGATCGTCGCCGAC

AAGGTCAAGCAGTTGCCGGCCTTTGCCGACATTCCACGGCGTGAATTCGAAACCCACAAG

TCCGGGCGCATGGGCAAGACCGGCACCCTGCACAACATGGCCGGCCAGTTGTGCGGCACC

AGCTACGCCATCCAGTACATGGACCAGTTCACCACCGAGCCCAAGGGCGAGGCTGGCGAC

GGCGACCTGTTCGGCGACTCCGGCAACCCGCAGATCACCCTCGTGGGCACCAGCCACAGT

GGCAAGAACTACAACTTCGCCGGCTTCCTGGAAGAGGCCATCGGTGCCGACATCCTCAAC

GTCGCCTTCCCCGGCGGCGGCCTGGAAGGCTCGATGCTGCAGTACCTGGGCAGCGACGAG

TTCCAGAAGAGCCCGCCGAAGATCCTCATCTGGGAATTCTCGCCGCTGTACCGCCTGGAC

CAGGAAACCATCTACCGGCAGATGATGGCCCTGCTGGACAACGGCTGCGAGGGCAAGGAC

GCGCAGATGACCGGCAGCACCACCCTCAAGCCCGGCAAGAACGAATTGCTGGTCAACAGC

AAGAACATGGACCTGCGCAACAGCGGTCATCAGGTCGACATCCGCTTCGCCGACACCTCG

GTGAAAACCCTGCAAGCCACCCTCTGGTACATGAACGGGCGCCACGAGGACATCAAGATC

GAAAAACCGGAAACATCCGACACCGACGGGCGCTTCGCCTTCGAACTGCGCACCGACGAA

GACTGGGCCTCGCAGAACCTGCTGGCGGTCGAGATCCAGGGCCCGGAAGCGGGCACTGCG

GGGCAGAAGGTCGAAGCGAAAATTTGCAAACGCAACGTATTCCCGAGCGCCGAGCAACGC

ACCGCGCAGATCGGGCAATGA

>algL fig|380021.185.peg.2455 Alginate lyase AlgL (EC 4.2.2.3)

ATGCAAAGCACAGACTTGAAACGCTTGTTGATACCCAGCCTGCTGGGGCTGGCCATCGTC

ACCGGCAGCGCCCAGGCCGCCGCGCCGCTGCGCCCGCCCCAGGGCTATTACGCCCCGGTG

GACAAGTTCAAGAGCGGCGACAACAGCGAGGGCTGCGACGCCATGCCGGCGCCCTACACC

GGCGCCCTGCAGTTTCGCAGCAAGTACGAAGGCTCGGACAAGGCCCGGGCGACCCTCAAC

GTGCAGTCCGAACAGGCCTTTCGTGACACCACCGCGGACATCACCAAGATCGAGCGCGGT

ACCAGCAAGCGGGTCATGCAGTTCATGCGCGACGGCCGCCCTGAACAGCTGGACTGCACC

CTCGCCTGGCTCAGCGCCTGGGCCCAGGCCGATGCGCTGATGTCCAAGGACTTCAACCAC

ACCGGCAAGTCCATGCGCAAATGGGCCCTGGGCAGCATGGCCTCGGCCTACCTGCGACTG

AAGTTCTCCGACTCCCACCCCCTGGCCACCCACCAGGAACAGGCACAGAAGATCGAGGCC

TGGTTCAGCAAGATGGCCGACCAGGTGGTCAGCGACTGGGACAACCTGCCCCTGGACAAG

ACCAACAACCACTCCTACTGGGCCGCCTGGTCGGTGATGGCCACCGCCGTGGCCACCAAC

CGCCGCGACCTGTTCGACTGGGCGGTCAAGGAATACAAGGTCGGCGCCAACCAGGTCGAT

GCCGACGGCTTCTTGCCCAACGAACTCAAGCGCCAGCAGCGGGCCCTGGCCTACCACAAC

TACGCCCTGCCGCCGCTGGCAATGATCGCCAGCTTCGCCCAGGTCAACGGCGTGGACCTG

CGCCAGGAGAACAACAGCGCCCTCAAGCGCCTGGGCGAGCGGGTGCTGGCCGGGGTCAAG

GACCCGGACACCTTCGAAAAGAAGAACGGCAAGCAGCAGGACATGACCGACCTCAAGGTC

GACTCGAAATTCGCCTGGCTGGAACCCTACTGCAGCCTCTACACCTGCGCCCCGGAGACA

TTGGAGCGCAAGCACAAGATGCAGCCGTTCAAGACTTTCCGCCTGGGTGGCGACCTGACC

AAGGTCTACGACCCGGCCCATGAAAAGGGCTCCTGA

>algA fig|380021.185.peg.2451 Mannose-1-phosphate guanylyltransferase (EC 2.7.7.13) / Mannose-6-phosphate isomerase (EC 5.3.1.8)

ATGATTCCGGTGATCTTGTCAGGTGGTAGCGGCTCACGTCTTTGGCCGCTTTCGCGCAAG

CAATTCCCCAAGCAGTTCCTCGCCCTGACCGGCGAACACACTTTGTTCCAGCAGACCCTG

GAGCGCCTGGTGTTCGAAGGCATGGATACCCCCATCGTGGTCTGCAACAAGGACCACCGC

TTTATCGTCAACGAGCAACTGGCGGCCCGTAAGCTCGAATCCCAGCGCATCCTCATGGAG

CCCTTCGGCCGCAACACCGCCCCGGCCGTGGCCCTGACCGCGATGATGCTGGTCAACGAA

GGCCGCGACGAGCTGATGCTGGTACTGCCCGCCGACCACGTCCTGGACGACCAGAAAGCC

CTGCAACGGGCCCTGGCCCTGGCCACCGTGGCCGCCGAGCGTGGCGAAATGGTGCTGTTC

GGGGTCCCGGCAACCAAGCCGGAAACCGGCTACGGCTACATCAAGTCCACCAACGATGCG

CTGCTCCCGGAGGGGGTCAGCCGGGTCTCGCACTTCGTCGAAAAGCCCGACGAAAAACGC

GCCACCGAGTTTGTCCAGGCCGGCGGCTATTTCTGGAACAGCGGCATGTTCCTGTTCCGC

GCCAGCCGCTTCCTCGAAGAGCTGAAGAAACACGACCCGGACATCTACGACACCTGCCTC

TTGACCCTGGAGCGCAGCCAGCAGGACCCGGACACCGTGACCATCGACGAAGCCACCTTC

GCCTGCTGCCCGGACAACTCCATCGACTACGCGGTGATGGAGAAGACCCAGCGCGCCTGC

GTGGTGCCCCTGACCGCTGGCTGGAGCGATGTCGGCTGCTGGTCGTCGCTGTGGGACGTG

CATGAAAAAGACGCCAACGGCAACGTCAGCAAGGGCGATGTGGTGATCCAGGACAGCCGC

AACTGCATGATCCACGGCAACGGCAAGCTGGTATCGGTGATCGGCCTTGAAAACATCGTC

GTGGTGGAAACCAAGGACGCCATGATGATCGCCCACAAGGACAAGGTCCAAGGGGTGAAA

CAGATGGTCAACACCCTCAACGAACAGGGCCGCAGCGAAACCCAGAACCACTGCGAGGTC

TACCGCCCGTGGGGCTCCTACGACTCGGTGGACATGGGCGGGCGTTTCCAGGTCAAGCAC

ATCTCGGTCAAGCCGGGGGCCTGCCTGTCCCTGCAGATGCACCACCACCGCGCCGAACAC

TGGATCGTGGTCAGCGGCACCGCCGAAGTCACCTGTGACGAGAACGTGTTCCTGCTCACC

GAGAACCAGTCCACCTACATCCCGATTGCCTCGGTGCACCGCCTGCGCAACCCGGGCAAG

ATCCCGCTGGAGATCATCGAAGTGCAATCGGGCAGTTATTTGGGCGAGGACGATATTGAG

CGGTTTGAGGATATCTACGGCCGCTCGACGCCGGTGGAACGTGGCGTCTCGGTGAAGACC

ATCGCCCAGTAA

>aprA fig|380021.185.peg.531 Secreted alkaline metalloproteinase (EC 3.4.24.-), PrtA/B/C/G homolog

ATGTCAAAAGCACTCTCGGAAGCAGTCGGCAACGGCATCGATTCGGCCGCTCAGGCCAGC

CATGCCTACCAGCAAGTTCTGGCCTTCAGCCACCTGTATGACCGTGGCGGCAGCGAGCTG

GTGAACGGCAAGCCATCCTTCACCGCCGACCAGGCGGCGGATTCGATCCTGCGCAAAGGC

CTGTCCTGGCACGACCAGAACGGCGACGGCAAGATCGACCTGAGCTACAGCTTCCTCACC

GAAAAACCGGCCAACTACAACCCGGCCCTGGGCAGCTTCAGCGAGTTCAGCGCACTGCAA

AAAGCCCAGGCGCTGCTGGCTCTGCAATCCTGGGCGGACGTGGCCAACGTGACCTTCACT

GAAGCGGCCAAGGGTGGCGACGGCCACATGAGCTTCGGCAACTACAACGTCAGCACCGGC

GGCGCGGCCTTTGCCTACCTGCCGTCCGGCAGCAGCTACGACGGCCAGTCCTGGTACCTG

ATCAACGACCAGTACCGGGTCAACGAAACCCCGGGCAACGGCAACTACGGCCGCCAGACC

CTGACCCACGAAATCGGCCACAGCCTGGGCCTGTCCCACCCGGGCGTGTACAACGCCGGC

AGCGGCAGCCCTACCTACAACGACGTGACCTACGCCCAGGACACCCGCGGCTACAGCCTG

ATGAGCTACTGGAGTGAGAGCAACACCGGGCAGGACTTCAGCAAGGACGGCGGCGGTGCC

TACGCCTCGGCGCCGTTGCTGGACGATATCGTTGCGGTGCAGAAGCTCTATGGCGCCAAC

CTGGAAACCCGTGCCGACGACACTGTCTACGGCTTCAACTCCAACACCGGTCGGGACTTC

TACAGCGCCCACAGCGCTTCGGACAAGCTGGTGTTCGCGGTCTGGGACGGCGGCGGCAAC

GATACCCTGGATTTCTCGGGTTTCACCCAGAACCAGAAGATCAATCTCAATGAAGGCGGC

TTCTCCGATGTCGGCGGCCTGGTGGGCAACGTGTCCATCGCCCATGGCGTGACCGTGGAA

AACGCCATCGGCGGCTCGGGCAATGACCTGCTGATCGGCAACGCCGCGGCGAACATCCTC

GAAGGCGGTGCCGGCAACGACATCATCTATGGCGGTGGCGGTGGCGACACCCTGTGGGGT

GGGGCGGGGGCTGATACCTTCGTCTACGGCGCGGCCGCGGATTCGACCTTCGACGCCCCG

GACTGGATCATGGATTTCGTCAGCGGCGAGGACAAGATCGACCTCACCGGGATCCCGCAA

TTCGCCTCCGGTGGCGCCACCCTGAACTTCGTCAGCGGCTTCACCGGGCATGCCGGCGAC

GCGATCCTCACCTACTACGCCGAAACCAACCAGACCAGCCTGATGATCGACCTGACCGGT

CATGGCGCGGTGGACTTCGCCGTGGGTACCGTGGGCCAGGCGGCGGTCACCGACATCATC

GCCTGA

>fpvA fig|380021.185.peg.420 Outer membrane ferripyoverdine receptor FpvA, TonB-dependent @ Ferric siderophore receptor, TonB dependent

ATGCACCTTCCAACACTCACCCCGGCCCGCAGCATCCTCGCGCTGGCCATCTGCCTGGCC

TGCAACCCCGTGATGGCGGTCGAGCCCACCACCGCTACCGGCAACCAGGCAGCCGCCACC

TACAGTTTTGCCATCGCCCGCCAGTCCCTGGCCAATGCCCTGGACCAACTGAGTACCCAG

AGCGGTTTGCAGATCGCCTACTCCGCGGCACTGGCCCAGGGGATCGAGTCGGCGGGGGTC

AGCGGGCGCATGAGCGCGGAACAGGCACTGGGCAAGCTGCTGGCAGGCACCGGCCTGGGT

TTTGAACGCAACGGCGCCAACGCGGCGCTGCTGACGCGCCTGCCCCAGAGCAGCCAGGCC

GTGGAGCTGGAGGCCACCCAGATCGTCAGCAACCAGTTGGGCACCGTCACCGAAGGCAGC

GGCTCCTACACCCCAGGCACCATCGCCACGGCGACTCGCATGGTGCTGACCCCGCGGCAG

ACACCGCAGTCGATCACCGTGGTCACCCGGCAGCACATGGAAGATTTCGGCCTCAACAAC

GTCGACGACGTGATGCGCCACACCCCGGGCATCACCGTCTCGGCCTACGACACCGACCGC

ACCAACTACTACGCCCGCGGTTTCTCGATCAACAACTTCCAGTACGACGGCATTCCCTCC

ACCGTGCGCAACGTCGCCTATTCGGCTGGCAACACCCTCAGCGACATGGCCATCTACGAC

CGGGTGGAAGTGCTCAAGGGTGCCACCGGCCTGCTGACCGGCGCCGGCTCCCTGGGCGCC

ACCATCAACCTGGTGCGCAAGAAGCCCACCGCCCAGTTCCAGGGCCACGCCTCCCTGGGC

ATGGGCTCCTGGGACAACTACCGCAGCGAACTGGACGTCAGCGGGCCGCTGACCGAAACC

GGCAATGTTCGCGGCCGCGCAGTGGCGGCCTACCAGGACAAGCAGTCGTTCCTCGACCAC

TATTCGCGCAAGACCTCGACCTACTACGGCATCCTCGAATTCGACCTGTCCCCCGACACC

CTGCTGACCGTGGGCGGCGACTACCAGGACAACATCCCCAAGGGCTCCAGCTGGTCCGGC

ACCTTTCCGCTGATCAACGCCACTGGCGGGCACAACAGCATGTCGCGCTCCTACAACAAC

GGCGCGACCTGGAGCGGCTGGGAGCAATACACCCGCACCGCTTTTGCCATGCTCGAACAC

GACCTGGGGGATGGCTGGGTCACCAAGCTGCAACTGGACCACAAGATCAACAGCTACCAC

GCCGAACTCGGCTCGATCCAGTTCGACGAACCGCAGACCGATGGCACGGCCAAGGTCAAC

GCACAGAAGTACACCGGCGACACCACCAGCGATTCCGCCGACCTGTACCTGAGCGGCCCC

TTCAATCTGTTCGGCCGCGAGCATGAACTGGTGCTGGGCGGCTCCATCGCCAACTCGCGC

TGGACCGGCAAGGGCTACTGGTCCCCGGACTTCCCCGGGGGCAAGGGTAACGTGGTGGAC

TTCTACAACTGGCACGGCAAGATCGAACGGCCCATCTGGGGGCTGCCGGCCCAGCGTACC

GACGACACCGTGCGCCAGACCGGCACCTACATGACCACCCGCCTCAACCTGATGGACGAC

CTGAACCTGTTTCTGGGCGGGCGCGTGGTCAACTATCACCTCACTGGCCTGACCCCGTCC

TACAAGGAAAGCGGACGCTTCGTGCCCTACGTCGGCGCGGTCTACGACCTCGACGACCAC

TTCTCGGTCTACGCCAGCTACACCGATATCTTCATGCCCCAGGAAAGCTGGAACAAGGAC

AAGGACAGCAAGCTGCTCAACCCGGACGAAGGCCAGAACTACGAGCTGGGGCTCAAGGGC

GAGTTCTTCGAAGGCCGCCTCAACAGCAGCCTGGCCTACTTCGAAGTGCACGAGACCAAC

CGCTCGGTGCCGGACGATGCCTACAACAACCAGTCGCCCACCCCCGACAACTACGCCTTC

AAGGGCGCCAAGGCCGTGACCAAGGGGTACGAACTGGAGATTTCCGGCGAACTGAGCCCG

GGCTGGCAAGTGCAGGCCGGCTACACCCACAAGATCGTGCGTGATGACGAGGGCGACAAG

ATCTCCACCTTCGAGCCGCAGGACCAGGTCAACCTCTACACCAGCTACAAGCTCAAGGGC

GACCTGGACAAGCTGACCGTCGGTGGCGGCCTGCGCTGGCAGAGCGAGGGCTGGTATTCG

GTCTACAACGCGCCGCGCAAGATCAACGAAGACATTTCCCAGGAGGCCTACTGGCTGGTG

GACCTGATGACCCGCTACCAGATCACCAAGAACCTGTCGGCGACCCTCAACGTCAACAAC

ATCTTCGACAAGTCCTACTACACCAACGTCGGTTTCTATAACTCGGCGGCCTACGGCGAA

CCCCGCAACTTTATGCTCAGCACCCGCTGGGACTTCTGA

>pchD fig|380021.185.peg.1583 2,3-dihydroxybenzoate-AMP ligase (EC 2.7.7.58) of siderophore biosynthesis

ATGTCCACTTTCGATGACCTCAAGGATTGCCCCTCTTGGCCCGAAGACTTCGCCCAGCGC

TACCGCCAGGCCGGCTACTGGCGCGACGAAACCTTTGGCGACCTGCTGCGCAGCGCCGCC

CAGGCCTTTGCCGAGCGTGAAGCACTGACCGAGGGCGAGCAGCACCTGAGCTACCGGCAA

CTGGACCTGCGGGTCGACCAACTGGCCGCCGGCTTATACCGGCTGGGTCTGCGGGCCGGC

GACAACGTGGTGCTGCAACTGCCCAACAGTGCGGCCTTCGTCGAAGTCTGCTTTGCCCTT

TACCGCCTCGGGGTGCGGCCGATCTTCGCGCTGCCGGCCCACCGGCACCTGGAAATCGGC

CGCTTCTGCGAGTTCGCCCGGGCCCGGGCCTACTTCTGCGCCGACCGGGACGCCAGCTTC

GACTATCGGGCCATGGCCCGCGACCTCAAGGACCGCAACCCGCAGCTGGAGTGGGTGGTA

GTCGCCGGCGAGGCCGAGGAATTCACGGCCTTGCACAGCCTGTATGAGCCGGCGCCTGCG

CGCACCTTCCCGTCCCCCAGCGCCGACGCGGTGGCCTGTTTCCAGCTCTCCGGCGGCTCC

ACCGGGGTGCCCAAGCTGATCCCGCGCCGGCACCACGAATACCTCTACAACCTGCGGGCC

AGCGCCGAGCGCTGTGGCCTGTCCGAGGCCAGCGTGTACCTGGTGGCGCTGCCCATGGCG

CACAACTTTCCCATGTGCTGCCCAGGGTTCATCGGCACCTTTTCGGTGGGCGGGCGGGTG

GTGCTGAGCCCCTCGCCGAGCCCGGAAGTCTGCTTCGAACTGATCGAGCGCCAGGGGGTG

ACCCACACCGCCCTGGTGCCGCCCCTGGCCCTGGTCTGGCTGGAAGCGGCCCAGGCCCGC

GGGCGTGGCCTGGTGCCGTTGCAACTGCTGCAAGTGGGCGGCGCCAAGCTCAGCTACGAG

GCCGCAAGGCGCATCGAACCGGTGCTCGGCTGCCGCCTGCAGCAGGTGTTCGGCATGGCC

GAGGGGCTGATCTGCTACACCGACCCCGAGGACCCGCCACAGCGGGTGCTGCACACCCAG

GGCCGGCCGCTGTCGCCGGCGGACGAAATTCGTGTGGTGGACGAGCACGACCAGCCGGTG

CCGGTGGGGCAGGTGGGGCAGTTGCTGACCCGCGGCCCCTACACCATCCGCGGTTACTAC

CGTTACCCCGAGCACAACGCCCAGGCCTTCACGGCCGATGGTTTCTACCGCACCGGCGAC

CGGGTCATGCTCACTGCCGACGGCTACCTGATGGTGGAAGGGCGCGACAAGGACCTGATC

AACCGCGGGGGCGAAAAGATCGCTGCCGAAGAAGTGGAAAACCTGCTGCTCAGCCACCCG

TCGGTGGCCGACATCGCCCTGGTGGCGATGCCTGATGCCTTCCTGGGCGAACGCACCTGC

GCCTTCGTCATCCCCCGCGGCACCGCGCCCCGGGCCCCGGAGCTGCTGCGTCACCTAAGG

GCCCAGGGCCTGGCGGCGTTCAAGCTGCCGGACCGCTTCGAGTTCATCCCGGCCTTCCCC

CAGACCGGGGTCGGCAAGGTCAGCCGCAAGCACCTGCGCGAGGCGATCCAGGCCCTGTAC

TTCGGCGCCCAGGCCGAACCCCTGGAGGGAAGTGGCGCCCGTGGCTGA

>pchC fig|380021.185.peg.1577 Pyochelin biosynthetic protein PchC, predicted thioesterase @ Thioesterase in siderophore biosynthesis gene cluster

GTGAGAAAGGTTCCCAGTGCCTGGCTGCGTCGTTATCCGCAGCCGCAACCGCCGCGCTGC

CGCCTGGTGTGCCTGCCCCATGCCGGTGGCAGCGCGAGCTTTTTCAACGACTGGCGTGGC

CAGTTGCCGGCGGATGTCGAGCTGGTCAGCGTGCAGTACCCGGGGCGCGAAGAGCGCCTG

AGCGAAAGCTGGCCCGGCAGCCTGGAGTGGATGGCGGGCACTATTACCCGGGCCCTGTCG

GACCTGGTGGACCGGCCCCTGGTGCTGTTCGGCCACAGCATGGGCGCGGCCCTGGCCTAT

GAGGTGGCGGCGCGCATGCAGCAGCAGGGCTCGGCGCCACAACGGTTGATCGTGTCCGCG

CACCCCGCGCCCCATCGCCAGCGCAGCGGCGAACTGCACCTGGGCCCGGACGAGGACCTG

CTGGCGAATGTGCGACGCCTGTCGGACGGTGCGCCGTCGCCCCTGGATGACCCGGCGCTG

CGCGACCTGTACCTGCCGGCGTTGCGCAACGACTACCGGCTGATCGAGTGCTACCGCGGT

GCGCCCGGCCGCGCCCTGGACCTGCCCCTGAGCGTGTGCCTGGGGGCCCGGGACACCGAG

GTGGACCAGGACGAAGCCTATGCCTGGGCCGAGGTCAGCGCCCAGGTCACCGACTTCCAG

GCCTTCCCCGGCGGGCACTTCTACCTGCGCGAGCAACAGGCCGAACTGCTGCGCCACCTG

ACCCGGCTGCTGGCCGGCCATGGCGAGCAGCCGTGGCAGTGCTGGCCTTCGACCCCATGA

>pchB fig|380021.185.peg.1576 Isochorismate pyruvate-lyase

ATGAATTTTCCCTTGGTAGACCCCGACATGAAGACACCCGAGCAGTGCAGCGGCCTGGAC

GACGTCCGCTGCGGTATCGACGCCATGGACCAGCAGATCATCCAGGCCCTGGGCCGGCGC

CTGGCCTACGTCAAGGCGGCGGCGCAGTTCAAGCCGACCGAGGACAGCATTGCCGCCCCG

GAGCGGGTTGCGGCGATGCTCCCGCAACGTCGGCAATGGGCCGAGCAGGCGAGCCTGGAC

CCGATGTTCGCGGTGCCGCTGTTTGCCCAGATCATCCACTGGAACATTGCCCAGCAAGTG

CGCCACTGGCGCCGTCAGCACGGGCTGGATCAAGGAGCCCAAGATGAGTGA

>pvdF fig|380021.185.peg.2160 Pyoverdine synthetase PvdF, N5-hydroxyornithine formyltransferase

ATGACAAAGAAGAATCTGGTGTATGTCTGGTCCCTGCGAAATGCCGCCGCCGACAAGGCC

GGGCAGGCGGTGGCTTACAAGGACCACGAGCGCTACATGAAATCGGTGCTGGAATTTCTG

GTCCAGACGCTGAACCAGACCCCCCTGGGCGAGGCCTACGAACTGGTGGGCGTGGTCTAC

GACGATGACGAGCAGTCGCCACGGGACCAGCAGTTGGTCAGCGACTACGGCTTTGCCTAC

CAGCCGGGCCGCCAGTGGTTGTACCCGGCGGACCTGGAAGTGCAGGGCAAGCGGGTCAAC

GACTTGCTGCTGAGTGTGCCGTCCACCTATCGTCGCCATCCCCGCGGCAGTGCCGAACAC

ATCGCCGGCAAGCAGGATTTCGAACGCCGCCTGCACGACACCCTGGTGGAGCTCAAAGCC

GATATCGTGGTGCTCGACGGCCTGCTGGTGATCCTCGATGAACTGGTGCGCCCGGGAGCG

CCTTTCGCCCGGCGGATCATGAACATCCACCCGGGCATCACCCGCATCGAATCGCCCTAT

GAGCGTCGCGGGGCCTATGCCACCTGGAATGCCCTGTACGGCGCCCGTGGGCAGAAAGTA

GTGGATTGGGCGACGAAGGAAACCGAGCCGACCGCGCCGCTATACCTGACCGGTGCCTCG

TTCCACTACGTGGACAACGGTATCGACTCCGGCGAGGTGCTGCATGACGTGCTCAACACC

GAAATCGGCCCCGAGGACACCATCCTCGAACTGCGCTGGAACAACTTCAACAACAGCCTG

TTCCCGGCCTTGCGCCAGGGCCTGTGGCTGATGCTGGAGAACCTTCGCCAGGCTCAGGCC

GGCAAGGCCCGGGACGTCGCCTGA

>ChiC fig|380021.185.peg.32 Chitinase (EC 3.2.1.14)

GTGGATAAGATCGATTTTGCATTAATCAAGAGTCAGGCCGCAGATGCTGCTTCCCTGATG

CCGAGCATTGCCGGCAAGAAGGTGCTCATGGGCTTCTGGCACAACTGGGCCGCCGGTCAC

AGCGACGGCTACCAGCAGGGGCAATTCGCCAACCTGGATCTGGTGGACGTGCCCAAGGAA

TACAACGTGGTTGCAGTGGCCTTCATGAAGGGCAACGGTATTCCCACCTTCAAGCCCTAC

AACCTTTCGGACGCCGAGTTCCGGCGCCAGGTGGGCGTGCTCAACAGCCAGGGCCGGGCG

GTATTGATCTCCCTGGGCGGCGCCGATGCCCATATCGAATTGCACAAGGGCAACGAACAG

CCCCTGGCCAACGAAATCATCCGTCTGGTGGAAACCTACGGCTTCGATGGCCTGGATATC

GACCTGGAGCAGAGCGCCATCGACTTTGCCGACAACAAGAGTGTGCTGCCTGCCGCCTTG

AAGCTGGTGAAGGATCATTACGCCGGCCAAGGCAAGCACTTCATCATCAGCATGGCGCCT

GAGTTTCCCTACCTGACCAGCAATGGCAAGTACGTGGCCTACCTGCAGGCCCTGGAAGGC

TATTACGACTTTGTCGCGCCGCAGTTCTATAACCAGGGAGGCGACGGGCTCTGGGTGCAG

GAGGCCAATGGCGGGCAGGGCGCCTGGATCGCCCAGAACAATGACGCGATGAAAGAGGAT

TTCCTCTACTACCTGAGCGAAAGCCTGGCCTCCGGTACCCGTGGTTTCACTCGCATTGCC

GCGGACAAGCTGGTGATAGGCCTGCCAAGCAACGTCGATGCCGCGGCCACCGGCTATGTG

ATCGACCCGTCGGTGGTGAGCAATGCGTTCAAGCGCTTGCAGGGCGCCGGTCACGCCATC

AAGGGGTTGATGACCTGGTCGGTGAACTGGGATGCCGGCGTCAGTCGGCAGGGTGTGCCC

TACAACTGGGAGTTTCGTCATCGCTATGCGCCGCTGATCCATGGCGATGGCGGTGAGCCG

GAGCGCCCAGGTGCTCCTGGAAACCTGATGGTACTGGGCACCAGTCGCAGCAGCGTCAAC

CTGAGCTGGGGTGTTTCCGGTAGTGTGCGGCCGGTGGAGTTCTACACCTTGTACCGCGAC

GGCAATGCGGTCGCTCGCACTCCCTCTCCGGGCTTCGAGGATCAGGGGCTGAGTGCGGAC

ACCCAATACAGCTATTTTGTGACCGCTACCGACACTCAGGGCCAGGAGTCGCTCCCCAGC

CGCAGTGTGAGTGCCAGGACCGCAGGCGGTGCGGTCGATCCGAGCTTTCCCGAGTGGCGC

ACCAGCCAGCACTACCTCAGGGAGGATGGAGTGACTTACGAAGGCAATCGTTACCTGTGC

CTCCAGGAGCACACCTCCAACCCGGGCTGGACGCCATCCGTAGCGTTCACCCTGTGGAGC

AAGGTGCTGCAGGAGCGTCATGGCTGA

>cbrD fig|380021.185.peg.1713 ABC-type cobalamin/Fe3+-siderophores transport systems ATPase components

ATGACTCACCGTTTGCACGCCGATGCCGTGACCTTGCGCCACGACGCGCGAATCATTTCC

CAGGGCCTGTCCCTGAGCATTCCCGACGGCTCGTTCACGGTCATTGTCGGGCCCAACGCC

TGTGGCAAATCCACCTTGCTGGCGGCGTTGTCCCGGCTACTGGCACCGGCTGAAGGGCGG

GTGGTGCTCGATGGCAAGGACATCCGCCAACTGCCGGCCCGGGAAGTGGCCCGGCGCCTG

GGCCTGCTGCCGCAAAGCGCCATGGCGCCGGATGGCATCAGCGTCGCCGAACTGGTGGCG

CGCGGGCGCTATCCGCACCAGTCGTTGCTGCGCCAGTGGTCGAAGGAGGATGAGCAGGCG

GTGCGCGGGGCGATGGCCGCGACCCGGGTCGAGGCGCTGGCCGAACGCTTGCTGGACGAG

TTGTCCGGCGGCCAGCGCCAGCGGGTGTGGATCGCCATGGTGCTGGCCCAGGAAACGCCG

ATCCTGCTGCTGGACGAGCCCACCACCTACTTGGATATCGTCCATCAGATCGAGCTGCTG

GAGCTTTTGGCCGAGCTCAACCGCCAGGGCCGGACCATCGTCGCGGTGCTGCATGACTTG

AACCAGGCTTGCCGCTATGCCAGCCACCTGATCGCCCTGCGCGACGGCGCCATCGTCGCC

CAGGGCGCGCCGGCGGCGATCTTCACCGAGGCCCTGGTGGAGCAGGTGTTCGGCCTGGCC

TCGGTGATCATCCGCGACCCGGTCAGCGGCACGCCGTTGATGGTGCCCAAGGGCCGAATC

GACCCGGCCTGA

>gltB fig|380021.185.peg.5302 Glutamate synthase

ATGAAAGCAGGTCTGTACCAACCAGATGAATTTAAGGATAACTGTGGTTTCGGCCTGATA

GCCCATATGCAGGGCGAACCCAGTCATACCCTGTTGAAAACGGCCATTGAGGCCCTGACC

TGCATGACCCACCGCGGTGGGATCAACGCGGACGGCAAGACCGGCGACGGTTGCGGTCTG

CTGATGCAAAAGCCCGACCTGTTCCTGCGCGCAGTCGCCACGGAGCACTTCGGCATCGAG

CTGCCCAAGCAATATGCGGTGGGCATGGTGTTCTTCAACCAGGACCCGGTGAAAGCCGAA

GCCGCTCGCGAAAACATGAACCGCGAGATCCTCGCCGCCGGCCTGCAACTGGTGGGCTGG

CGCAAAGTGCCGATCGATACCAGTGTGCTCGGGCGTCTGGCCCTGGAGCGCCTGCCACAG

ATCGAACAAGTATTCATCGGCGGCCAGGGCCTGAGCGACCAGGATTTCGCCATCAAGCTG

TTCAGTTCCCGTCGCCGCTCCTCGGTGGCCAACGCCGCCGACACCGACCACTACATCTGC

AGCTTTTCCCACAAGACCATCATCTATAAGGGCCTGATGATGCCGGCGGACCTCGCCGCC

TTCTATCCGGACCTGGGTGACGAGCGCCTGCAAACCGCGATCTGCGTGTTCCACCAGCGT

TTCTCTACCAACACCCTGCCGAAATGGCCGCTGGCCCAGCCATTCCGCTTCCTCGCCCAC

AACGGCGAGATCAACACCATCACCGGCAACCGCAACTGGGCCGTGGCCCGGCGCACCAAG

TTCGCCAACGACCTGATCCCGGACCTGGAAGAGCTCGGCCCGCTGGTCAACCGCGTGGGT

TCCGACTCCTCGAGCATGGACAACATGCTGGAGCTGATGGTCACCGGCGGCATCGACCTG

TTCCGCGGCGTGCGCATGATCATTCCGCCAGCGTGGCAGAACGTCGAGACCATGGACGCC

GACCTGCGCGCGTTCTACGAATACAACTCGATGCACATGGAGCCGTGGGACGGCCCGGCC

GGCGTGGTCATGACCGACGGCCGCTACGCCGTCTGCCTGCTGGACCGCAACGGCCTGCGC

CCGGCGCGCTGGGTCACCACCCAGAACGGCTTCATCACCCTGGCCTCGGAAATCGGCGTG

TGGAACTACCAGCCCGAGGACGTGATCGCCAAGGGCCGGGTCGGCCCGGGCCAGATCTTT

GCCGTGGACACCGAGACCGGGCAGATCCTCGACACCGACGCCATCGACAACCGCTTGAAG

TCCCGTCATCCATACAAGCAATGGCTGCGCAAGAACGCTCTGCGCATCCAAGCCACCATG

GAAGACAACGACCACGGCTCGGCCTTCTACGACGTCGACCAGCTCAAGCAGTACATGAAG

ATGTACCAGGTCACCTTCGAAGAGCGTGACCAGGTCCTGCGCCCGCTGGGCGAGCAGGGC

TACGAAGCGGTGGGTTCGATGGGCGACGACACGCCGATGGCGGTGCTGTCCCAGCGCGTG

CGTACCCCCTACGACTATTTCCGCCAGCAGTTCGCCCAGGTCACCAACCCGCCGATCGAC

CCGCTGCGCGAAGCCATCGTCATGTCCCTGGAGATCTGCCTCGGTGCCGAGCGCAACATC

TTCCAGGAGTCGCCCGAGCACGCTTCGCGAGTGATACTCAGCTCGCCAGTGATCTCCCCG

GCCAAGTGGCGTTCGCTGATGAACCTCGATCGCCCGGGCTTCGAGCGCCAGGTCATCGAC

CTCAACTACGACGAGAGCGTCGGTCTGGAAGCGGCGATCCGCAACGTCGCCGACCAGGCT

GAAGAAGCCGCGCGCGCCGGCCGCACCCAGATCGTCCTCAGCGACCGTCATATCGCGCCG

GGCAAGTTGCCGATCCACGCCTCCCTGGCCACGGGCGCGGTGCACCACCGCCTGACTGAA

AAGGGCCTGCGCTGCGACTCCAACATCCTCGTGGAAACCGCCACCGCCCGCGATCCGCAC

CACTTCGCGGTGCTGATCGGGTTCGGCGCCTCGGCGGTCTATCCGTTCCTGGCCTACGAA

GTGCTGGGCGACCTGATCCGTACCGGTGAAGTGCTGGGCGACCTCTACGAGGTGTTCAAG

AACTACCGCAAGGGCATCACCAAGGGCCTGCTGAAGATCCTGTCGAAGATGGGCATCTCC

ACCATCGCCTCTTACCGTGGCGCGCAGCTGTTCGAAGCCATCGGCCTGTCCGAGGAGGTC

TGCAACCTGAGCTTCCGTGGCGTGCCGAGCCGGATCAAGGGCGCACGTTTCGTCGACATC

GAAGCCGAGCAGAAAGCCCTGGCGACCGAGGCCTGGAGCCCGCGCAAGCCGATCCAGCAA

GGCGGCCTGCTGAAGTTCGTCCACGGCGGCGAATACCACGCCTACAACCCGGACGTGGTC

AACACCCTGCAAGCCGCTGTGCAGCAGGGCGACTACGCCAAGTTCAAGGAATACACCGCG

CTGGTGGACAACCGCCCGGTGTCGATGATCCGCGACCTGTTCCAGGTGAAGACCCTGGAC

ACGCCGATGGACATCAGCGAAGTCGAGCCCCTGGAGTCGATCCTCAAGCGCTTCGACTCT

GCCGGTATCTCCCTGGGTGCCCTGTCGCCGGAGGCTCACGAAGCCCTGGCCGAAGCCATG

AACCGCCTCGGCGCGCGTTCCAACTCCGGTGAGGGCGGCGAAGACCCGGCGCGCTACGGC

ACCATCAAGAGCTCGAAGATCAAGCAAGTGGCCACTGGACGTTTCGGCGTGACCCCGGAA

TACCTGGTCAACGCCGAAGTGCTGCAGATCAAGGTGGCCCAGGGCGCCAAGCCCGGCGAG

GGCGGCCAGCTGCCTGGTGGCAAGGTCAACGGCCTGATCGCCAAGCTGCGCTACGCAGTC

CCCGGCGTGACCCTGATTTCGCCTCCACCGCACCACGACATCTATTCGATCGAAGACTTG

TCGCAGCTGATCTTCGACCTCAAGCAGGTCAACCCTCAGGCGCTGGTGTCGGTGAAGCTG

GTGGCCGAAGCCGGTGTTGGCACCATCGCTGCCGGTGTGGCCAAGGCCTATGCCGACCTG

ATCACCATCTCCGGCTACGACGGCGGCACCGGCGCTTCGCCGCTGACCTCCATCAAGTAC

GCCGGTGCGCCGTGGGAACTGGGCCTGGCGGAAACCCACCAGACCCTGCGCGGCAACGAC

CTGCGCGGCAAGGTCCGGGTGCAGACCGACGGCGGCCTGAAAACCGGCCTCGACGTGATC

AAGGCCGCCATTCTCGGCGCCGAGAGCTTCGGCTTCGGCACCGCGCCGATGATCGCCCTG

GGCTGCAAGTACCTGCGCATCTGCCACCTGAACAACTGCGCCACCGGTGTGGCGACCCAG

AACGAGAAACTGCGCAAGGATCACTACATCGGCACCGTCGACATGGTGGTGAACTTCTTC

ACCTACGTCGCCGAGGAAACCCGTGAGTGGCTGGCCAAGCTTGGCGTGCGTTCCCTGGAG

CAACTGATCGGCCGGACCGACCTGCTGGAGGTGCTGGAAGGCCAGACCGCCAAGCAGCAT

CACCTGGACCTGACCCCGTTGCTGGGCAGTGACCACATTCCGGCGGACAAGCCGCAGTTC

TGCCAGGTGGACCGCAACCCGCCGTTCGACAAGGGCCTGCTGGCCGAGAAAATGGTCGAG

ATGGCCCGCTCTGCGATCAATGACAAGAGCGGCGCCGATTTCGCCCTGGATATCTGCAAC

TGCGACCGCTCCATCGGCGCACGGATCTCCGGCGAGATCGCCAGGCTCCACGGCAACCAG

GGCATGGCCCAGGCGCCTATCACGTTCCGCTTCAAGGGCACTGCGGGGCAGAGCTTCGGC

GTGTGGAACGCCGGCGGCCTGAACCTCTACCTGGAAGGCGACGCCAACGACTACGTGGGC

AAAGGCATGACCGGCGGCAAGCTGGTGATCGTTCCGCCCAAGGGCAGCGTCTACAAGACC

CAGGACAGCGCCATTATCGGCAACACCTGCCTGTACGGCGCCACCGGCGGCAAGCTGTTC

GCCGCTGGCACCGCGGGTGAGCGTTTCGCCGTGCGCAACTCCGGTGCCCACACCGTGGTG

GAAGGCACTGGCGATCACTGCTGCGAGTACATGACCGGTGGTTTCGTCTGCGTCCTGGGC

AAGACCGGTTACAACTTCGGCTCAGGCATGACCGGCGGTTTCGCCTATGTGCTGGACCAG

GACAACACCTTCGTTGACCGGGTCAACCACGAACTGGTGGAAATCCAACGGATCAGCGGC

GAAGCGATGGAAGCCTATCGCAGCCACCTGCAGCGCGTGCTGAACGAGTACGTCGAGGAA

ACCGACAGCGAATGGGGCCGTAACCTCGCCGAGAACCTCGACGACTACCTGCGCCGTTTC

TGGCTGGTCAAGCCCAAGGCTGCCAGCTTGAAGTCGTTGCTTTCCAGCACCCGTGCCAAC

CCGCAGTGA

>gltD fig|380021.185.peg.5303 Glutamate synthase

ATGGCTGAACGTCTGAATAACGACTTCCAGTTCATCGATGTCGGGCGCAAGGATCCGAAG

AAGAAACTGTTGCGTCAACGCAAGAAAGAGTTCGTGGAAATCTACGAACCCTTCAAACCC

CAGCAGTCGGCCGACCAGGCCCACCGCTGCCTGGGTTGCGGTAACCCATACTGTGAATGG

AAGTGCCCGGTGCACAACTTCATTCCCAACTGGCTCAAGCTGGTGGCCGAGGGCAACATC

CTCGCCGCCGCCGAGCTGTCGCACCAGACCAACACCCTGCCGGAAGTCTGCGGCCGGGTG

TGCCCGCAGGATCGTCTGTGCGAGGGTGCCTGCACCCTCAACGACGGCTTTGGCGCGGTG

ACCATCGGTTCGGTGGAGAAGTACATCACCGACACCGCCTTCGCCATGGGCTGGCGCCCG

GACATGTCCAAGGTCAAGCCCACCGGCAAACGCGTGGCGATCATTGGTGCGGGCCCGGCG

GGCCTGGGCTGTGCCGACGTGCTGGTGCGTGGCGGCGTGACCCCGGTGGTGTTCGACAAG

AACCCGGAAATCGGCGGTCTGCTGACCTTCGGTATCCCCGAGTTCAAGCTGGAAAAGACC

GTGCTGAGCAATCGTCGCGAAGTCTTCAGCGGCATGGGCATCGAGTTCCGCCTGAACACC

GAGGTGGGCAAGGACGTGACCATGGAGCAACTGCTCGCCGAATACGATGCGGTGTTCATG

GGCATGGGCACCTACACCTACATGAAGGGCGGCTTTGCCGGTGAGGACCTGCCGGGCGTC

TATGACGCGCTGGATTTCCTGATCGCCAACGTCAACCGCAACCTGGGCTTTGAAAAGTCG

CCGGAAGACTTCGTCGACATGAAAGGCAAGAAGGTGGTGGTGCTGGGCGGTGGCGACACC

GCGATGGACTGCAACCGCACCTCGATCCGCCAGGGCGCCAAGTCGGTGACCTGCGCCTAT

CGTCGTGACGAAGCCAACATGCCCGGCTCGCGCAAAGAGGTGAAGAACGCCAAGGAAGAA

GGCGTGAAATTCCTCTACAACCGCCAGCCGATCGCCATCGTCGGTGAAGACCGCGTCGAA

GGCGTGAAGGTGGTCGAGACCCGTCTCGGCGAGCCGGACGCCCGTGGCCGCCGCAGCCCC

GAGCCGATCCCGGGTTCTGAAGAGATCATCCCGGCCGACGCCGTGGTCATCGCCTTCGGT

TTCCGTCCAAGCCCGGCGCCATGGTTCGAGCAGTTCAGCATCCAGACCGACAGCCAGGGC

CGTGTCGTGGCCCCGGAACAGGGCCAGTTCAAGCACCAGACCAGCAACCCGAAGATCTTT

GCCGGTGGTGACATGGTGCGCGGTTCTGATCTTGTGGTGACGGCGATCTTCGAAGGGCGT

AACGCCGCCGAAGGCATCCTCGACTACCTGGGCGTCTGA

>gltP fig|380021.185.peg.4948 Proton/glutamate symporter @ Proton/aspartate symporter

ATGAAGAAGGCAAAATTAAGCCTCGCCTGGCAGATCCTCATCGGTCTGGTCCTGGGGATT

GCGATTGGCGCGCTGCTCAATCATTTCAGTGCTGAAAAGGCCTGGTGGATCAGCAACGTC

CTGCAGCCTGCGGGCGATATCTTTATCCGCTTGATCAAGATGATCGTGATCCCGATCGTC

ATTTCCTCGCTGATCGTCGGCATCGCCGGTGTCGGTGACGCGAAGAAACTGGGGCGCATC

GGCCTCAAGACCATCATCTACTTCGAAATCGTGACCACCATCGCCATCGTCGTCGGCCTG

CTGCTGGCCAACCTGTTCCATCCGGGCGCAGGCATCGACATGAGCACCCTGGGCACCGTG

GACATCTCCAAGTACCAGGCCACTGCGGCCGAGGTGCAGCATGAACACGCGTTCATCGAG

ACCATCCTCAACCTGATTCCATCGAACATCTTCGCGGCCATGGCCCGCGGCGAGATGCTG

CCCATCATCTTCTTCTCGGTGCTGTTCGGCCTGGGCCTGTCGAGCCTGCAGTCCGACCTG

CGCGAACCGCTGGTGAAGATGTTCCAGGGCGTTTCGGAAAGCATGTTCAAAGTCACCCAC

ATGATCATGAACTACGCCCCTATCGGCGTATTTGCACTGATCGCGGTGACCGTCGCCAAC

TTCGGTTTCGCCTCCCTGCTGCCGCTGGCCAAGCTGGTGATCCTGGTGTACGTGGCTATC

GCCTTCTTCGCTTTCGTGATCCTCGGCCTGATCGCCCGCCTGTTCGGCTTCTCGGTGATC

AAACTGATGCGCATCTTCAAGGATGAGCTGGTCCTGGCCTACTCCACCGCCAGCTCGGAA

ACCGTGCTGCCGCGGGTGATCGAGAAGATGGAAGCCTACGGCGCGCCGAAAGCCATCTGC

AGCTTCGTGGTGCCGACCGGCTACTCGTTCAACCTCGACGGTTCGACCCTGTACCAGAGC

ATCGCGGCGATCTTCATCGCCCAGCTGTACGGCATCGACCTGTCCATCAGCCAGCAACTG

CTGCTGGTGCTGACCCTGATGGTCACCTCCAAAGGTATCGCCGGGGTTCCGGGCGTGTCC

TTCGTGGTCCTGCTGGCCACCCTGGGCAGCGTCGGCATTCCTCTGGAAGGCCTGGCCTTC

ATCGCCGGTGTCGACCGTGTGATGGACATGGCGCGTACCGCCTTGAACGTGATCGGCAAC

GCCCTGGCGGTACTGGTCATCTCCCGTTGGGAAGGCATGTACGACGACGCCAAGGGCCAG

CGTTACTGGAACTCCCTGCCGCACTGGCGCAGCAAGGAAAAACTGCCAGCAGGCGAAGCG

TCCAAAGGCTAA

>gltS fig|380021.185.peg.5199 Sodium/glutamate symporter

ATGCCGACTTTTGAATTGGACGCGCTGACCACCCTGGCGCTGGCCCTGATCCTGCTGGGG

GTGGGAGCCCAGCTGAAGAAACGCAGCCGCTGGCTGACCCAGCTCTGTGTCCCGGCGCCG

GTGATCGGCGGTTTCGGCTTTGCCCTGATCGTCTGGCTGCTGCGCGATCAGCAACTGCTG

GCGATCAAGCTCGACACCGCGATCCAGACGCCCTTGATGGTGGCGTTCTTCACCACGGTG

GGCCTGGGGGGCAGCCTCGGCCTGCTGCGCCGTGGCGGCAAGATCCTGTTCGTCTACCTG

GGGGCCTGCTGGTCCCTGGCGCTGCTGCAGAACCTGGTGGGGGTCGGTGCCGCCAAGGCG

CTGGGCATCGACCCGCTGCTGGGCATCATGGCCGGTGCGGTGTCCCTGGAGGGTGGCTTT

GGCGCGGCGGCGGCCTTTGGCCCGATCGCGGAAAACCTCGGGGCGGTGGGGGCCACCACG

GCGGCCCTGGCCTCGGCCACCTTCGGCATGGTTGCCGGCGGCTTGCTGGGCAGCCCGCTG

GCGCGCTGGCTGATCGAGCGCAACCGCCTGCAGATCCAGGCCGATCAGGTCAGTACCCTG

CAGCACCTGGAAAGCGCTGCCCAGGGTTCGGTGGCGCCTTTGGATGCGCCGACCCTGCTG

CGCCTGCTGACCTGCATTCTGCTGATCATGGTGCTGGGTTTCTGGATCGGCAGCGCGCTG

AACGAGCACCTGGGCATCGTCCTGCCAAGCTACGTGGGGGCGATGTTCGTGGCGATCATC

CTGCGCAATCTCAATGACCGGGCGCGGATCATCGACATCCCCGACAGCGCCGTGAGCACC

CTGGGCGACGTATGCCTGGGGGTGTTCCTGACCATGGCCATGATGAGCCTGAAGTTCTGG

GAACTGGAACAGCTCGGCCTGCCGCTGCTGGTGATCCTGGTGGTGCAGGTGCTGGTGATG

GTGCTGCTGTGCGTATTCCTGCTGTTCCGCCTGTTTGGCGGCAACTACGACGCGGCGGTG

TTGTGTGCCGGCTTCATGGGCCACGGCCTGGGGGCCACGCCCAATGCGGTGGCCAACATG

GGCGCGATCTGTGATCACTACAAGGTCTTTTCCTACAAGGCCTTCATCATCGTGCCGCTG

TGCGGTGCGGTATTGATCGACATCGTGGCGATCCCGCTGATCACCTGGTTCATCAACGCC

TTTGCCTGA

>gltT fig|380021.185.peg.4948 Proton/glutamate symporter @ Proton/aspartate symporter

ATGAAGAAGGCAAAATTAAGCCTCGCCTGGCAGATCCTCATCGGTCTGGTCCTGGGGATT

GCGATTGGCGCGCTGCTCAATCATTTCAGTGCTGAAAAGGCCTGGTGGATCAGCAACGTC

CTGCAGCCTGCGGGCGATATCTTTATCCGCTTGATCAAGATGATCGTGATCCCGATCGTC

ATTTCCTCGCTGATCGTCGGCATCGCCGGTGTCGGTGACGCGAAGAAACTGGGGCGCATC

GGCCTCAAGACCATCATCTACTTCGAAATCGTGACCACCATCGCCATCGTCGTCGGCCTG

CTGCTGGCCAACCTGTTCCATCCGGGCGCAGGCATCGACATGAGCACCCTGGGCACCGTG

GACATCTCCAAGTACCAGGCCACTGCGGCCGAGGTGCAGCATGAACACGCGTTCATCGAG

ACCATCCTCAACCTGATTCCATCGAACATCTTCGCGGCCATGGCCCGCGGCGAGATGCTG

CCCATCATCTTCTTCTCGGTGCTGTTCGGCCTGGGCCTGTCGAGCCTGCAGTCCGACCTG

CGCGAACCGCTGGTGAAGATGTTCCAGGGCGTTTCGGAAAGCATGTTCAAAGTCACCCAC

ATGATCATGAACTACGCCCCTATCGGCGTATTTGCACTGATCGCGGTGACCGTCGCCAAC

TTCGGTTTCGCCTCCCTGCTGCCGCTGGCCAAGCTGGTGATCCTGGTGTACGTGGCTATC

GCCTTCTTCGCTTTCGTGATCCTCGGCCTGATCGCCCGCCTGTTCGGCTTCTCGGTGATC

AAACTGATGCGCATCTTCAAGGATGAGCTGGTCCTGGCCTACTCCACCGCCAGCTCGGAA

ACCGTGCTGCCGCGGGTGATCGAGAAGATGGAAGCCTACGGCGCGCCGAAAGCCATCTGC

AGCTTCGTGGTGCCGACCGGCTACTCGTTCAACCTCGACGGTTCGACCCTGTACCAGAGC

ATCGCGGCGATCTTCATCGCCCAGCTGTACGGCATCGACCTGTCCATCAGCCAGCAACTG

CTGCTGGTGCTGACCCTGATGGTCACCTCCAAAGGTATCGCCGGGGTTCCGGGCGTGTCC

TTCGTGGTCCTGCTGGCCACCCTGGGCAGCGTCGGCATTCCTCTGGAAGGCCTGGCCTTC

ATCGCCGGTGTCGACCGTGTGATGGACATGGCGCGTACCGCCTTGAACGTGATCGGCAAC

GCCCTGGCGGTACTGGTCATCTCCCGTTGGGAAGGCATGTACGACGACGCCAAGGGCCAG

CGTTACTGGAACTCCCTGCCGCACTGGCGCAGCAAGGAAAAACTGCCAGCAGGCGAAGCG

TCCAAAGGCTAA

>hfq fig|380021.185.peg.5418 RNA-binding protein Hfq

ATGTCAAAAGGGCATTCGCTACAAGACCCTTACTTGAATACATTGCGTAAAGAGAAGGTT

GGGGTTTCCATCTATCTGGTCAACGGGATCAAGCTGCAAGGTACGATCGAGTCTTTCGAC

CAGTTCGTCATCCTGCTGAAGAACACCGTCAGCCAAATGGTTTACAAGCACGCTATCTCG

ACAGTGGTTCCAGTTCGCCCGATTCGCCTGCCTAGCGCTTCCGAATCCGAACAGGGTGAC

GCTGAGCCAGGTAACGCCTGA

>pltC fig|380021.185.peg.840 Polyketide synthase modules and related proteins

ATGGATAACGATGTCCGCGATGTAAGCAAGGAACAGCTGCAAGAGAGTCTTGCTCAGGCA

ATCACCACCATCCGTGCGCTCAAGGAAAAGGTGGCCGGCAAGAGCTCGGCGCCTGTCGAA

CCGATCGCCGTAGTGGGCCTGGGGTGCCGGCTGCCGGGCAGTGCCGACACACCGAAGCGG

CTGTGGAGCCTGCTGAAAAACGCCACCGATGCGGTGGGCGACATGCCCAGCGACCGTCTG

TACGGCACCGACTATTACCATCCTGATCCCCAGGCACCCGGCAAGGCCTACGTCATGCGC

GGTGGCTTCATCGAGGGGGTGGATCAGTTCGACCCGGGCTTCTTCGGCATTTCGCCCAAG

GAAGCCGAAGGCATGGACCCCCAGCAGCGCCTGGCCCTGGAGGTTGCCTGGGAGGCCCTG

GAGAACGCCGCGATCGCCCCCGACAGCCTGCATGGCAAGAAGCTCGGCGTGTTCATGGGG

GTCAGTACCAATGATTACGTGCGCCTGCGCCAGCAGTTGGGCGCGGTCGAGGACATCAAC

GCCTACCAGTTCTATGGCGAAACCAGCTTCGTGGCCGGGCGCATTGCCTACACCCTGGGC

TCCAGGGGCCCGGCGGTGGTGCTCGACACCTCCTGCTCCTCATCCCTGGTGGCCCTGCAC

CAGGCCTGCAACAGCCTGCGCAGCCGCGAGAGCGAGCTGGCGCTGGCCGGTGGGGTCAAC

CTGATCCTGTCGCCCTACGGTTTCATCCTGGTCAGCAAGCTGCGGGCCGTGGCCCCCGAT

GGCCGCTGCAAGACCTTCGACGCGGCGGCCGATGGCTACGGGCGCGCCGAAGGCTGCGTG

ATCCTTGCGCTCAAGCGGCTGAGCGATGCGGTACGCGACCAGGACCCGGTGCTGGCCGTG

ATCGAGGGTAGTGCGGTCAACAACGACGGCGCCAGCAGCGGCATCACCGTGCCCAACATC

CACGCCCAGGAAGAGGTGATCAGGCTGGCGCTCGGCCAGGCCGGGCTCCAGGGCAGCGAG

GTCGACTATGTCGAGGCCCATGGCACCGGCACCGCGCTGGGCGACCCGATCGAACTGCAC

GCCCTGCATGCGGTACTGGGCAAGCAGCGGCCCGTGGATGCACCGCTGCTGGTGGGCTCG

ATCAAGGCCAACATGGGGCATCTCGAACCGGTCGCCGGGGTGACCGGGCTGGCCAAGGTC

CTGCTGTGCCTGCAACAAGAGGCCCTGGTGCCCCAGGTGCACTTCAACACGCCCAACCCG

CGGATCGAATGGGATCGCCTGGCCCTGAAGGTGGTCACCGAATCCACGCCCTGGCCACGC

CAGGGCAAGGCGCGGCACGCCGGTGTCAGTTCGTTTGGTGTCACCGGCACCAACGCCCAT

GTGCTAGTGGGCGACGCGCCGCTGCGCGAACGTGCCCAGGGGCGCGACAACCCCTGGCAG

CTGATCACTCTGTCGGCCAAGGGCGAGACGCCCCGGCGCCAGATTGCCGGACGCTATGAA

CGTTTTATCGCCGACAACAGCCAGCTCGAACTCAAGGACCTGTGCTACACGGCGAACGTC

GGGCGGGCGCACTTTGGCCATCGTTTCGCGGCCGTGGCCGATAGCCGTGAGGGGCTGCGC

GAGCAACTGGCAGCCTATGCGTCACGCAAGGTGGTGGGGCATGTATTCGAAGGGCGCTGC

CAGGGAGCGGCGGCGCCGCTGGTGATGCTCTTTCCGGGGCAGGGCTGCCAGTACCGGGCA

ATGGCCCAGGCACTGTATGACAGCGAACCATTCTTCAAGGCGCAGATCGATGAATGCCGC

GCCCTGTTGCAGCCGCTGATGGACGTGGACCTGCTGACCCTGGTGCTGGACGCGGGTGCG

GCCAGTGACAGCTACCTGCAACAGACCCGTTATGCCCAGCCGGCGATATTCGCGGTCGAA

TATGCCCTGGCGCGGTTGTGGATGCATTGGGGGGTCGCTGCCGATGCGCTGTTCGGACAC

AGTTTCGGCGAGATCGGTGCAATCTGTGTGGCCGGGGCGGTATCCCTGGCTGACGCGCTG

CGTATGGTGGAGGCGCGTGGGCGCCTGGCCCAGCAACTGATGACGGCCGGCGGCGCGATG

TACGCACTGGGCATGAGCGAGGCGCAACTGCAGGAGCTGCTCAAGGACCGGCCCGGCAGC

GCGATCGAACTGGCGGCGGTCAACAGCCCGCAGGACGTGGTGGTGGCCGGGCCGCAAGCC

GAGGTCCAGGCGCTGGCCGAAGCGGCACTCGCCAGCGGTTGCAAGGTCAAGAAGCTTGCG

GTTTCCCATGCCTTCCATACCGCAGCCACCGAGCCGATGCTTGAAGCGTTCCGTCAGACG

GTGGCGCAGATCACCTTCAGCGAGCCGCGCTTGCCGGTCATCAGCAGTGTCACTGGCCGG

GTGCATACGCTCTCCAGCCTGAGCTCGCCCGATTACTGGTGTACGCACACCCGCCAGGCC

GTGAGGTTCAGCGAAGGGGTCAACACCCTGATCGCAGAGCTGGGGGTGAAAACCTTCCTT

GAAGTGGCCTCCGATGCTGTGTTGACGCCGCTGATCGGCCGTCACCCCCTGGCGGACGAC

AGCCTGGTCCTGGCCAGCCTGCGCCGGGTCGGCGATCCGTCCAGGGACCTGCGCCTGGCC

GCCGCGCAGCTCTACGTGGGCGGCCATAACCTCGACTGGGCCCGGCTGCACGAGCATGAC

GGGGCCTTGCGCCAGGCCTTGCCCGGGTATGCGTTTCAGCGCCAGCGCTACTGGTTCGAC

AACGCCAGCGGCTCGCCCCTGCAACAGGTCGCTGCGGGAGCGGTCGGCCGGCTGCTGGGG

CATTCGGTCAATGCACCCACTCCGGCATTCGAGTCGACGCTCGATAGCGCGCTCCTGCAG

GCCGTGGGCGGCGAGATCCGCGATGACCTGGCGCTGCTGCGTCCTGACCGGTTGCTGGCA

GCCCTGAGCGATGAGCTGGCCGGCCACTTGCAGCTGGACACCTATGGGGTGAGCCTCGCC

AGCATCACCCCGGCCCTGGCGTTCCATGTCGATGACCAGTTGCATCTGTTCACCGAACTC

AAGCCGCTGTCCGGGGCGGCCTGGGAGGTCAACTGCTCGGCCCTGAGCGCAGCGGCCAAG

GTTGCCGGTGCCGATTGGCAGCCGGTCCTGTCGCTGACCCTGGAAGGCCTGCCGGCGGCT

GTCCGGGGCGGCCTGCCGGACCCGGGCCATGCCGCCGCCCAGGACGCAGGCTTTGTCTAC

CACATGCAGTTGCCGGCAGAACCCGATCCGCACGGCAATCACCTTGGGCAGGTCCTCGAG

TACCTGGGGCAGGCGGTGCCGGGCGATGCGCCGGGCTCGATAAGCGGCATCCGCCGCTGG

GTCGCCAGCCGGTCCGCTTCGCCCCGGGCACAAAGCCTGGTGATTGCCACGAGCCAGGCA

CATCCGCAGCAGTTCGACTGCGCCTTGTACGATGCGCACGGCCAGTGTGTAGGCAGCCTG

GAAGGGCTGACCCTGGCTGCCGCCCCGAGTGAGGAGGCGCTGCGTGGCATGCTTTACCAG

CCCGACGTGCTCTACAGCCTGGATTGGCTCGAACGCCCTCGCCAGCGCGCCGAAGTGCCG

CAGCAGGGCCGCTTGTTCACCCTGGTCAGCCGCTCGCCGGAGACCGCCGCTCCCCTGGTC

GAACACCTGCAGCGTGGCGGGCATCGTGCCCGGGTCCTGTGCCCGCAGCAGTTGCTCGAT

AGCGCGCGGAGGGTGCTGGCCCAGGACCCGAGCCAGGCATTGACCGATGAAGGCGAGGCC

TTGGCCGCCGATATCATCGTGCTCGATGGCAAAGACATCGAGGATGCCGGCTCGACCACG

CTGCAGAGCCTGTCGAGCCTGCAGGCGACGCTGTTCCACCCGTTGCTGGAGATGGTCCAG

GCGCTCATCGAACTGGGCCCGCGCGGTGGCCGGTTGTGGCTGGTGACCCAAGGGGCAAAC

GCCGTTGGCCTGGATCGCGACCAGCCCTTGCAAGTGGCGACCGGGCCGTTGTGGGGGCTG

GGCAAGACGCTCGCCCTGGAGCACCCCGAACACTGGCGCGGGCTGATCGACCTGACCCCG

GACGATCCTCACTGGGCAAGGGCGCTGGCCGAAGAAGTCAGCGACCCCGATGGCGAGGAC

AAGATCTGCCTGCGCCCGGGCAAGCGTTATGTCCAGCGCCTGAACCATTTCAGCGCTACG

CAGTTGCCAGCACAGGCCTATGCACCTTGCCCGCAGGGCAGTTACCTGATTACCGGCGGC

ATGGGTGGAATTGGCCTGGCCATGGCCCAGTGGCTTCTGGATAAAGGCGCGGGCGCTGTG

CTGATCACCGGCCGGCGGCCACTGGAGGATGTCGCCACGGGCCTGGAGCGCTTCGGCGCT

GCGGCATCGCGGGTGAGTTACGTCCAGGCCGACATCACCTCGCCCCAGGACATGCAGCGG

CTGTTCTGCGGTCTGGAGGCGTCGGGCGCGAGCCTGAAGGGCATCTTCCATGCCGCCGGG

ATTTCAATTCCGCAGGATCTCAAGGACGTCGACCGTGACAGTTTCGACCAGGTGATGCGG

CCCAAGGTCGAGGGCACCTGGTTGTTGCACGAACTGTCCCTGGGGCTGGACCTGGACTTC

TTTGTCCTGTGTTCGAGCATCGCCAGTGTCTGGGGTTCGCAGCATGTCGCCAGCTACGCA

GCCGCCAATCAGTTCCTCGACAGCCTGGCGTGGCAGCGTCGGGCCATGGGCCTGAGTGCC

CTGGTGATCGACTGGGGGCTGTGGGCCGGCGGCAGTCACCTGTTCGACGAGCAGGTGCTG

AATTTCCTCACCAGCGTTGGCTTGAAACAGATCGCCCCGGTACAGAACGTCGGCCTGCTG

TCGCGGATTCTGGCCAGCGAGCTGCCCCAGATGGTGGTGTCAGGGGTGGACTGGAATCGC

TTCAAGCCACTGCTCGAATCCCGCGGCCCGCAACCGCTGCTGCAGTACATCCGCAGCCAG

GCTCCGACCGCCAGGGCCGGCGACAGCAGCAACGTGGAGATCCTGCAGCAACTGGCGGGC

GCCGACGAAGCCGCTGCCCTGGCATTGCTGGATGACTACGTGTGGGAGCAGTACGCGCAG

TTGCTCGGGGTCAAGACCGAACAGGTGCGGGCCAAGCTCGAGGATGGCGGCAGCCTGATG

GACTACGGCCTGGACTCGCTGCTGGTGATGGACATGGTCGCCCGCTGCCGGCGCGATCTG

AAGCTGGAGATCAAGGCCCGCGAGTTTCTTGAGTGTCCGGGCCTGATGTGGCCGGACTTC

CTGGCCCGTTCGATAAAGGAACAGGGCTGCGTGGCCGAGGCCTGA

>pltD fig|380021.185.peg.841 hypothetical protein

ATGAACGATGTGCAGTCTGGCAAGGCGCCAGAGCATTACGACATTCTCTTGGCGGGCAAC

AGCATCAGCGTGATCATGCTCGCCGCCTGCCTGGCCCGGAACAAGGTCCGGGTCGGTTTG

TTGCGCAACCGGCAGATGCCCCCCGACCTTACCGGTGAGGCGACGATTCCCTATACCTCG

ATGATTTTCGAGCTGATTGCCGACCGCTATGGCGTGCCGGAAATAAAGAATATCGCCCGC

ACCCGGGATATCCAGCAGAAGGTGATGCCGTCTTCCGGGGTCAAGAAGAACCTCGGGTTC

ATCTATCACCAGCGCAGCCGGGCGGTGGACCTGGGCCAGGCGCTGCAATTCAACGTGCCC

TCCGAGCATGGCGAGAACCATCTGTTCAGGCCCGATATCGATGCCTATCTGCTGGTGGCG

GCCATCGGTTATGGCGCGCAACTGGTGGAAATCGACAACAGCCCCGAGGTGCTGGTCGAG

GACAGCGGGGTCAAGGTAGCTACGGCACTGGGGCGCTGGGTCACTGCCGATTTCATGGTT

GATGGCAGCCAGGGCGGCCAGGTGCTGGCGCGGCAGGCGGGCCTGGTCAGCCAGGCTTCG

ACGCAGAAGACCCGGACCCTGGAATTCTCCACTCATATGCTCGGGGTGGTGCCGTTCGAT

GAGTGCGTGCAGGGCGATTTTCCCGGCCAGTGGCATGGCGGCACTCTGCATCACGTGTTC

GACGGGGGCTGGGTGGGGGTCATCCCGTTCAACAACCATCAGCACTCGCGCAACCCTTTG

GTCAGCGTGCTGGTTTCACTGCGTGAGGACCTCTGCCCGAGCATGGACGGCGACCAGGTC

CTGGCCGGCCTGATCGAGCTGTACCCCGGCCTGGGGCGGCACCTGTCCGGCGCCCGGCGG

GTGCGCGAGTGGGCGCTGCGCCAGCCGCCCCGGCAGGTCTATCGCACGGCACTCGAACGC

CGCTGCCTGATGTTCGATGAGGGCGCCGCGAGCAACGATCTGTTGTTCTCGCGCAAGCTG

TCCAATGCTGCGGAACTGGTTCTGGCCCTGGCGCACCGGCTGATCAAGGCGGCGCACAGC

GGTGACTACCGCAGCCCGGCCCTGAATGATTTTGTCCTGACCCAGGACAGCATCATCAGC

TTGAGTGACCGGATCGCCTCAGCGGCCTATGTGTCGTTTCGCGACCCCGAGTTGTGGAAT

GCCTTCGCCCGTGTCTGGCTGCTGCAGTCGATTGCCGCCACCATCACCGCGCGCAAGATC

AACGATGCCTTTGCCAAGGACCTGGACCCGCGGGTGTTCGATGAAATCGACCAGCTCGCA

GAGGACGGTTTCTGGATGCCTCTGTATCAGGGGTACAAGGATATTCTCAACACTACGCTG

GGCCTTTGTGATGACGTCAAAAGCGCCAAGGTCTCTGCTGCGCACGCGGCGAGCAGCATC

TTTGCGGAGCTTGCCAACGCCAGTTTTGTTCCGCCTATTTTTGATTTTGCTAATCCTCAC

GCTCGTGTCTATCAACTGACCACCTTGAGAAAGCTCAAGGCGCTCTGGTGGGGCCTGATG

CAAGTGCCCTCAGAGGTCGGGCGGCTGATTTTCTATCGATCCTTCAGAAAACCTTCCCTG

CGCAAGGAGAGTTGA

>pltE fig|380021.185.peg.842 Butyryl-CoA dehydrogenase (EC 1.3.99.2)

ATGGACTTCAACTACGACGATACCCAGAAAAAACATGCGGCCATGATCGCCCAGGTGTGT

GCCGAGCAATTGGCGGCCTGTGGCAATGAACACTCGCGGTATTTCACTGCCCGGCAATGG

GCGATCTGCGGCGAGGCCGGATTGCTGGGGCTGTCGATTCCCCGGGAATACGGTGGCCAG

GGCCTGGGTGCACTGTCGACGGCCATTGCCATGCACGCCTTTGGCCTGGGTTGCACAGAC

ATGGGCCTGGTGTTCGCGGCGGCGGCCCACCAGTTTGCCTGTGCGATGCCGATCGTCGAG

TTCGCAACAGCGGAAACCAAACGCGATGTGTTGCCCAAACTCGCCAGCGGTGAATTCATC

GGCTCCAACGCAATCACCGAACCCGAGGCCGGCTCCGACTCCAGCAATTTGAAGAGCCGT

GCCTGGCCCCAGGCCGATGGCAGTTATCGCCTTGACGGCCACAAGAGCTTTGCCGGCAAT

GCGCCGATTGCCGACATCTTCGTGACCTATGCCACCACCCAGCCCGAGTACGGTGCCCTG

GGGGTCAGCGGTTTTATCGTCCACCGCAGCAGTGCGGGGCTCAGGGTCAGTGAGCCCCTG

GACAAGGTATGCCTGAGAAGCTGCCCCGCGGGTGAAGTGTTCTTTGACGATTGCAGGGTT

CCTGAGGTCAACCGCCTGGGTGAGGAGGGGCAGGGCCGGCAGGTGTTCCAGAGCTCCATG

GGCTGGGAGCGTGCCTGCCTGTTCGCAGCGTTCCTGGGGATGATGGAACGGCAACTGGAA

CAGACCATCGAGCATGCGCGCACCCGGCGCCAGTTTGGCAAGCCGATTGGCGACAACCAG

GCGGTTTCGCACCGTATCGCGCAAATGAAGCTGCGCCTGGAGTCGGCGCGGTTGCTGCTG

TTCCGGGCGTGCTGGGGCATGGACCAGGGCGATTCGGGGCAGCTCAACATTGCCCTGTCG

AAACTGGCCATCAGTGAAGGGGCGCTGGCATCGAGCATCGATGCGGTGAGGATTTTCGGC

GGCCGGGGCTGCCTGGAGTCTTTCGGGATCGAGGCGATGCTGCGCGACTCCATCGGCACT

ACGATCTTTTCCGGCACCAGCGACATGCAGCACGAGATCATTGCCCGGGAGCTGAAGCTA

TGA

>pltF fig|380021.185.peg.843 Polyketide synthase modules and related proteins

ATGAAGCTGCTCCATGAACGGATGATGCACAGCCTTGCCCGCTACCCGCGGCAGACGGCG

GTGGTGGATGAGCAGGATGCCTTGAGCTACGAGGCGCTGGAGCTCAGAACCCGGGAATTC

GTGGCAATGCTCTGTGCCCTGGGGGTCGGCCAGGGGCAGCGGATACTGCTCTGGGCGCAC

AAGTCGGTGGACCTGGTGGCGGTCATGCAGGCGGCCCTGCGGCTGGGGGTGGTGTATGTG

CCGGTGGACCCTCTGAGCCCGGTGTCGCGCCTGGAAAAGATCGCCGGGGATTCCCAGGCC

GTGCTGGTCCTCTGCACCGCGGCACGCCAGGAAGAACTCGCCGGCTCCGCGCTTGCCCAG

GTGCGCAGCGTGGTCCTGGACGACCCGGCCAGCGCCGGCTACTGGCGCAACATCGATACC

GGCTCCAGCGTAGTGCCTACGCTGCGCATCCAGCCGGACGATCTGGCCTACATCCTCTAC

ACCTCCGGGTCCACCGGCGTGCCCAAAGGGGTTGCGCTCAGCCACGGCAATGCCCTGGCT

TTCGTCGACTGGGCGTGCGAGCGCTATTGCTTCCAGCCTGGCGAGCGTTTCGCCAACCAT

GCCCCCCTGCATTTCGACCTGTCGGTCCTGGACATCTACTGCGCGCTCAATGTGGGCGCG

ACGGTGTGCCTGGTTCCCGAGTCGATCGCGTTCTCGCCGCGGCTGCTGACCGACTTCATC

CGCCAGCACGAAATCAGCATCTGGTACTCGGTGCCCTCGGTACTCATGATGATGATGCAA

GACGGCGACTTGCTCAGCGATATCCAGGACACCCTGCGGGTACTGTTGTTCGCCGGCGAG

CCTTTTCCGATCAAGCACCTGCGTGACCTGCGCGCGGCCTATGCCGATGTGCGTCTGGCC

AATCTCTTCGGCCCCACGGAAACCAATGTGTGCACCGCATTCGAGGTCGGCGCCATCGAT

CCCGAGCGCGTGCTCCCGGTGCCCATCGGCACGGCCGCCTCCGGCAACCAGGTGTGGGCG

CAAAAGCCTGATGGCAGCCGCTGCGCAGTGGGGGAAGAAGGTGAGCTGGTGGTGCAGGGG

CCTACGGTCATGCTGGGCTATTTCGCCAAGCCGGCTCAGGAGGGGCCCTACAAGACCGGC

GATATGGTCAGGCAGCGGCCTGACGGCAACTACGAATACCTGGGGCGTCGTGACGACATG

CTCAAGGTGCGTGGCAACCGGATCGAGCGCGGGGAAGTCGAAGCCGCGCTGCTGGCCCAT

CCCCAGGTCAGCGAGGCCGCCGTGCTGGTGGTCGGGGAGGGGATGAACGCGCAGTTGTGG

GGCGTGCTGGTCGCTCACACCCGGGACGCTCTTTCGCTGATCGACCTCAAGCGCCACTGC

GCCCAGCGCCTGCCTCGCTACATGATCATCGACAAGGTGCTGTGCCTGGACGCACTGCCA

CGCAACGCCAATGGCAAGGTCGATCGCTTCGCCCTGGCCAGGCAGGTGGAGGGCTGA

>pltG fig|380021.185.peg.844 CFA synthetase, thioesterase component

ATGGGAACTCCATCACGCGTGGCCAAGGACGCCTGGTTTCCCTATGCCAGCCGGCCTCGG

GGCAAGATGCGCCTGTTCACCTTTCCCTATGCCGGGAGCGGGGCCTCGGTCTTTCATCGC

TGGTTCCTGCCCCTGTACGACCAGGTCGACCTGTATGCCTTGCAGTTGCCGGGCCGCGAG

GGTCGCAGCCAGGAGGCCTGCTACAGCGACATGCAGGCGGCGGCCAATGACGTGGCCGAC

TGCCTGGAGCAGTTTGGCGATGACATCCCCTGTTGTTTCTTCGGCCACAGCATGGGCGCC

TTGCTGGCCTTTGCGGTCGCCGGTGTGCTGCAGGAGCGGCGGTTGCCGATGCCGCAGCAA

CTGATGCTCTCGGGCATGGTTGCGCCCCATGTGCGGCAACGGGTAGCACCGCTGCACCAG

CTGCCGGCAGAGCAGGCCATCGCCGCGCTGCAGGCCATGGGGGGCGTGCCGGCGGTGGTG

CTGGCCGAGGAGGACCTGATGCAGATGTACCTGCCTATCATTCAGGCCGACATACAGATG

GTCTACTCCTACGCCGGTGCCCAGCCGCAGCCGCTGGACACCCGCATGATCTGCCTGAGC

GGCGCCCAGGACCTGATTGCGCCGCCTGCGCAGATGCAGCAGTGGCAGCGCTACACCCAG

TGCGGCTTCGAGCAGTTCGTGTTTGCAGGTGGCCATTTCTTTCTCGACGCCCAGGTCATG

TCGCGGGTCAAAACAGTGCTGGGCAGCGCCCTGCACAATCAGCCTGGCAGTCTGGCGGTG

TGA

>pltH fig|380021.185.peg.846 ABC-type efflux pump membrane fusion component YbhG

ATGAAGAAGCAGTTGATTGTAGGGGTTGCCGTACTGCTGGTGGCCACAGCGGGTGTTTCC

TGGTTTCTCCTTCGCCCCGAGAAGCAGAACGACCACCTCAAGCTCCATGGCAACGTCGAC

ATTCGCCAGGTGTCCCTGGCGTTCGACGGCAGTGAGCGAATCGCCGCGCTGTATGCCGAA

GAGGGCGACCTGGTGCAGCCAGGCCAGGTCCTGGCGGAACTCGACACACGCACACTGCGC

CTGGAAATAAATCGCTCCAAGGCCAGGATCGGCGCCCAGGAACAGGCGCTGCTGCGCTTG

AAAAATGGCACACGGCCTCAAGAGGTCGAGCAGTCCAAGGCACGTTTTGATGCCGCCCAG

GCGCAAATGCAGCTGGCTCAACTGCACATGCAGCGCCTGCGCAGGATCGCCGACGACACC

CAGGGCAAGGGCGTCAGCCAGCAACGCCTGGACCAGGCAGCCGCCCGCTTGCAAGTGGCC

AAGGCCCAGTCGCAGGAGCAGCGCGAATCCTGGACCCTGGCCAAGATCGGCCCACGCAAT

GAAGACATCGCCCAGGCCACGGCCGACCTGCAAGCCTCCAGGGCCGACCTGGACCTGCTG

GAACATTACCTGGCGCGCGCTCAGCTCAAGGCGCCGACCCAGGCCCGGATCCGCACGCGC

CTGCTGGAGCCGGGGGATATGGCCTCGCCGCAGCGCCCGGTGTTTGCCCTGGCCCTGACC

GACCCCAAGTGGGTACGGGCCTATGTCAACGAGCGCCAGTTGGGCCACATACGTGCGGAC

CAGATGGCGCGGGTCTACACCGACAGCTTTCCTGACCAGGCCATCGACGGCAAAGTCGGC

TACATCTCCTCGGTTGCCGAATTCACCCCCAAGTCGGTGGAAACGGAAGACCTGCGCACC

AGCCTGGTCTACGAGATCCGGGTGCTGGTCAAGGACCCTGACGATCGCCTGCGGCTCGGT

ATGCCGGCGACCGTCTACCTTGACCAGGCACCGGTGGCCGGGGCCACGCCATGA

>pltZ fig|380021.185.peg.845 Transcriptional regulator YbiH, TetR family

ATGAACACGCGACGCAGAACCAGCAGAAGCGACGGCGAACACACTAAAATCCGCATTCTC

GAAGTGGCTGCCCGCCTGTTTGCCCAGCATGGCTATGCCAACACCGCGAGCAAGCTCATC

TGTGAAGAAGCCGGCGCCGACCTCGCCGCCATCAACTACCACTTCGGTAGCCGGGAAGCC

CTGTACAAGGCGGTGCTGATCGAGGGACACAAGCAGCTGGTGAGCTTCGAAGCCCTGTCG

CAGCTGGCGCAAAGCGAAGAGCCGGCGCTGATCAAGCTCGATAGCTTCATCGATGCCATC

GTCACCCGGGTGCTCGATGAGCAGAGCTGGCAAAGCAAAGTCTGTGCACGGGAAATACTG

GCGCCCACTGTCCATTTCACCAGCCTTGTGCAAGAAGAAGTGATGCCCAAATTCCGTCTG

CTGGAAGCGCTCATCAGCGAGATCACCGGCTTTCCGATCGGTGACCCGGCGCTGGCGCGT

TGCACCATCAGCATCATCGCGCCCTGCCTGATGCTGGCGGTGATAGATCGCCAGCAGCCC

AGCCCGCTGCAAGCCGTCCTGCAACACGACGCCAACGCCCTGAAAGCCCACTTCAAGCTG

TTCGCCAGGTCCGGCCTCGCGGCCATTGCCCAATAG

>pltL fig|380021.185.peg.837 hypothetical protein

ATGGACGGAGAGGAAGTTAAAGAAAAGATTCGTCGCTACATCATGGAAGATTTGATCGGG

CCAAGTGCAAAGGAAGATGAGCTTGATGACCAGACTCCACTACTGGAGTGGGGCATTCTC

AACTCGATGAATATCGTCAAGCTCATGGTCTACATCAGGGATGAAATGGGTGTCTCGATT

CCAAGTACCCATATCACCGGCAAATATTTTAAAGACCTGAATGCAATATCCAGGACTGTC

GAGCAACTAAAGGCCGAGTGCGCCTAA

>pqqA fig|380021.185.peg.4272 Coenzyme PQQ synthesis protein A

ATGTATAGGCAACACCCGTCCCACCCACCTCAAAGGAGCAATTTTATGACCTGGTCCAAA

CCTGCTTACACTGACCTGCGTATCGGCTTCGAAGTCACCATGTACTTCGCCAGCCGCTAA

>prnC fig|380021.185.peg.1697 FAD-dependent oxidoreductase

ATGACTCAGAAGAGCCCCGCGAACGGACACGATAGCAACCACTTCGACGTAATCATCCTC

GGTTCGGGCATGTCCGGTACCCAGATGGGGGCCATCCTGGCCAAACAACAGTTTCGCGTG

CTGATCATCGAGCAGTCGTCGCACCCGCGGTTCACGATCGGCGAATCGTCGATCCCCGAA

ACGTCTCTCATGAACCGCATCATCGCTGATCGCTACGACATTCCGGAGCTCGGCCACATC

ACCTCGTTCTACTCGACGCAGCGTTACGTTTCGTCGAGCACGGGCATCAAGCGCAACTTC

GGCTTCGTGTTCCACAAACCTGGCCAGGAGCACGACCCGAAGGAGTTCACCCAGTGCGTC

ATTCCCGAGCTGCCGTGGGGGCCGGAGAGCCATTATTACCGGCAGGACGTCGACGCCTAT

CTGTTGCAAGCGGCCATCAAATATGGCTGCACGGTCCGCCAGAAGACGAGCGTGACCGAA

TATCACGCGGACAAGGACGGCGTCGCGGTGACCACCGCCGAGGGCGAGCGGTTCACCGGC

CGGTACATGATCGACTGCGGAGGACCCGGCGCGCCGCTGGCGACCAAGTTCGGGCTCCGC

GAAGAGCCGTGTCGCTTCAAGACGCACTCGCGCAGCCTCTACACGCACATGCTCGGGGTC

AAGCCGTTCGACGACATCTTCAAGGTCAAGGGGCAGCGCTGGCGCTGGCACGAAGGAACC

CTGCACCACATGTTCACGGGCGGCTGGCTCTGGGTGATTCCGTTCAACAACCACCCGCGC

TCGACCAATAACCTGGTGAGCGTCGGCCTGCAGCTCGACCCGCGTGTCTACCCGAAAACC

GACATTCCCGCGCAGCAGGAATTCGACGAGTTCCTCGCGCGGTTCCCGAGCATCGGCGCT

CAGTTCCGGGACGCCGTGCCAGTGCGCGACTGGGTCAAGACCGACCGCCTGCAGTTCTCG

TCGAACGCCTGCGTCGGCGACCGCTACTGCCTGATGCTGCACGCGAACGGGTTCATCGAC

CCGCTCTTCTCCCGGGGGCTCGAGAACACCGCGGTGACCATCCACGCGCTCGCGGCGCGC

CTCATCAAGGCGCTACGCGACGACGACTTCTCCCCCGAGCGCTTCGAGTACATCGAGCGC

CTGCAGCAAAAGCTTTTGGACCATAACGACGACTTCGTCAGCTGCTGCTACACGGCGTTC

TCGGACTTCCGCCTATGGGACGCGTTCCACCGGCTGTGGGCGGTCGGCACTATCCTCGGG

CAGTTCCGGCTGGTGCAAGCCCACGCGAGGTTTCGCGCGTCGCGTGACGAGGGCGACCTC

GATCACCTCGACAACGACCCGCCGTACCTCGGGTACCTGTGCGCGGACATGGAGCAGTAC

TACCAGTTGTTCAACGACGCCAAAGCCGAGGTCGAGGCCGTGAGCGCCGGGCACAAGTCG

GCCGAGGAGGCCGCGTTGCGGATTCACGCCCTCATCGACGAACGAGACTTCGCCAAGCCG

ATGTTCGGCTTCGGGTACTGCATCACCGGGGACAAGCCGCAGCTCAACAACTCGAAGTAC

AGCCTGATACCGGCGATGAAGCTGATGTACTGGACGCAAACCCGCGCGCCGGCAGAGGTG

AAGAAGTACTTCGACTACAACCCGATGTTCGCGCTGCTCAAGGCGTACATCACCACCCGC

ATCGGCTTGGCTCTGAAGAAGTAG

>trpA fig|380021.185.peg.4888 Tryptophan synthase alpha chain (EC 4.2.1.20)

ATGAGCCGCCTGCAAACCCGTTTTGCCGAGCTGAAGGAAGCAAACCGCGCCGCCCTGGTA

ACCTTCGTCACCGCCGGCGACCCGAACTACGACACCTCCCTGGCGATCCTCAAGGGCCTG

CCCGCAGCGGGCGCCGACGTGATCGAACTGGGCATGCCCTTCACCGACCCGATGGCCGAC

GGCCCGGCGATCCAGCTGGCCAACATCCGTGCCCTGGGGGCCAAGCAAAACCTGGCGAAA

ACCCTGCAGATGGTTCGCGAGTTCCGCGAAGGCAACAGCGACACCCCGCTGGTGCTCATG

GGCTACTTCAACCCGATCCACCACTACGGCGTGCCGCGCTTCATCGCCGACGCCAAGGCC

GCCGGCGTCGATGGCCTGATCGTGGTGGACATGCCGCCGGAACATAACAGCGAACTGTGC

GACCCGGCCCAGGCCGCCGGCATCGACTTCATCCGCCTGACCACACCGACCACCGACGAC

GCCCGCCTGCCCAAGGTGCTCAACGGCAGCTCCGGTTTCGTCTACTACGTTTCCGTGGCC

GGGGTCACCGGTGCCGGTGCCGCCACCCTGGAACACGTGGAAGAAGCCGTGGCCCGCCTG

CGTCGCCACACCGACCTGCCCATCAGCATCGGTTTCGGCATCCGTACTCCGGAGCAGGCA

GCCGCCATCGCTCGCCTGGCCGACGGCGTGGTAGTGGGCTCGGCGCTGATCGACCACATC

GCCAGCGCCGAGTCTGCCGAGCAGGCGGTGGATGGGGTGCTGAGCCTGTGTGCGGCGCTG

TCCGAGGGCGTACGTAAGGCCCGCGTCAGCTGA

>trpB fig|380021.185.peg.4889 Tryptophan synthase beta chain (EC 4.2.1.20)

ATGACCCAGTCCCAATTACGCAATGGCCCTGACGACAACGGCCTGTTCGGCGCATTCGGC

GGCCGTTACGTGGCCGAAACCCTGATGCCGCTGATCCTCGACCTGGCCCGCGAATACGAA

AAGGCCAAGGAAGATCCCGAATTCCTCAAGGAGCTGGCCTACTTCCAGCGCGATTACGTG

GGCCGCCCCAGCCCGCTGTACTTCGCCGAACGCCTGACCGAGCACTGCGGCGGCGCCAAG

ATCTACCTCAAGCGTGAAGAGCTGAACCACACCGGCGCGCACAAGATCAACAACTGCATC

GGCCAGATCCTGCTGGCGCGGCGCATGGGCAAGAAACGCATCATCGCCGAGACCGGCGCC

GGCATGCATGGCGTGGCCACCGCCACCGTGGCCGCGCGTTTTGGCCTGGATTGCGTGATC

TACATGGGCACCACCGACATCGAGCGCCAACAGGCCAACGTATTTCGCATGAAGCTGCTG

GGCGCCGAAGTGATCCCGGTGGTGGCCGGCACCGGCACCCTGAAGGACGCGATGAATGAA

GCCCTGCGTGACTGGGTGACCAACGTCGACAGCACCTTCTACCTGATCGGCACCGTCGCC

GGCCCGCACCCGTACCCGGCCATGGTCCGCGACTTCCAGGCCGTGATCGGCAAGGAGACC

CGCGAGCAGATGCAAGCCCAGGAAGGCCGCCTGCCGGACAGCCTGGTGGCGTGCATCGGT

GGCGGTTCCAACGCCATGGGCCTGTTCCACCCGTTCCTTGACGACAAGAGCGTCGAGATC

ATCGGCGTCGAAGCCGCCGGCTACGGCATCGAGACCGGCAAGCACGCCGCCAGCCTCAAC

GGCGGCGTACCCGGCGTACTGCACGGCAACCGCACCTTCCTGCTGCAGGACGACGATGGC

CAGATCATCGACGCCCACTCGATTTCCGCCGGCCTCGACTACCCCGGCATCGGCCCGGAA

CACGCCTGGTTGCACGATATTGGCCGCGTCGAATACACCTCGGTGACCGACGACGAAGCC

CTGGCCGCGTTCCACCAGTGCTGCCGCCTGGAAGGCATCATCCCGGCCCTGGAAAGCGCC

CATGCCCTGGCCGAAGTCTTCAAGCGCGCACCGAACCTGCCCAAGGATCACCTGATGGTG

GTCAACCTCTCGGGCCGTGGCGACAAAGACATGCAAACCGTGATGCACCACATGCAACAG

TCCCAGCAGGAGAAACACTGA

>trpC fig|380021.185.peg.4220 Indole-3-glycerol phosphate synthase (EC 4.1.1.48)

ATGAGTGTGCCGACAGTTCTGGAAAAAATCCTCGCCCGTAAAGCCGAGGAGGTTGCCGAG

CGCAGCGCCCGCGTCAGCCTGGCCGAGCTGGAAACCCTGGCGCGTGCTGCCGATGCACCA

CGGGGTTTCGCCCGGGCCTTGCAGGATCAGGTCAAGCTCAAGCAGCCGGCAGTGATAGCC

GAGATCAAGAAGGCTTCGCCGAGCAAAGGCGTGATCCGTGAGAACTTCGTTCCGGCGGAC

ATTGCCAAGAGCTACGAGAAAGGCGGTGCCACTTGTCTGTCCGTGCTGACCGATGTCGAT

TACTTCCAGGGTGCCGATGCGTACCTGCAGCAGGCCCGCGCCGCCTGCAAGCTGCCGGTG

ATCCGCAAGGACTTCATGATCGACCCGTACCAGATCGTCGAAGCCCGTGCCCTGGGCGCC

GACTGCGTATTGCTGATTGTCGCTGCGCTGGATGACGCGCGGATGGCGGAGCTGGCGGCG

TTGGCCAAGGGCGTTGGCCTGGATGTGCTGGTGGAAGTGCATGATGGTGATGAGCTGGAG

CGGGCGTTGAAGACCCTCGACACACCGCTGGTTGGGGTCAACAACCGCAACCTGCACACC

TTCGAAGTCAATCTGGAGACCACCCTCGACCTGCTGCCACGCATTCCCCGCGAGCGCCTG

GTGATTACCGAGAGCGGCATCCTCAACCGTGCCGATGTCGAGCTGATGGAAATCAGCGAT

GTCTATTCGTTCCTGGTGGGTGAGGCGTTCATGCGTGCCGAAAGCCCGGGCAGCGAATTG

CAGCGTCTGTTCTTCCCTGAGCGCGGCGTGCCGGTCAGCGGCTCGACCCTGGACTGA

>trpD fig|380021.185.peg.4221 Anthranilate phosphoribosyltransferase (EC 2.4.2.18)

ATGGATATCAAGACTGCCTTGAGCCGTATCGTCGGCCACCTGGACCTGAGCACCGAAGAA

ATGCGTTCGGTAATGCGTGAAATCATGACCGGCCAATGCACCGATGCGCAGATTGGCGCC

TTCATGATGGCCATGCGCATGAAAAGCGAAAGCATCGACGAGATCGTCGGTGCGGTATCG

GCAATGCGCGAGCTGGCGGACCGGGTCGAGCTGAAAACCCTGGATGGCGTGGTGGATGTA

GTGGGCACCGGCGGCGACGGGGCGAACATCTTCAACGTGTCCACGGCTTCCTCCTTTGTG

GTCGCAGCGGCCGGTTGCACTGTGGCCAAGCACGGCAACCGCGCGGTGTCCGGCAAGAGC

GGCAGCGCCGACCTGCTGGAAGCGGCAGGGATCTATTTGAACCTGACCCCGGTCCAGGTG

GCGCGCTGCATCGATAACGTCGGCATCGGCTTCATGTTTGCCCAGTCCCATCACGGGGCG

ATGAAATATGCCGCCGGCCCGCGTCGCGACCTTGGCCTGCGCACCCTGTTCAACATGCTC

GGCCCGCTTACGAATCCGGCCGGAGTCAAGCATCAGGTGGTAGGCGTGTTCACTCAGGCG

TTGTGCCGGCCCTTGGCCGAAGTCTTGCAACGCCTGGGCAGCAAGCACGTTCTGGTGGTG

CATTCCCAGGATGGTCTGGACGAGTTCAGCCTGGCTGCGCCCACCTATGTGGCCGAGCTG

AAGAATGACCAGATCACCGAGTACTGGGTACAGCCCGAGGATCTGGGCATGAAGAGCCAG

AGTCTGTTCGGCCTGGTGGTGGAAAGCCCGGCGGCCTCCCTGGAGCTGATCCGCGATGCC

CTGGGGCGCCGCAAGACCGAGCATGGCCAGAAGGCCGCCGAAATGATTGTGCTCAATGCG

GGTGCGGCGCTGTATGCCGCCGATCATGCCAGTAGCCTGAAAGAGGGCGTGGCCCTGGCT

CACGACGCCTTGCACACCGGCCTGGCCCGGGAGAAGCTCGAAGAGCTGGGTGCCTTTACC

GCGGTGTTCAAGCAGGAGAATGAAGGATGA

>trpE fig|380021.185.peg.4228 Anthranilate synthase, aminase component (EC 4.1.3.27)

ATGATTCGCGAAGAATTCCTGCGTTTGGCCGCAGCCGGTTACAACCGTATCCCGCTGGCC

TGCGAAACCCTTGCCGACTTCGACACCCCGCTGTCGATCTACCTGAAACTGGCCGACCAG

CCCAACTCCTATCTGCTGGAGTCGGTGCAGGGCGGTGAGAAGTGGGGGCGTTACTCGATC

ATCGGCCTGCCGTGCCGCACTGTGCTGCGGGTGCACGGCTTCCAGGTCAGCGTGACCCAT

GATGGCGTGGAGATCGAAAGTCACGAAGTGGCCGACCCGCTGGCCTTCGTCGAGGAATTC

AAAGCCCGCTACAACGTCCCGACCATTGCCGGCCTGCCGCGTTTCAACGGTGGCCTGGTG

GGCTATTTCGGCTATGACTGCGTGCGTTATGTCGAGCCGCGCCTGGGCCCGTGCCCGAAC

CCGGATCCGATCGGTGTGCCGGATATCCTGTTGATGGTCTCCGACGCGGTAGTGGTGTTC

GACAACCTGGCGGGCAAGGTGCACGCCATCGTGCTGGCCGACCCTGGCCAGGACAACGCC

TTCGAAGCCGGCCAGGCACGTTTGCAGGAATTGCTGGCGCAACTGCGCCAGCCGATCACT

CCGCGTCCGGGCCTGGATTTCAGCAAGCAGCTGGCCGCTGATCCGCTGTTCCGCTCCAGC

TTTACCCAGGCTGACTATGAGCGGGCCGTGGACACCATCAAGGAATACATCCTGGCCGGT

GACTGCATGCAGGTGGTGCCGTCCCAGCGCATGTCCATCGATTTTTCGGCGGCGCCCATC

GACCTGTACCGGGCGCTGCGCTGCTTCAACCCGACCCCTTACATGTACTTCTTCAACTTC

GGTGATTTCCACGTAGTGGGCAGCTCGCCGGAAGTGCTGGTGCGGGTCGAAGACAACCTG

ATCACTGTGCGGCCGATCGCCGGTACCCGCCCGCGGGGCGCCTCCGAGGAGGCCGACCTG

GCCCTGGAGCAGGACCTGCTGTCGGACGCCAAGGAGATCGCCGAGCACCTGATGCTGATC

GACCTGGGACGCAACGACACCGGTCGGGTCTCGGAAATCGGTTCGGTGAAACTCACCGAA

AAGATGGTCATCGAGCGCTATTCCAACGTGATGCACATCGTTTCCAACGTCACCGGCCAG

CTCAAGGCCGGGCTGACGGCCATGGATGCGCTGCGGGCGATCCTGCCGGCGGGCACTCTG

TCCGGCGCACCGAAAATCCGTGCGATGGAAATCATCGACGAGCTGGAACCGGTCAAGCGT

GGGATCTATGGCGGCGCCGTGGGTTACTTCGCCTGGAACGGCAACATGGACACCGCCATT

GCCATTCGCACGGCGGTGATCAAGAACGGCGAGCTGCATGTACAGGCCGGCGGCGGGATT

GTTGCCGACTCGGTGCCGACCCTGGAGTGGGAAGAGACGCTGAACAAGCGTCGCGCAATG

TTCCGTGCCGTGGCACTGGCGGAGCAGACCCCTGCATCCAAGGCTTGA

>trpF fig|380021.185.peg.17 Phosphoribosylanthranilate isomerase (EC 5.3.1.24)

ATGCCAGCTGTTCGCAGCAAGATCTGCGGGATTACCCGCATAGAAGATGCGTTGGCAGCA

GTGGCGGCGGGGGCCGATGCCATCGGCCTGGTGTTCTATGCCAAGAGCCCGCGGGCGGTG

AATGTGCAGCAGGCGCGGGCAATCATCGCCGCCTTGCCGCCGTTCGTGACCAGCGTCGGC

CTCTTCGTCAATGCCAGTCGTTGCGAGCTGGGGGAGATTCTCGACGCCGTACCGCTGGAC

TTGCTGCAGTTTCATGGCGATGAGTCGGCGGCTGATTGCGAGGGCTATCACCGGCCCTAT

ATCAAGGCGTTGCGGGTCAAGGCCGGGGATGACATCGCGGCGGCCTGCCTGGCCTATCCG

CGGGCCAGCGGGATTCTGCTGGATACCTATGTCGAAGGCGTGCCGGGTGGAACCGGCGAG

GCGTTCGACTGGTCCCTGGTTCCTCAGGGGCTGAGCAAGCCGATCATTCTTGCCGGTGGC

CTCACGCCAGATAACGTGGCGGCAGCCATCGCCCGGGTGCGCCCGTATGCGGTGGATGTC

AGCGGCGGGGTGGAGCAGGGCAAGGGCATCAAGGACCCGGCAAAGATTCAGGCATTTATG

CAGGCAGTGCGTCGCAGCAACGAGTCGATGTGA

>trpG fig|380021.185.peg.4222 Anthranilate synthase, amidotransferase component (EC 4.1.3.27) @ Para-aminobenzoate synthase, amidotransferase component (EC 2.6.1.85)

ATGCTGCTGATGATTGATAACTACGACTCCTTCACCTACAACGTCGTCCAGTACCTGGGC

GAGTTGGGAGCTGAGGTGAAAGTAGTGCGCAATGACGAACTGACCGTGGCTCAGATCGAG

GCGCTCAAGCCCGAGCGCATCGTGGTCTCGCCCGGGCCTTGCACGCCGACCGAAGCCGGT

ATTTCCATCGAGGCGATCAAGCATTTCGCCGGCAAGCTGCCAATTCTCGGCGTATGCCTG

GGGCATCAGTCCATCGGCCAGGCCTTTGGCGGCCAGGTGGTGCGTGCCCGGCAAGTGATG

CACGGCAAGACCAGCCCGGTGTTCCACCGTGACCTGGGGGTGTTCAACGGCCTGAACATG

CCGCTGACCGTAACCCGTTACCACTCGCTGGTGGTCAAGCACGAGAGCTTGCCCGAGTGC

CTCGAAGTCACGGCCTGGACCCAGCTGGAAGACGGCTCCGTCGACGAGATCATGGGGTTG

CGCCACAAGACGCTGAATATCGAGGGGGTGCAATTTCACCCCGAGTCTATTCTCACCGAG

CAGGGCCACGAGCTGTTCGCCAACTTTCTCAAACAGAGCGGCGGCACGCGCTAA
